# Tumors hijack immune-privileging regulons via distinct cell types to confer T cell desertion and immunotherapy resistance across various cancers

**DOI:** 10.1038/s41467-026-72538-x

**Published:** 2026-05-08

**Authors:** Bashir Lawal, Akshat Gupta, Renu Sharma, Huayan Ren, Rohit Bhargava, Yue Wang, Xiao-Song Wang

**Affiliations:** 1 UPMC Hillman Cancer Center, University of Pittsburgh, Pittsburgh, PA USA; 2 Department of Pathology, University of Pittsburgh, Pittsburgh, PA USA; 3 Magee-Womens Hospital of UPMC, Pittsburgh, PA USA

**Keywords:** Cancer immunotherapy, Cancer microenvironment

## Abstract

Immune checkpoint blockade (ICB) has transformed oncology, yet most patients fail to respond, suffer from hyper-progressive disease, or face severe immune-related toxicities, underscoring the urgent need for biomarkers that identify non-responders. Here we show that tumors co-opt an immune-privileging regulon signature (IMPREG) mirroring transcriptional programs of immune-privileged organs — to enforce T-cell desertion and ICB resistance across solid tumor types. Single-cell and spatial transcriptomic analyses reveal that tumors activate IMPREG through three distinct cellular routes: malignant cells adopting immature neuronal states, cancer-associated fibroblasts assuming myofibroblast identities, or endothelial cells — each creating localized niches of immune suppression and antigen-presentation collapse. Across 4 discovery and 36 validation clinical datasets, IMPREG consistently predicts immunotherapy resistance in 14 distinct cancer types, functioning as an orthogonal marker independent of established biomarkers. Crucially, IMPREG-expressing tumors show enhanced sensitivity to EGFR inhibitors or anti-angiogenic therapies in specific tumor entities. These findings suggest IMPREG as a dual-utility predictive biomarker for personalized treatment stratification.

## Abstract (web summary)

Immune checkpoint blockade has revolutionized cancer therapy, yet resistance and immune-related toxicities remain major challenges. This group reveals that tumors hijack immune-privileging regulons (IMPREG) via distinct cell types to confer T-cell desertion, and validates IMPREG as an orthogonal pan-cancer biomarker predicting both immunotherapy resistance and sensitivity to specific targeted therapies.

## Introduction

The advent of cancer immunotherapy, particularly ICB therapies targeting PD-1 and CTLA-4, has revolutionized cancer treatment^1–3^. However, this promise is tempered by a critical reality: a vast majority of patients derive no clinical benefit, with durable responses observed in only about 20% of cancer patients^4–7^. For non-responders, the consequences are not just a lack of efficacy but the potential for significant harm, as 5–29% experience hyper-progressive disease (HPD), a rapid acceleration of tumor growth that worsens prognosis. Furthermore, 10–43% of all patients undergoing ICB develop severe, life-threatening immune-related adverse events (irAEs). For example, in non-small cell lung cancer (NSCLC)^8^, ICB is a cornerstone of first-line therapy, but its use presents a significant clinical dilemma. While offering durable benefits, the objective response rates remain below 50%. This challenge is compounded by hyper-progressive disease^9^ affecting up to 15% of patients, and the incidence of severe irAEs (Grade ≥ 3) ranges from 5–10% with monotherapy to as high as 35% with dual ICB inhibitors^10^. A similar challenge is acute in triple-negative breast cancer (TNBC): adding anti-PD-1 to chemotherapy offers only a 12–13.6% improvement in responses^11^, whereas 20–25% of patients suffer severe irAEs^12,13^.

Given these challenges, early and reliable identification of patients likely to benefit from ICB therapy is crucial to reduce adverse outcomes and improve therapeutic efficacy^14–16^. Many biomarkers have been developed to predict therapeutic sensitivity, including PD-L1 expression, tumor mutational burden (TMB), and other genomic markers linked to neoantigens. Our group has identified predictors such as intragenic rearrangement burden and tumor-associated antigen burden to identify responders with low TMB and PD-L1-negative tumors^17,18^. However, the high prevalence of irAEs and HPD underscores the urgent need for robust biomarkers to identify patients at risk of resistance. More critically, the financial burden associated with ICB therapies remains substantial, with single-agent annual costs typically ranging from $100,000 to $150,000. The widespread use of ICB in non-responding patients creates a staggering economic burden in the U.S., exceeding $20 billion annually, based on an overall 80% non-response rate^19^. This highlights the critical need for robust biomarkers to avoid futile treatments and select effective therapeutic alternatives.

Tumor resistance to T-cell infiltration presents a major obstacle for ICB therapy, frequently resulting in immune-desert or immune-excluded microenvironments where T cells are confined to the stroma. These tumors mimic naturally immune-privileged organs (such as the brain, retina, and testis) that evade systemic immunity to preserve specialized functions^20^. While it was conventionally thought that blood-tissue barriers employ tight junctions to physically restrict T-cell infiltration^21^, recent studies identify specific molecular mechanisms that contribute to immune privilege. Key mechanisms include downregulation of MHC expression, which limits antigen presentation^22^, and cytokine signaling, particularly TGF-β, which suppresses T-cell activation while promoting regulatory T-cell differentiation^23,24^. Furthermore, the expression of PD-L1 and FasL on resident cells fosters tolerance^25^, and induces apoptosis in infiltrating Fas+ lymphocytes respectively^26^. Collectively, these mechanisms establish a tolerogenic environment that balances immune surveillance with tissue preservation.

Transcription factors (TFs) play pivotal roles in gene regulation, orchestrating various cellular programs by controlling gene expression^27,28^. Dysregulations of transcriptional programs are known to prime the initiation, progression, and prognosis of solid tumors^29–33^. Some tumorigenic TFs, such as HIFs, Myc, ETS-1, β-catenin, and TP53, also play significant roles in immune evasion, and recent studies have highlighted the regulatory role of specific TFs in immune modulation and immune checkpoint expression^34–37^. Transcriptional regulons offer a more precise reflection of TF activity than TF expression^38^. A regulon is a collection of genes regulated as a unit by the same transcription factor acting as a repressor or activator. A recent study mapped immune-related regulons across multiple cancer types, revealing a diverse landscape associated with immune cell infiltration, yet their relevance to immunotherapy response remains unexplored^39^. Another focused study on a myeloid cell-related regulon underscored its predictive value for ICB response^40^. This calls for a broader exploration of transcriptional regulons that confer ICB resistance in distinct cellular compartments within tumors.

In this study, we analyzed human transcription factor regulons in a large panel of ICB clinical trial datasets^41–43^, and identified an immune-privileging regulon signature (IMPREG) shared by immune-deserted tumors and privileged tissues. IMPREG functions as a unique, orthogonal biomarker of immunotherapy response, distinct from canonical biomarkers, and provides dual utility by predicting both ICB resistance and sensitivity to specific targeted therapies.

## Results

### Large-scale analysis of regulon activities in clinical trial datasets revealed an immune-privileging regulon (IMPREG) signature predictive of ICB response

**Fig. 1:**
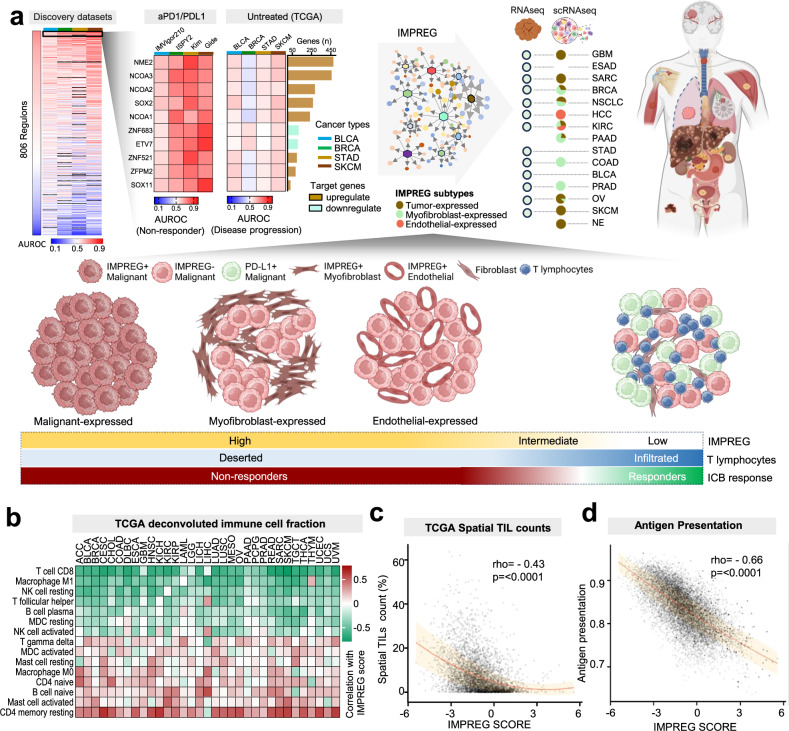
Identification of the immune-privileging regulons linked to tumor immune evasion and ICB resistance. **a** Discovery of the IMPREG signature and its relationship to TME subtypes. The heatmap (left) shows AUROC scores of 806 regulons across discovery datasets, highlighting 10 regulons that most predict ICB therapy resistance in breast (BRCA), melanoma (SKCM), bladder (BLCA), and stomach (STAD) cancers (middle panel). AUROC analysis of disease progression in untreated TCGA cohorts validates their predictive value (right panel). The bar graph depicts the number of target genes of each regulon, categorized by their regulation mode. The bottom schematic highlights the TME subtypes of IMPREG+ tumors linked to T cell desertion and ICB resistance. The illustrations were created using BioRender (Lawal, B. (2026) https://BioRender.com/34p48c7, and Wang, X. (2026) https://BioRender.com/hjyx97z). **b** Spearman correlations between the IMPREG scores and the immune cell fractions deconvoluted from TCGA pan-cancer data using CIBERSORT. **c** Scatter plots showing two-sided Spearman correlations between IMPREG and spatial TIL counts (*n* = 4917, independent tumor sample, rho = −0.43) in TCGA cohorts derived from deep learning analysis of histopathological images. Shaded bands represent smoothed residual envelopes around the fitted regression line. **d** Scatter plots showing two-sided Spearman correlations between IMPREG scores and antigen presentation proficiency (*n* = 9860, independent tumor sample, rho = −0.66) in the TCGA dataset. Shaded bands represent smoothed residual envelopes around the fitted regression line. Source data are provided as a Source Data file.

To identify transcriptional regulons predictive of patient responses to anti-PD-1 immunotherapy, we conducted a comprehensive analysis of human transcription factor activity using Dorothea, a robust and widely accepted tool for assessing regulon activity^43^. Regulon activities were computed based on the expression levels of downstream target genes alongside their modes of regulation. The workflow is depicted in Supplementary Fig. 1. To mitigate potential biases arising from unequal sample sizes, we employed a consensus discovery approach where each of the four diverse, high-quality cohorts was analyzed independently to ensure that larger datasets did not dominate the derived signature (see “Methods”). Regulons were selected based on consistent association with non-response in ≥3/4 discovery datasets (*p* < 0.05, no reverse predictions) and average AUROC ≥ 0.7. This approach revealed a unique set of ten regulons consistently linked to resistance to anti-PD-1/PD-L1 therapies in multiple cancer types, including stomach adenocarcinoma (STAD, Kim cohort)^44^, melanoma (Gide cohort)^45^, breast cancer (BRCA, ISPY2 trial)^46^, and bladder cancer (BLCA, IMvigor210 cohort)^47^ (Fig. 1a, Supplementary Fig. 2).

To evaluate the prognostic effects of these regulons, we analyzed their associations with disease progression within untreated cancer patient cohorts from TCGA (Fig. 1a). Our findings reveal that these regulons are not broadly indicative of poor clinical outcomes, suggesting their predictive role for ICB resistance. Next, we developed a composite signature, referred to as the IMPREG signature, by averaging the activities of the ten identified regulons. The IMPREG signature demonstrated strong predictive accuracy across several cancer types, with an area under the ROC curve (AUROC) of 0.87 in STAD, 0.83 in melanoma, and 0.79 in BRCA, and a moderate AUROC of 0.62 in BLCA. Survival analyses revealed a pronounced association between high IMPREG scores and reduced overall survival (OS) in the melanoma Gide cohort (HR = 3.56, *p* = 0.002) and the BLCA cohort (HR = 1.36, *p* = 0.04) (Supplementary Fig. 2).

### IMPREG signature dictates an immunosuppressive TME via multifaceted mechanisms

We then investigated the impact of IMPREG on the tumor microenvironment (TME) by analyzing immune cell fractions deconvoluted using CIBERSORT^48^. Our analysis revealed a strong negative correlation between IMPREG score and key anti-tumor immune cell populations, including CD8 + T cells, M1 macrophages, follicular helper T cells, while showing a positive correlation with mast cells and CD4 memory resting T cells. This trend was consistently observed across all TCGA cancer types (Fig. 1b). This observation was further validated using spatial immune cell quantification of histopathological images^49^. IMPREG shows a strong inverse correlation with TILs (Fig. 1c) and a positive correlation with neutrophils, mast cells, and macrophages (Supplementary Fig. 3a). We next explored the mechanistic role of the IMPREG signature in immune modulation. IMPREG inversely correlates with pro-inflammatory cytokines, chemokines, and immune markers, as well as TCR/BCR diversity (Supplementary Fig. 3b, c). Notably, IMPREG exhibits a strong negative correlation with antigen processing and presentation signatures (rho = -0.66, Fig. 1d), a trend that persists even in tumors with high TIL levels (Supplementary Fig. 4). These results underscore the multifaceted mechanisms by which IMPREG orchestrates an immune-evasive TME.

### IMPREG is expressed in immune-privileged organs and immunosuppressive cell clusters

**Fig. 2:**
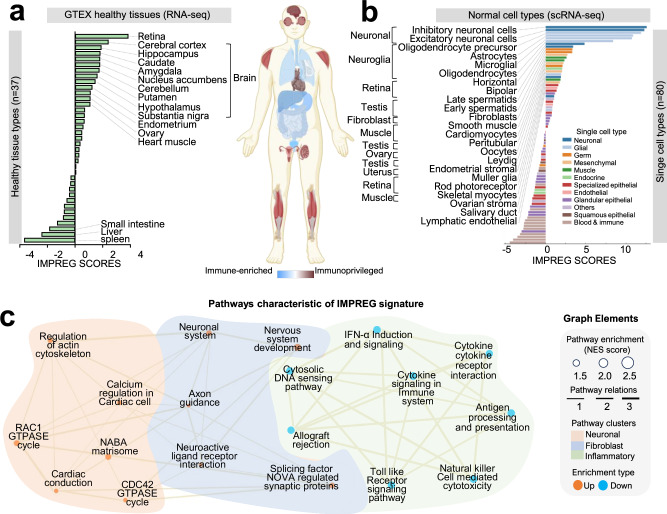
IMPREG expression in immune-privileged organs and immunosuppressive cell types highlights a physiological immune evasion mechanism exploited by tumors. **a** IMPREG expression across 37 healthy human tissue types, analyzed using bulk RNA-seq data from the GTEx Project. The schematic illustrates the anatomical distribution of immune-privileged (red) and immune-enriched (blue) tissues, highlighting the tissue-specific role of IMPREG in immune privilege. The anatomical schematic in panel (**a**) was created in BioRender (Lawal, B. (2026) https://BioRender.com/0m98mew). **b** Cell-type-specific IMPREG expression across 80 distinct cell types, derived from scRNA-seq data across 31 studies in the Human Protein Atlas (HPA). IMPREG over-expression is predominantly in immune-privileged cell clusters but is absent in blood and immune cell populations. **c** In-depth pathway enrichment analysis conducted on the target gene sets associated with the IMPREG signature identifies distinct clusters of functionally related pathways. Node size corresponds to normalized enrichment scores (NES), with pathways grouped into clusters based on shared functions. Pathway enrichment was performed using MSigDB v2022.1 Hallmark and Canonical Pathways C2 gene sets, with significance defined by FDR < 0.05. Source data are provided as a Source Data file.

To elucidate the physiological role of IMPREG, we analyzed RNA-seq data from healthy human tissues obtained from the GTEx project. This revealed that IMPREG expression is markedly elevated in immune-privileged tissues, such as the retina, the brain, and the central nervous system (CNS). Additionally, IMPREG is also modestly upregulated in the ovary, endometrium, and heart. Conversely, it is conspicuously absent in T cell-rich organs such as the spleen, liver, and small intestine (Fig. 2a). These results were corroborated by scRNA-seq data from diverse healthy human cell types, showing that IMPREG is predominantly expressed in immune-privileged cell clusters, such as neuronal cells, retinal cells, spermatids, and fibroblasts (Fig. 2b). This tissue-specific expression pattern strongly suggests that IMPREG functions as a transcriptional program driving immune-privileged TME.

Using our IndepthPathway tool^50,51^, we performed pathway enrichment analysis of the IMPREG target genes based on the known mode of regulation. This analysis revealed that IMPREG defines a specific “immune-privilege” program characterized by upregulation of neuronal pathways (e.g., nervous system development) and stromal-vascular remodeling (Fig. 2c). In contrast, immune activation pathways, including interferon responses, cytokine signaling, and antigen presentation and processing, were negatively regulated by IMPREG. These data suggest that IMPREG may promote an immune-privileged environment by enhancing pathways typical of immune-exempt tissues.

### Tumors hijack immune-privileging regulons via distinct cellular compartments

**Fig. 3:**
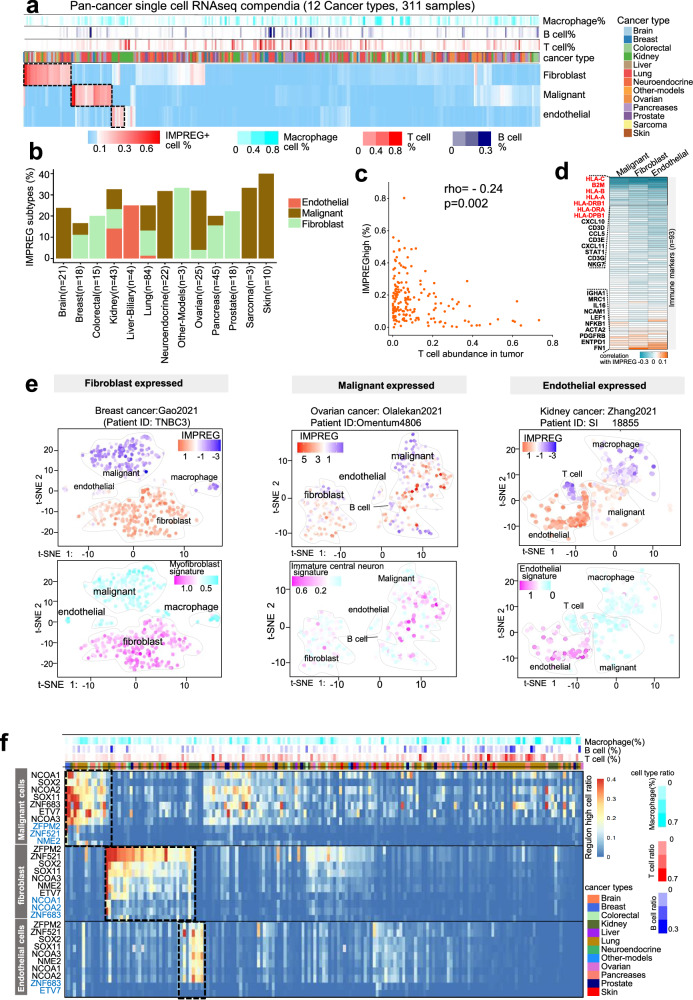
Tumors hijack immune-privileging regulons via distinct cell compartments in the TME. **a** Heatmap depicting the cellular distribution of high IMPREG expression across major tumor microenvironment compartments in pan-cancer single-cell RNA-sequencing (scRNA-seq) datasets. Each column represents a cancer type, and each row denotes a cell lineage, illustrating compartment-specific enrichment of the IMPREG program. **b** Stacked bar charts summarizing the relative frequency of IMPREG subtypes, classified by the dominant cellular compartment expressing IMPREG (malignant-cell–driven, fibroblast-driven, or endothelial-driven), across multiple cancer types using all samples in the dataset. Hematological malignancies were excluded to prevent skewing towards the malignant-driven subtype. **c** Scatter plots showing Spearman correlations between IMPREG scores and T-cell abundance across tumor samples (*n* = 175, rho = −0.24, *p* = 0.002), indicating immune exclusion in IMPREG-high tumors. **d** Heatmap illustrating the Spearman correlation coefficients between IMPREG expression and a curated panel of 93 immune marker genes within different IMPREG-high cell compartments. **e** Representative t-SNE projections from selected tumors demonstrating the spatial and cellular localization of IMPREG-high cells within malignant, fibroblast, or endothelial populations, corresponding to the three IMPREG subtypes. **f** Heatmap showing individual IMPREG transcriptional regulon signature-high cell fractions across various cell types in single-cell datasets covering multiple cancer types. Each column represents an individual sample, and each row denotes a cell lineage, illustrating compartment-specific enrichment of the IMPREG program. Panels (**c**–**f**) used solid-tumor samples filtered for malignant + (endothelial and/or fibroblast) representation and Shannon diversity ≥ 0.8, whereas panels (**a**, **b**) used all solid tumor samples to ensure the figures reflect the true landscape of cancer subtypes, not just the “high-diversity” ones. All correlation analyses were performed using Spearman’s rank correlation. Source data are provided as a Source Data file.

To elucidate the cellular origins of the IMPREG signature within the tumor microenvironment (TME), we analyzed a pan-cancer scRNA-seq compendium of 366 subjects across 40 datasets^52^ (311 solid tumor samples after excluding hematological malignancies and low-cell-count samples). We found that high IMPREG expression originates from one of three distinct cellular compartments—malignant, fibroblast, or endothelial cells—depending on the tumor type (Fig. 3a, Supplementary Fig. 5). The distribution of these IMPREG subtypes varies significantly across cancer types; for example, the malignant-expressed subtype is dominant in brain and neuroendocrine cancers, the fibroblast-expressed subtype in breast and pancreatic cancers, and the endothelial-expressed subtype in kidney cancer (Fig. 3b).

Crucially, high IMPREG expression is significantly associated with T-cell exclusion across tumors (rho = -0.24, *p* = 0.002), suggesting a direct role in creating an immune-deserted TME (Fig. 3c). Mechanistically, IMPREG is linked to the widespread suppression of anti-tumor immunity. IMPREG is strongly anti-correlated with genes essential for both MHC-I (e.g., HLA-A/B/C, B2M) and MHC-II (e.g., HLA-DRA/DRB1) antigen presentation, as well as T-cell receptor components (i.e., CD3D/E). Conversely, IMPREG positively correlates with an immuno-suppressive, stromal-rich phenotype, marked by fibroblast/pericyte genes (e.g., FN1, PDGFRB) and the ecto-enzyme ENTPD1 (CD39) (Fig. 3d). Finally, representative t-SNE plots from ovarian, breast, and kidney cancers visually confirm the cell-type-specific expression of IMPREG at the single-cell level (Fig. 3e).

### Regulon-level architecture reveals cell-type specific regulons converging on immune evasion

Next, we dissected the contribution of individual regulons to the IMPREG signature across cellular compartments and clinical cohorts. Single-cell analysis revealed that the IMPREG signature is not a monolithic program but a composite of distinct regulatory modules active in specific cell types converging on immune evasion (Fig. 3f). Specifically, the IMPREG signature in malignant cells is dominated by *SOX11*, *NCOA2*, *ZNF683*, and *ETV7*, whereas fibroblasts preferentially activate *ZFPM2* and *ZNF521*. Endothelial cells, in contrast, rely more on *ZNF521*, *NCOA2* and *NCOA3*, but notably lack *ZNF683 and ETV7*. This suggests that IMPREG captures a convergent phenotype of immune privilege achieved through diverse transcriptional regulons.

Clinically, this modularity results in context-dependent predictive power for individual regulons. Benchmarking across independent immunotherapy cohorts demonstrated that the composite IMPREG signature outperforms individual regulons and cell-type-specific sub-signatures (e.g., Neuronal-IMPREG), which lacked stable predictive performance across diverse cancer types (Supplementary Fig. 6a). Multivariable LASSO regression confirmed that no single regulon universally predicts resistance, highlighting the necessity of aggregation to capture context-dependent regulatory nodes (Supplementary Fig. 6b). Variance decomposition indicated that the ten regulons collectively explain nearly all IMPREG variance (median *R²* ≈ 0.99; Supplementary Dataset 1, Supplementary Fig. 6c). While *ZNF683* and *ETV7* act as stable biological anchors, the integration of context-adaptive regulons is essential to ensure the aggregated score captures emergent predictive utility unavailable from any single component.

### Distinct cell-state subtypes in IMPREG-high tumors

**Fig. 4:**
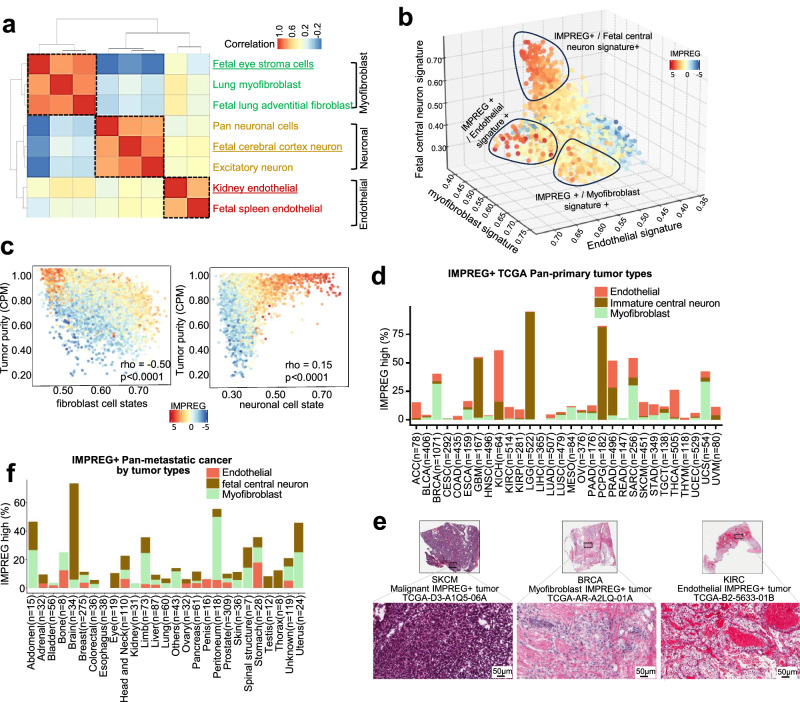
Cell-state signatures define distinct IMPREG-high tumor subtypes with unique TME characteristics. **a** Correlation matrix of the top IMPREG-driven cell-state signature scores most strongly associated with the IMPREG score, based on data from our scRNAseq compendium and TCGA pan-cancer dataset. **b** Three-dimensional (3D) scatter plot illustrating the distribution of TCGA tumors into three distinct IMPREG-high subtypes. Each dot represents a tumor sample, positioned in 3D space according to its cell-state signatures. **c** Scatter plots showing Spearman’s correlations between cell-state signatures and tumor purity across TCGA tumor samples (fibroblast: rho = −0.50; neuronal: rho = 0.15). **d** Stacked bar chart classifying IMPREG-high tumors into subtypes based on the predominant cell-state signature across distinct cancer types in TCGA. **e** Representative histology images of myofibroblast, central neuronal, and endothelial-dominant IMPREG-high TCGA tumors. Scale bars, 50 µm. **f** Stacked bar charts showing the proportional composition of IMPREG-high tumor subtypes across pan-metastatic cancer types, with each bar representing the percentage of IMPREG-high tumors attributed to each cell-state-defined subtype within a given cancer type. Source data are provided as a Source Data file.

To investigate cellular states associated with elevated IMPREG expression, we analyzed 1265 cell-state signatures in the pan-cancer scRNA-seq and TCGA bulk RNA-seq compendium, which identified three distinct tumor clusters with myofibroblast-like, neuronal-like, or endothelial-specific features (Fig. 4a). Using representative signatures from each cluster, we classified TCGA tumors into three IMPREG subgroups: myofibroblast, central neuronal, and endothelial-dominant (Fig. 4b, Supplementary Fig. 7). Correlation with tumor purity confirmed a malignant origin for the neuronal signatures and a stromal origin for the fibroblast signatures (Fig. 4c, Supplementary Fig. 8). The central neuronal subtype was prevalent in neuronal tumors and prostate cancer, while the myofibroblast subtype characterized stromal and solid tumors like breast and pancreatic cancer (Fig. 4d). Endothelial-dominant subtypes were observed mainly in kidney and thyroid cancers. IMPREG-high tumors collectively represent 23% of all tumors in the pan-cancer panel. Representative pathology images are shown in Fig. 4e.

### Dynamic remodeling during metastasis

We next examined how these IMPREG subtypes engage in metastasis by analyzing 1588 pan-metastatic tumors (Fig. 4f). This revealed a dynamic remodeling, with a pronounced enrichment of the neuronal IMPREG subtype in metastatic tumors (4.9%) compared to primary tumors (2.5%). To address potential confounding by tumor purity, we analyzed purity-stratified cohorts (Supplementary Fig. 8). We found that metastatic neuronal enrichment occurred independently of tumor purity; primary neuronal tumors actually exhibited higher purity than metastatic lesions (*p* < 0.0001; Supplementary Fig. 8c). Furthermore, while primary neuronal signatures were restricted to high-purity samples, metastatic tumors exhibited significant neuronal enrichment even in low- and medium-purity tiers, contrasting with the stromal dilution pattern of the myofibroblast subtype (Supplementary Fig. 8d, e). This enrichment suggests that the neuronal phenotype in metastasis reflects a specific, purity-independent biological selection for neuronal-like transcriptional programs during metastatic progression. Conversely, the endothelial IMPREG subtype was significantly depleted, decreasing from 7.4% in primary tumors to 3.4% in metastatic lesions (Supplementary Fig. 8f). To avoid confounding by tissues with intrinsic neuronal programs, analyses of neuronal subtype frequency in purity-stratified groups were performed after excluding tumors of neuronal origin (GBM, LGG, and PCPG).

### IMPREG signature spatially organizes an immunosuppressive TME

**Fig. 5:**
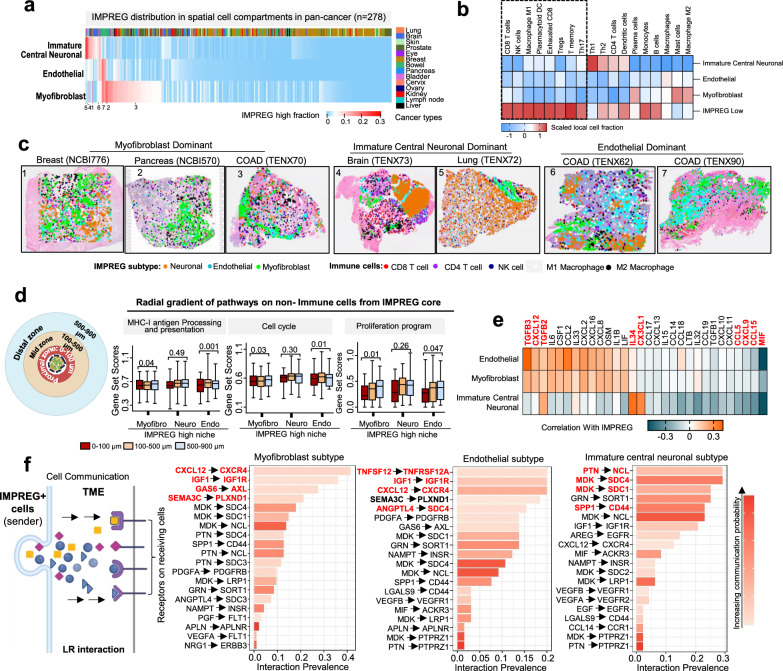
Spatial landscape of IMPREG subtypes reveals distinct TME across cancers. **a** Heatmap showing the percentage of IMPREG-high cells in cellular compartments defined by myofibroblast, immature central neuronal, and endothelial signatures across 278 pan-cancer tumors. Rows are individual tumors. **b** Spatial neighborhood analysis quantifying the local fractions of diverse immune cell populations to IMPREG-high and IMPREG-low cells. Heatmap values represent scaled location cell fractions. The local zone for the spatial neighborhood analysis is defined by an adaptive radius calculated for each slide as 2.5 times the median nearest-neighbor distance between adjacent spots. **c** Representative spatial transcriptomic maps showing IMPREG-high cell localizations in seven IMPREG-high tumors of three IMPREG subtypes. **d** Box and whisker plot showing radial gradients in pathway enrichment as a function of distance from IMPREG⁺ core regions in eligible slides (*n* = 97 tumor spatial slides). For each pathway, spot-level enrichment was quantified across three radial distance bands—0–100 µm, 100–500 µm, and 500–900 µm from the nearest IMPREG⁺ cluster. The box represents the interquartile range, whiskers indicate the minimum and maximum values, and the central line denotes the median enrichment score. *P*-values were calculated using paired one-tailed *t*-tests. **e** Spatial correlation heatmap showing subtype-dependent associations between IMPREG expression and chemokine/cytokine genes, with correlations calculated using Spearman’s rank correlation. **f** Heatmap summarizing CellChat-inferred ligand–receptor signaling from IMPREG-high subtypes (senders) to non-immune tumor cells (receivers) across the three IMPREG subtypes. Communication probabilities were computed using the triMean method ( ≥ 10 spots per group per slide). Significance was assessed by CellChat’s default permutation-based test, with per-slide *p*-values aggregated using Fisher’s combined probability and Benjamini–Hochberg FDR correction. Bars represent interaction prevalence (fraction of eligible slides with significant interaction); color intensity reflects mean communication probability. The schematic in panel (**f**) was created in BioRender (Lawal, B. (2026) https://BioRender.com/cius7x3). Source data are provided as a Source Data file.

To investigate the spatial architecture of the IMPREG signature, we analyzed 278 spatially profiled tumors from the HEST-1k compendium, a curated resource of pan-cancer spatial transcriptomics^53^. High IMPREG expression was concentrated in three cellular compartments, defining myofibroblast, immature central neuronal, and endothelial dominant subtypes across cancers (Fig. 5a). Spatial neighborhood analysis confirmed that IMPREG-high regions across all three subtypes are characterized by a profound exclusion of effector immune cells, including cytotoxic CD8 + T cells, M1 macrophages, and NK cells, as well as Tregs and exhausted T cells indicating lack of pre-existing immunity (Fig. 5b). However, the spatial immune exclusion patterns varied by IMPREG subtype: the Immature central neuronal cell type exhibited enrichment of CD4 T cells, whereas the Myofibroblast subtype showed strong enrichment of M2 macrophage and mast cells, both of which are associated with wound healing. Representative spatial maps visually confirmed this subtype-specific organization, showing dense myofibroblast-driven foci in pancreatic and breast tumors, immature neuronal-like clusters in brain and lung tumors, and endothelial-rich regions in kidney tumors (Fig. 5c).

Next, we performed radial gradient analysis quantifying pathway enrichment in concentric bands extending outward from IMPREG-high cores (Fig. 5d). This analysis revealed that IMPREG organizes the tumor microenvironment into distinct functional zones. The proximal core (0–100 µm) across all three subtypes exhibited the lowest levels of Antigen Presentation Proficiency, establishing a localized hub of immune suppression. In contrast, cell cycle and proliferation programs were enriched in distant zones, suggesting active tumor growth occurs peripherally to the IMPREG-positive niches.

### Distinct cytokine landscapes define the immune-privileged architecture of IMPREG nests

We next investigated the cytokine networks sustaining this immune-privileged architecture (Fig. 5e). Three cytokines demonstrated strong positive correlation with IMPREG expression. TGFB3, which drives fibroblast-to-myofibroblast transition and directly suppresses T-cell cytolytic genes, including perforin, granzymes, and IFN-γ^54^. CXCL12 functions as a “stromal trap” that arrests T-cells within the stroma via CXCR4 engagement, preventing their migration toward IMPREG nests^55^. IL-34 and CX3CL1 are neuron-derived factors that maintain microglia in anti-inflammatory states. IL-34 signals through CSF1R to polarize microglia toward an anti-inflammatory reparative phenotype^56^. CX3CL1 signals through CX3CR1 to suppress microglial production of pro-inflammatory mediators, including TNF-α, IL-1β, and nitric oxide^57^.

Conversely, three key cytokines were downregulated within IMPREG-high cores, reflecting suppression of immune activation pathways. CCL15, which normally recruits monocytes to tumor sites, was markedly reduced. CXCL9, a critical CXCR3 ligand required for CD8 + T-cell trafficking and anti-PD-1 efficacy^58^, showed a strong inverse correlation with IMPREG. MIF (macrophage migration inhibitory factor), which promotes immune cell recruitment and antigen-presenting cell function^59^, was similarly suppressed.

### Subtype-specific ligand-receptor interactions underpin IMPREG-mediated immune exclusion

CellChat analysis of ligand-receptor interactions revealed distinct immunosuppressive mechanisms across IMPREG subtypes (Fig. 5f). In the myofibroblast subtype, CXCL12 → CXCR4 signaling from FAP + CAFs created a T cell exclusion barrier while stabilizing PD-L1 expression^55^. IGF1 → IGF1R supported metabolic reprogramming and M2 macrophage polarization^60^, while GAS6 → AXL mediated efferocytosis and NK cell inhibition^61^. SEMA3C → PLXND1 is a key axis promoting CAF activation and ECM remodeling^62^. The endothelial subtype was dominated by TNFSF12 → TNFRSF12A, which promotes angiogenesis and myeloid recruitment^63,64^. IGF1 → IGF1R on tumor endothelial cells regulated tip cell maintenance and sprouting angiogenesis while enhancing Treg/MDSC recruitment. CXCL12 → CXCR4 on tumor endothelial cells inhibits CD8 + T cell activation, while ANGPTL4 → SDC4 regulates vascular permeability, affecting immune cell extravasation^65^. The immature central neuronal subtype exhibited developmentally regulated interactions: PTN → NCL (Pleiotrophin-Nucleolin) axis, which has established roles in neurotrophic signaling and the promotion of an immunosuppressive niche^66,67^. MDK → SDC4/SDC1 drove M2 polarization and promoted CD8 + T cell dysfunction^68^, and SPP1 → CD44 functioned as a direct immune checkpoint suppressing CD8 + T cell activation^69^. Collectively, these spatial analyses demonstrate that IMPREG orchestrates a complex, subtype-specific signaling network that simultaneously creates physical barriers, establishes biochemical traps, and actively repels effector immune cells to maintain an immune-privileged tumor microenvironment.

### Validation using internal UPMC patient cohort: IMPREG-high TNBC tumors display T-cell desertion and myofibroblast-rich TME

**Fig. 6:**
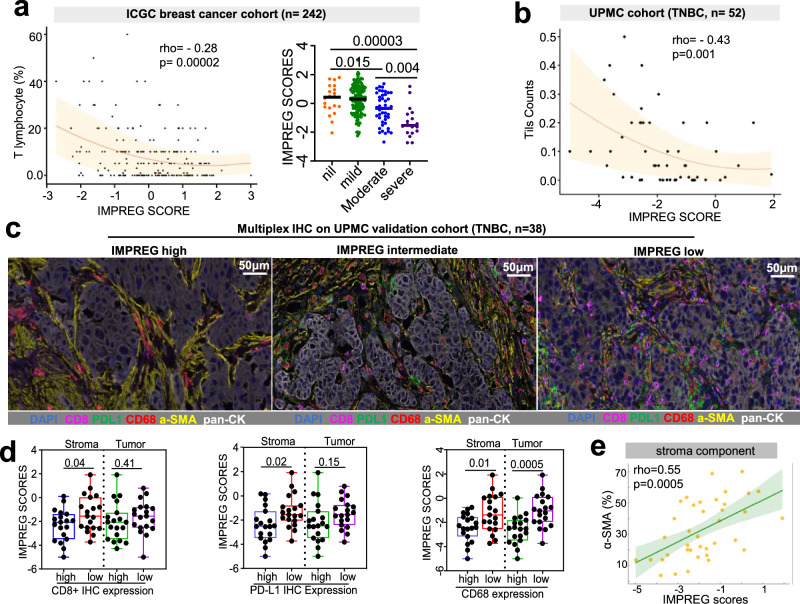
IMPREG-high breast tumors exhibit T cell desertion and myofibroblast-rich TME in ICGC and UPMC breast cancer cohorts. **a** Scatter plots show Spearman correlations between IMPREG and pathologically determined TIL percentage in the ICGC breast cancer cohort (*n* = 242 independent patient tumors; rho = −0.28). Shaded bands represent smoothed residual envelopes around the fitted regression line. Adjacent bar plots depict IMPREG scores across varying pathological immune phenotypes of the TME. The middle line represents the median value. *P*-values were calculated using unpaired two-tailed *t*-tests. **b** Scatter plots showing Spearman correlations between IMPREG scores and TIL percentages in our UPMC TNBC cohort (*n* = 52 independent patient tumors, rho = −0.43). Shaded bands represent smoothed residual envelopes around the fitted regression line. **c** Representative multiplex IHC images from the UPMC TNBC cohort, displaying markers CD8, PD-L1, CD68, αSMA, and pan-CK in both tumor and stromal regions across IMPREG high, intermediate, and low groups. Color-coded overlays identify specific markers within each region. **d** Box-and-whisker plots comparing IMPREG expression levels in tumor and stromal compartments of the UPMC cohort (*n* = 38 independent patient tumors), stratified by immune markers: CD8+, PD-L1+, and CD68+. Boxes represent the interquartile range, whiskers indicate minimum and maximum values, and the central line denotes the median IMPREG score. *P*-values were calculated using unpaired two-tailed *t*-tests. **e** Scatter plot showing two-sided Spearman correlation of IMPREG scores with αSMA (myofibroblast) staining in the stromal region (*n* = 38, rho = 0.55). Shaded band represents 95% confidence interval. Source data are provided as a Source Data file.

To validate IMPREG’s role in shaping the tumor microenvironment (TME), we analyzed its association with immune infiltration. In a large breast cancer cohort from International Cancer Genome Consortium (ICGC)^70^, we observed a significant inverse relationship between IMPREG scores and T-cell infiltration (Fig. 6a). We confirmed this finding experimentally in our internal UPMC cohort of TNBC patients, where IMPREG scores negatively correlated with TILs (rho = -0.43, *p* = 0.001; Fig. 6b), and other immune cell populations (Supplementary Fig. 9a). Multiplex IHC staining on 38 TNBC tumors further revealed that high IMPREG expression was associated with lower densities of CD8+, PD-L1+, and CD68+ immune cells in both tumor and stromal regions (Fig. 6c, d, Supplementary Fig. 9). Aligning with our proposed IMPREG subtyping, IMPREG-high TNBC tumors were predominantly characterized by a myofibroblast-rich TME. This was demonstrated by a strong correlation between IMPREG scores and the myofibroblast marker αSMA in the stroma (rho = 0.55, *p* = 0.0005; Fig. 6e). These experimental results confirm that IMPREG is a key driver of T-cell desertion and an immuno-suppressive, myofibroblast-rich TME in TNBC.

### IMPREG shows consistent predictive value across a wide array of validation clinical trial datasets involving various immunotherapy modalities

**Fig. 7:**
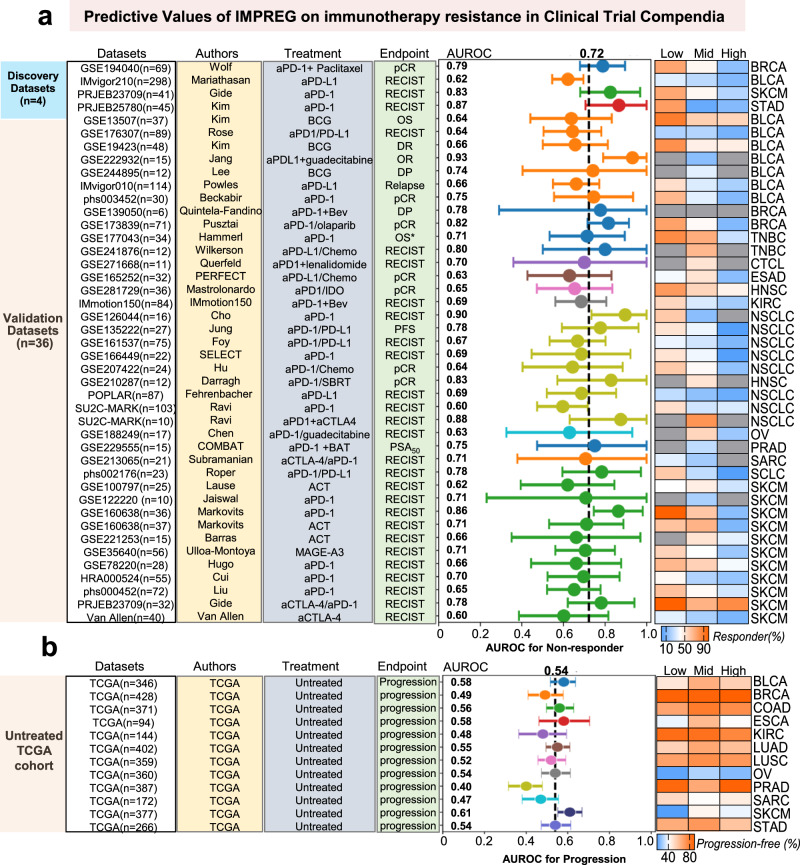
IMPREG as a predictive biomarker for immunotherapy outcomes across diverse modalities. **a** Forest plot summarizing the performance of the IMPREG score for distinguishing non-responders (resistant) from responders across multiple immunotherapy cohorts. **b** The predictive values of IMPREG on tumor progression in the TCGA untreated patient cohorts. Each row corresponds to an individual study cohort, with the dataset ID and sample size (n), first author, treatment regimen, and clinical response endpoint indicated on the left. Cohorts are grouped into discovery (top) and validation (bottom) collections. The dots (center of the error bars) indicate the AUROC for predicting resistance using IMPREG, and horizontal whiskers denote the 95% confidence interval (error bars). The vertical dashed line represents the mean AUROC. The heatmap on the far right shows the observed responder fraction (%) after stratifying samples into Low, Mid, and High IMPREG groups within each cohort. The RR was defined as the percentage of responder patients within each group. IMPREG groups were defined using a median ± median absolute deviation (MAD) framework: IMPREG-low (< Median − MAD), IMPREG-intermediate (≥ Median − MAD and ≤ Median + MAD), and IMPREG-high (> Median + MAD). A default constant of 1 was applied for MAD calculation. The gray color represents groups with less than 5 samples and were exempted from the RR calculation. RECIST, Response Evaluation Criteria in Solid Tumors; ORR, overall response rate; pCR, pathological complete response; OS, overall survival; PFS, progression-free survival; PSA_50_, ≥50% decline in prostate-specific antigen. Source data are provided as a Source Data file.

To assess the efficacy of the IMPREG score as a predictive biomarker for response to ICB therapy, we aggregated 36 additional independent clinical trial datasets—predominantly anti-PD-1/PD-L1 treated cohorts, supplemented by other immunotherapy modalities like Adoptive Cell Transfer (ACT) and BCG—to evaluate the IMPREG score as a predictive biomarker for immunotherapy response (Fig. 7, Supplementary Dataset 4 and Supplementary Fig. 10). Across a diverse range of cancer types, IMPREG demonstrated an average AUROC of 0.72 for predicting resistance. In addition to anti-PD-1/PD-L1, IMPREG consistently retained its predictive accuracy across various immunotherapy modalities such as ACT, anti-CTLA4, and anti-MAGE-A3 antibody. In the case of adoptive T cell therapies for SKCM, AUROC values ranged from 0.62 to 0.71. In contrast, in TCGA untreated patients, IMPREG did not predict tumor progression or patient death (Fig. 7b, Supplementary Fig. 11). This suggests IMPREG as a specific indicator of immunotherapy resistance. Across the immunotherapy datasets, the response rates were highest in the IMPREG-low group (median 60.00%), followed by the IMPREG-intermediate group (median 38.9%), with the lowest rates observed in the IMPREG-high group (median 14.8%) (Fig. 7a). IMPREG scores consistently aligned with immune-desert and PD-L1-low phenotypes across multiple cohorts (Supplementary Fig. 12).

Of note, IMPREG showed moderate efficacy in predicting anti-PD-L1 response to bladder cancer in the IMvigor210 trial (AUROC = 0.62). Interaction analysis revealed that prior immune modulation significantly enhanced IMPREG’s predictive performance (Supplementary Fig. 13a, b). Specifically, predictive accuracy improved with prior platinum exposure (Supplementary Fig. 13a) and reached an AUROC of 0.84 in BCG-exposed patients (Supplementary Fig. 13b). In BCG-treated cohorts—48 patients from the Kim trial^71^ and 12 from Lee et al.^72^—IMPREG predicted BCG resistance with an AUROC of 0.66 and 0.74, respectively (Supplementary Fig. 13c).

### IMPREG is orthogonal to known immune signatures and outperforms established immunotherapy biomarkers

**Fig. 8:**
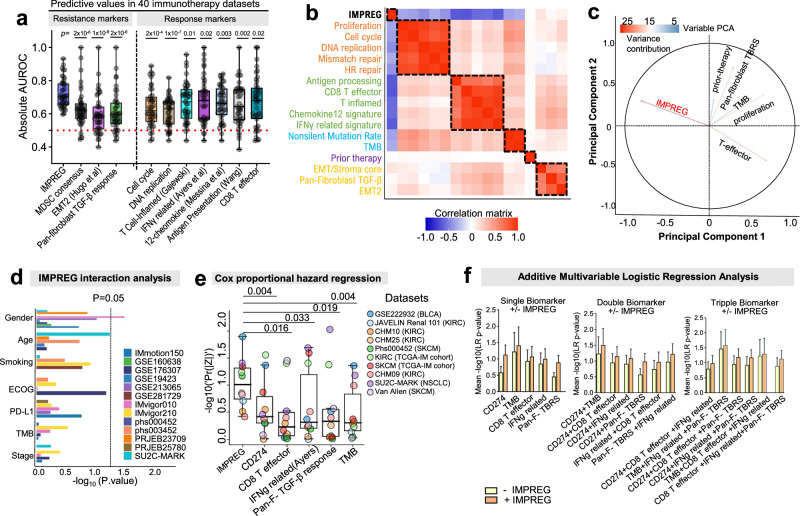
Evaluation of IMPREG predictive accuracy, orthogonality, and additive value in immunotherapy cohorts. **a** Boxplots comparing the absolute AUROC of IMPREG versus established immunotherapy biomarkers across 43 treatment arms from 40 immunotherapy datasets. Each point represents one treatment arm’s AUROC (*n* = 43). Boxes: interquartile range; center line: median; whiskers: minimum to maximum values. Statistical significance of each biomarker’s performance relative to IMPREG was assessed using a paired two-sided *t*-test. The *p*-values for comparisons with IMPREG are shown above the corresponding boxplots. **b** Correlation matrix showing pairwise Spearman correlations between IMPREG and 16 tumor-intrinsic and immune-related features. **c** Principal component analysis (PCA) variable biplot illustrating feature projections onto the first two principal components. IMPREG loads orthogonally along PC1 and is spatially separated from other biomarkers, indicating that it captures a distinct and independent axis of variance. **d** Bar plot summarizing interaction testing between IMPREG and major clinical covariates (age, sex, ECOG status, PD-L1 expression, TMB, smoking status, and disease stage) across immunotherapy cohorts (*n* = 13 datasets) with adequate sample size for covariate modeling. Bars represent −log_10_(*P*-values) from two-sided likelihood ratio chi-squared tests comparing main effects versus interaction models (see “Methods”). The dashed line indicates *p* = 0.05. **e** Boxplots showing the distribution of −log_10_(*P*-values) for individual predictors derived from multivariable Coxph regression models of the disease progression free survival across immunotherapy cohorts with available TMB data. Each point denotes a dataset-specific effect size (*n* = 10 cohorts). Boxes represent IQR (25th–75th percentile), center line denotes median, whiskers extend to 1.5 × IQR, points beyond whiskers represent outliers. Statistical comparisons were performed using one-tailed Wilcoxon paired signed-rank tests. **f** Multivariable modeling analysis illustrating the added predictive contribution of IMPREG in composite models. Bars represent the mean −log_10_(likelihood-ratio [LR] *p*-values) across 13 independent immunotherapy datasets for models containing single biomarkers, double-biomarker combinations, or triple-biomarker combinations, shown with and without IMPREG. Error bars indicate SEM. Source data are provided as a Source Data file.

To evaluate IMPREG’s predictive value against established biomarkers, we compared their absolute AUROC scores across the expanded compendia of 40 immunotherapy datasets for 14 cancer types used in our study. IMPREG consistently outperformed both resistance-associated biomarkers such as MDSC, epithelial mesenchymal transition (EMT), pan-fibroblast TGF-β response, and response markers such as antigen presentation proficiency, cell cycle, interferon γ, and T effector signatures (Fig. 8a). Benchmarking metrics including sensitivity, specificity, and Area Under Precision-Recall Curve (AUPRC) confirmed IMPREG’s robustness (Supplementary Fig. 14). To investigate whether IMPREG represents a redundant variable of five canonical latent tumor programs underlying response to immunotherapy recently reported by Usset et al (Nat Genet. 2024)^73^, a correlation analysis was performed across 16 tumor-intrinsic and immune features included in that study. While other features grouped into five distinct correlation clusters (proliferation, immune activation, EMT/ TGF-β response, prior therapy, and TMB), IMPREG appeared as a standalone node in the correlation heatmap, suggesting that it is not reducible to known canonical latent tumor programs (Fig. 8b). To evaluate IMPREG’s role within the tumor immune landscape, we performed principal component analysis (PCA) alongside the five immune-related programs. All these variables were grouped into six principal components, and IMPREG emerged as the primary contributor to the first principal component (PC1), accounting for 29% of the variance (Supplementary Fig. 15). Notably, its vector opposes those of T-cell effector, TMB, and proliferation—factors with minimal influence on PC1. This opposing orientation and spatial separation underscore IMPREG’s role as an orthogonal axis (Fig. 8c).

### IMPREG provides independent, non-redundant predictive power

Interaction analysis across immunotherapy cohorts confirmed that IMPREG’s predictive value is robust and independent of clinical context (Fig. 8d). No significant interactions were found between IMPREG and key clinical variables—including age, gender, smoking status, ECOG performance, PD-L1 expression, TMB, or clinical stage—indicating stable predictive accuracy across diverse patient demographics. Stratified AUROC analyses further validated this consistency across subgroups (Supplementary Datasets 2, 3). Moreover, within harmonized datasets containing matched TMB data, multivariable Cox proportional hazard regression identified IMPREG as the strongest predictor of response, significantly outperforming TMB, CD274, interferon-γ, CD8 T effector, and pan-fibroblast TGF-β signatures (Fig. 8e). Finally, additive multivariable models demonstrated that incorporating IMPREG consistently improved model fit for all single- and multi-biomarker combinations (Fig. 8f), confirming that IMPREG provides independent, non-redundant predictive power beyond established biomarkers.

### IMPREG confers sensitivity to selected targeted agents

**Fig. 9:**
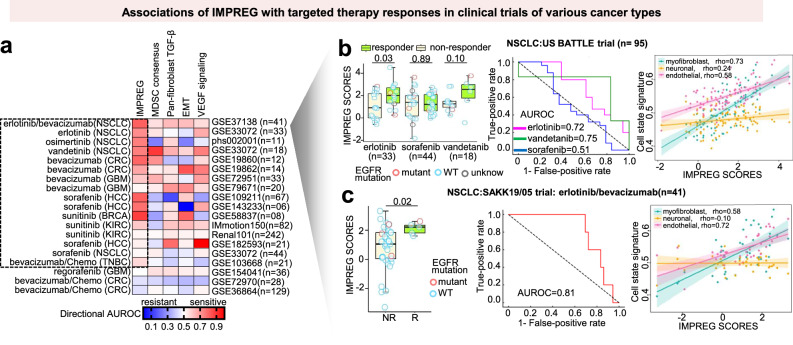
IMPREG confers a sensitive response to selected targeted agents. **a** Heatmap showing the predictive performance of IMPREG and established immunotherapy resistance biomarkers across 17 clinical trials of targeted therapies spanning seven cancer types. **b** In the BATTLE trial, IMPREG predicts sensitivity to erlotinib and vandetanib. Box-and-whisker plots compare IMPREG scores between responders (R) and non-responders (NR) for erlotinib (*n* = 33 subjects), sorafenib (*n* = 44 subjects), and vandetanib (*n* = 18 subjects). Boxes: IQR (25th–75th percentile); center line: median; whiskers: 1.5 × IQR; points beyond whiskers: outliers. *P*-values were calculated using two-sided Mann-Whitney *U*-tests. ROC curves illustrate IMPREG’s predictive performance across treatment arms. Scatter plots show Spearman correlations between IMPREG scores and cell-state signatures: myofibroblast (rho = 0.73), neuronal (rho = 0.24), and endothelial (rho = 0.58); shaded bands represent 95% confidence intervals. **c** In the SAKK 19/05 trial, IMPREG predicts sensitivity to erlotinib plus bevacizumab (*n* = 41). Box-and-whisker plots compare IMPREG scores between responders and non-responders. Boxes: IQR (25th–75th percentile); center line: median; whiskers: 1.5 × IQR; points beyond whiskers: outliers. *P*-values were calculated using two-sided Mann-Whitney *U*-tests. ROC analysis: AUROC = 0.81. Scatter plots show Spearman correlations between IMPREG scores and myofibroblast (rho = 0.58), neuronal (rho = −0.10), and endothelial (rho = 0.72) cell-state signatures; shaded bands represent 95% confidence intervals. Source data are provided as a Source Data file.

To evaluate whether IMPREG could also inform targeted therapy decisions, we analyzed 17 clinical trial datasets spanning seven cancer types. Remarkably, in contrast to its role in predicting resistance to ICB, only IMPREG—and not other established immunotherapy biomarkers like TGF-β, EMT, or MDSC signatures—predicted increased sensitivity to EGFR inhibitors and anti-angiogenic agents (Fig. 9a). This specific predictive value was evident in non-small cell lung cancer (NSCLC) patients treated with EGFR inhibitors (erlotinib, osimertinib) regardless of EGFR mutation status, as seen in the BATTLE trial (AUROC = 0.72) and the Swiss SAKK 19/05 trial (AUROC = 0.81) (Fig. 9b, c). Mechanistic analysis revealed that this sensitivity correlates with the myofibroblast and endothelial components of the IMPREG signature, suggesting that IMPREG identifies a distinct tumor subset where stromal and vascular remodeling may create vulnerabilities to EGFR inhibition. Furthermore, IMPREG successfully predicted sensitivity to anti-angiogenic therapies (bevacizumab, sunitinib, sorafenib) in colorectal, breast, kidney, and liver cancers, surpassing the VEGF signaling signature. This finding highlights IMPREG as a unique dual-utility biomarker capable of stratifying patients for both immunotherapy and targeted treatments.

## Discussion

Our study identifies the IMPREG signature as a central transcriptional mechanism that tumors co-opt to establish immune-privileged microenvironments, driving T cell exclusion and resistance to ICB therapies across multiple cancer types. This signature mirrors transcriptional programs found in immune-privileged tissues, such as the retina, central nervous system, and gonads, which naturally evade systemic immune responses to protect specialized functions. By co-opting these immune-privileged pathways, tumors may create a hostile environment for anti-tumor immune cells, leveraging multifaceted mechanisms to suppress T cell infiltration, downregulate antigen presentation pathways, and reduce pro-inflammatory signaling. These findings underscore the critical clinical utility of IMPREG as a versatile biomarker for predicting immunotherapy resistance across diverse cancer types, offering immediate implications for refining patient stratification and guiding the selection of targeted combination therapies.

### Tumors exploit transcriptional programs underlying immune privilege

The discovery that IMPREG mirrors transcriptional programs intrinsic to immune-privileged tissues such as the retina, central nervous system, and gonads is noteworthy. These organs naturally evade systemic immune responses to protect their specialized functions by preventing cytotoxic immune infiltration while maintaining homeostasis through macrophages and other immunosuppressive cells. While previous research has attributed immune privilege to structural barriers and immune mechanisms, our findings underscore the critical involvement of a set of specific transcriptional regulators, an underexplored dimension of immune privilege that warrants further investigation. The IMPREG signature may orchestrate immune evasion through multifaceted mechanisms that reshape the TME into an immune-depleted and immunotherapy-resistant state. It suppresses antigen presentation, dictates an immunosuppressive cytokine contexture, and promotes stromal remodeling via myofibroblasts and endothelial barriers, thereby impairing T cell infiltration. Furthermore, IMPREG is associated with diminished B and T cell receptor diversity, impairing adaptive immunity and enhancing immunosuppressive pathways such as TGF-β signaling. These multifaceted changes may synergistically disrupt innate and adaptive immune responses, fostering immune escape and therapeutic resistance.

### Orthogonality to canonical immune signatures

A critical finding of our study is the orthogonality of IMPREG to established immune signatures. Recent large-scale analyses have identified five canonical latent tumor programs, including proliferation, TMB, and T-effector signals, that underlie response to immunotherapy^73^. Our analysis reveals that IMPREG does not merely recapitulate these known factors; rather, it emerges as a primary, independent contributor to TME variation, projecting as a unique axis distinct from T-cell inflammation or mutational burden. This distinction highlights the uniqueness of IMPREG: it captures a “cold” tumor state driven by active immune-privilege co-option rather than a simple lack of immunogenicity. Unlike signatures derived from post-hoc outcome analysis (T-cell-inflamed GEP, IFN-γ signatures), IMPREG is grounded on the transcriptional mechanism of immune-privilege, providing mechanistic interpretability. Furthermore, IMPREG integrates regulon signatures across malignant, fibroblast, and endothelial cells, capturing convergent immune privilege regardless of cellular origin— this integrative design underpins IMPREG’s consistent performance across diverse tumor types. Consequently, IMPREG provides non-redundant predictive power, consistently outperforming standard biomarkers like TMB and T-cell-inflamed signatures in multivariate models. This suggests that IMPREG identifies a distinct subset of non-responders who might be misclassified by conventional markers, addressing a critical gap in current precision oncology frameworks.

### Distinct cell types and states mediate tumor-specific immune evasion

Our study reveals that tumors employ distinct cellular compartments, including malignant cells, fibroblasts, or endothelial cells, to execute IMPREG-mediated immune evasion. This adaptability underscores the versatility of tumors in tailoring their immune-privileged state to the TME’s composition and context^74^.

1. **Malignant cell dominance and metastatic remodeling:** In cancers such as glioblastomas, neuroendocrine, and prostate cancers, malignant cells exhibit high IMPREG expression, mimicking the transcriptional states of immature central neurons. Furthermore, our analysis of pan-metastatic tumors revealed a pronounced enrichment of neuronal IMPREG subtypes in metastatic epithelial tumors, suggesting a possible selection for neuronal-like transcriptional programs during metastatic progression. This highlights an increased reliance on tumor neuronal states to maintain immune evasion post-metastasis. While previous studies have linked neuronal signaling to immune-privileged niches^75,76^, our findings broaden this framework, implicating neuronal IMPREG programs as a potential driver of immune escape in metastatic lesions.
2. **Fibroblast- or endothelial-mediated evasion:** IMPREG’s interactions with fibroblasts and endothelial cells appear to be more prevalent in primary tumors. In cancers like TNBC and pancreatic adenocarcinoma, fibroblasts with myofibroblast-like features dominate the IMPREG-high signature. These myofibroblasts may remodel the extracellular matrix (ECM) and secrete immunosuppressive factors such as TGF-β, further suppressing T cell infiltration^77,78^. Additionally, in kidney and liver-biliary tumors, endothelial cells overexpressing IMPREG adopt programs resembling kidney endothelial cells, creating vascular barriers that exclude immune cells^79^. This compartmentalization suggests that therapeutic strategies must be tailored to the specific cellular source of IMPREG expression within a given tumor type.

### Clinical applications: stratification for immunotherapy and targeted agents

Our study demonstrates that IMPREG serves as a robust, dual-utility biomarker capable of guiding decision-making for both immunotherapy and targeted treatments. First, as a predictor of resistance, IMPREG identifies an immune-deserted phenotype characterized by diminished T cell infiltration and poor response to ICB therapies across multiple cancer types. The strong predictive value in melanoma, breast cancer, and NSCLC suggests that IMPREG could be integrated into screening workflows to spare patients from futile ICB monotherapy and its associated toxicities. Crucially, beyond predicting resistance, IMPREG identifies tumors with heightened sensitivity to targeted therapies. In NSCLC cohorts, IMPREG-high patients showed notable responsiveness to EGFR inhibitors (e.g., erlotinib, osimertinib), independent of EGFR mutation status. EGFR signaling is known to drive TME remodeling and immune suppression through fibroblast and endothelial cell activation^80,81^, which aligns with IMPREG’s association with myofibroblast and endothelial signatures in these datasets. Similarly, IMPREG predicted sensitivity to anti-angiogenic agents in renal and hepatocellular carcinomas. These findings support a therapeutic redirection strategy for IMPREG-high patients.

To guide future clinical translation, we envision a potential IMPREG-guided decision framework that warrants future validation. IMPREG scoring could be incorporated into molecular workups alongside PD-L1 and TMB to provide complementary prediction. Based on our findings, IMPREG-high NSCLC patients without actionable drivers may be candidates for exploring EGFR inhibitors or EGFR plus anti-angiogenic combinations (e.g., erlotinib/bevacizumab), particularly the myofibroblast-dominant subtype. In endothelial-dominant cases of IMPREG-high RCC, HCC, and CRC—anti-VEGF/VEGFR agents warrant investigation for their potential to disrupt vascular barriers facilitating immune exclusion. Notably, sequential or concurrent combination of these targeted agents with ICB warrants exploration, as TME normalization may restore immunotherapy responsiveness. While these applications require validation in prospective clinical trials, our data suggest IMPREG may serve as a complementary biomarker to identify patients unlikely to benefit from ICB monotherapy who could be prioritized for targeted agents addressing stromal and vascular components of immune privilege.

### Data limitations and future directions

While IMPREG demonstrates broad generalizability, several limitations warrant consideration. First, data heterogeneity from different institutions and sequencing platforms introduces batch effects that may affect IMPREG scores near threshold cutoffs; future implementations should establish platform-specific calibration standards. Second, cohort variability in prior treatments confounds performance; for instance, BCG or platinum chemotherapy in BLCA cohorts may reshape the immune microenvironment and obscure IMPREG signals, potentially biasing predictive estimates. Prospective studies should stratify by treatment history or use propensity score matching. Third, the retrospective design limits causal inference; unmeasured confounders may influence observed associations. Randomized biomarker-stratified trials are essential to establish clinical utility and optimal thresholds. Fourth, single-cell and spatial analyses from limited samples may underrepresent IMPREG cellular diversity; larger atlases across diverse demographics are needed to refine cell-type-specific targeting strategies.

To fully realize the clinical potential of IMPREG, future research should integrate multi-omics and spatial transcriptomics. Our spatial analysis revealed that IMPREG organizes the TME into functional zones, creating “stromal traps” via TGF-β and CXCL12 that physically exclude T cells. High-resolution spatial mapping in future trials could help precisely delineate these zones and identify specific druggable targets within the IMPREG niche. Furthermore, expanding analysis to additional modalities, such as bispecific antibodies or CAR-T therapies, could broaden IMPREG’s applicability. Ultimately, prospective trials using IMPREG as a stratification tool are essential to establish its utility in personalized oncology, guiding the selection of combination therapies, such as ICB plus EGFR or VEGF inhibitors, to overcome immune privilege and improve patient outcomes.

In conclusion, our study identifies the IMPREG signature as a pivotal, orthogonal transcriptional mechanism by which tumors establish immune-privileged microenvironments. Distinct from canonical immune markers, IMPREG mirrors physiological immune-privilege programs and orchestrates T cell exclusion via cell-type-specific mechanisms in malignant, fibroblast, and endothelial compartments. Importantly, IMPREG offers dual clinical utility: predicting resistance to ICB while simultaneously identifying tumors responsive to targeted EGFR and anti-angiogenic therapies. These findings provide a strong rationale for integrating IMPREG into precision oncology workflows to optimize patient selection and guide the development of combination strategies that dismantle tumor immune privilege.

## Methods

### Ethics statement

This study complies with all relevant ethical regulations. Deidentified archival FFPE tissue slides were obtained from the Pitt Biospecimen Core, University of Pittsburgh, under Institutional Review Board Honest Broker Approval Number HB015. No direct patient contact or recruitment was involved. Informed consent was not required, as only deidentified archival specimens were provided through the certified honest broker protocol, and the study did not constitute human subjects research under 45 CFR 46. This study did not involve animal experiments; therefore, institutional limits on maximal tumor size/burden are not applicable. The cohort comprises 67 female patients with triple-negative breast cancer (age range and clinical characteristics in Supplementary Dataset 5). Sex and gender were not considered as study design variables, as TNBC predominantly affects female patients, and no male cases were present in this archival cohort.

### Data retrieval and preprocessing

The data processing workflow and datasets used in this study are described in Supplementary Fig. 1. A compendium of single-cell RNA sequencing datasets from 31 normal tissues, comprising 80 single-cell types, was obtained from the Human Protein Atlas portal (HPA, www.proteinatlas.org)^82^. scRNA seq data from 40 tumor datasets across 13 cancer types (12 solid tumor types used in downstream analyses after exclusion of hematological malignancies) were sourced from the Curated Cancer Cell Atlas (3CA) database^52^. The Bulk RNA-seq data for the Cancer Genome Atlas (TCGA) pan-cancer were retrieved from the UCSC Xena browser (https://xenabrowser.net)^83^, while bulk RNA-seq data for 37 healthy tissue types of the Genotype-Tissue Expression (GTEx) project were obtained from the HPA portal. Bulk RNA-seq data for the International Cancer Genome Consortium (ICGC) breast cancer cohort, comprising 242 samples, were obtained from the ICGC data portal (https://dcc.icgc.org/)^70^. The bulk RNA-seq data for the metastatic tumor (*n* = 1588) were obtained under controlled access in dbGAP (phs000673.v4.p1). In addition, bulk RNA-seq and clinical data from 40 clinical trial datasets for immunotherapy and 17 datasets for targeted therapy, spanning various cancer types, were sourced from publicly available repositories, including the Gene Expression Omnibus (GEO; http://www.ncbi.nlm.nih.gov/geo), the European Genome-Phenome Archive (https://ega-archive.org), or the database of Genotypes and Phenotypes (https://dbgap.ncbi.nlm.nih.gov/). From the 40 immunotherapy datasets, together with three additional cohorts reporting tumor mutational burden (TMB), we assembled harmonized TMB-annotated datasets using author-reported TMB values from the original publications. Detailed information of the clinical datasets, including the available genomic datasets accession, treatment arms, clinical endpoints, and source publications are provided in Supplementary Dataset 4.

### Clinical samples

Formalin-fixed paraffin-embedded (FFPE) slides of Triple-negative breast cancer (TNBC) patients (*n* = 67, Gender = female) were obtained from patients diagnosed with TNBC from September 1991 to May 2016 at the UPMC Hillman Cancer Center. Clinical characteristics of the included patients are summarized in Supplementary Dataset 5.

### Histopathological evaluation

Tissue specimens were evaluated by a pathologist, who was blinded to the genomic data to eliminate potential bias. Tumor-infiltrating lymphocytes (TILs) were assessed and scored, including tumor TILs, stromal TILs, and total TILs, using standard hematoxylin and eosin (H&E)-stained slides of formalin-fixed, paraffin-embedded (FFPE) surgical specimens. Scoring followed standardized criteria to ensure consistent grading of immune infiltration and histological characteristics across specimens.

### Multiplex immunohistochemistry (IHC)

Multiplex IHC and multispectral imaging were carried out at the Translational Pathology Imaging Lab core at UPMC Hillman Cancer Center. Individual antibodies were optimized as single stains according to supplier recommendations using appropriate control tissues. Multiplex panel optimization was carried out according to Akoya Bioscience’s “Opal Assay Development Guide”. Automated multiplex staining of tissues was performed on the Leica Bond RX. All staining reagents were provided in the Opal 6-Plex Detection Kit (Akoya Bioscience, cat# NEL871001KT), which was used according to the manufacturer’s instructions for sequential staining of each antibody in the panel. Primary antibody information is detailed in Supplementary Dataset 6. Akoya Bioscience’s PhenoimagerHT platform and InForm® analysis software (version 2.8) were used for 20x whole slide scanning and spectral unmixing of 20x regions of interest.

### Library preparation and bulk RNA-sequencing

High-quality RNA was isolated from formalin-fixed, paraffin-embedded (FFPE) tumor samples using guanidinium thiocyanate-phenol-chloroform extraction (TRIzol; Thermo Fisher Scientific). mRNA sequencing (mRNA-seq) was performed by Novogene Co., Ltd., following a rigorous protocol and quality control to ensure data quality. For library construction, mRNA was purified from total RNA using poly-T oligo-attached magnetic beads. After fragmentation, first-strand cDNA synthesis was performed using random hexamer primers, followed by second-strand cDNA synthesis. Libraries were constructed through successive steps of end repair, A-tailing, adapter ligation, size selection, amplification, and purification. The libraries were quantified using Qubit and real-time PCR and checked for size distribution on a Bioanalyzer. Quantified libraries were pooled based on concentration and target data requirements, then sequenced on the Illumina NovaSeq platform, generating 150 bp paired-end reads.

### Selection of discovery datasets

To construct a robust discovery framework, we employed a **consensus discovery approach** to identify immune-privileging regulons. We selected four discovery datasets—IMvigor210 (BLCA; anti-PD-L1), ISPY2 (BRCA; anti-PD-1 + paclitaxel), Kim (STAD; anti-PD-1), and Gide (SKCM; anti-PD-1)—based on: (i) available pretreatment RNA-seq data, (ii) high-quality clinical and response data, (iii) representation of distinct immunotherapy contexts across cancer types, and (iv) being the largest publicly available datasets with these characteristics in their respective indications at the time we initiated this study. To mitigate concerns about sample-size imbalance, each dataset was analyzed independently (Supplementary Dataset 7), so that regulon selection was not driven by a large-sized dataset (Supplementary Dataset 8).

### Inference of transcriptional factors activities using Dorothea and selection of IMPREG regulon signature

To systematically infer transcription factor (TF) activity, we acquired a list of 1344 human TF regulons (TF-target genes) from the DoRothEA v0.11 R package^43^. DoRothEA is a highly curated resource for regulon target genes that integrates diverse evidence, including peaks, binding site motifs, and gene expression interactions—thereby minimizing noise and maximizing confidence in the inferred regulatory network. The DoRothEA package (v0.11) implemented VIPER to estimate regulon activities from gene expression data, as described previously^43^. VIPER surpasses traditional transcriptional ranking methods by explicitly incorporating the signed mode of regulation (activation or repression) exerted by the TFs on their targets^38^, allowing the score to reflect the inferred relative protein activity rather than just the TF’s mRNA level, which provides a more functional and mechanistic estimate of transcriptional control. For our analysis, we require the regulons to include at least 25 target genes. The regulons are ranked based on the gene expression levels of their target genes. Positive scores suggest increased relative protein activity, whereas negative scores indicate decreased activity. Regulons were included in the final IMPREG signature if they: (1) exhibited significantly higher activity in non-responders compared to responders in at least 3/4 of the immunotherapy discovery datasets (two-tailed unpaired *t*-test, *p* < 0.05, Supplementary Dataset 7) with no reverse prediction (i.e., higher activity in responders in any dataset), and (2) achieved an average AUROC ≥ 0.7 for predicting resistance across the four datasets, calculated as the mean AUROC of the regulon across IMvigor210, ISPY2, Kim, and Gide (Fig. 1a). The 10 regulons meeting these criteria were averaged to yield a composite IMPREG score with an average AUROC of 0.79 across the four datasets (Supplementary Fig. 2b). The target genes of the IMPREG regulons are detailed in Supplementary Dataset 9.

### Immune cell deconvolution and computation of immune and cell type-specific signatures

To investigate immune-related markers and gene sets across diverse cancer datasets, we integrated transcriptomic and clinical data from multiple sources, including publicly available repositories. Quantile-normalized gene expression data were obtained from various datasets, encompassing both RNA-seq and microarray platforms, and clinical metadata were sourced from corresponding files or published supplementary materials. Immune scores were calculated for predefined gene sets, including immune-related pathways, cell-type markers, and transcriptional regulon activity, using the transcriptomic data. Cell-type-specific gene sets are compiled from publicly available resources, such as MSigDB^84^ and PanglaoDB^85^. CIBERSORT (v1.04) was applied to expression data to estimate immune cell fractions^48^. The immune scores were merged with clinical metadata to generate comprehensive datasets for downstream analysis. To evaluate the pathway enrichment characteristic of the signatures, and functional association of the pathways, we used the Concept Signature Enrichment Analysis (CSEA)^51^ implemented in the IndepthPathway tool we developed^50^. Hallmark (h.all.v7.5.1) and canonical pathway (c2.cp.v7.5.1) gene sets were chosen as the reference, and *p*-value < 0.05 defines significant pathways. The resulting top pathways are disambiguated via correcting the crosstalk effects between pathways^50^. The pathway network was visualized using the “igraph” R package (ver. 2.1.4).

### Analysis of single-cell RNA-seq datasets

To assess the association between cancer-specific regulon activity and cell-type-specific gene expression signatures across diverse cancer types, we retrieved a Pan-cancer scRNA-seq compendium from Cancer Cell Atlas^52^, which uses an integrated pipeline. The raw gene expression matrix was processed to generate counts-per-million (CPM) and rank-transformed matrices. Cells were retained if they expressed more than 1500 genes with at least 1 read per gene and had mitochondrial gene content below 25%. Genes were retained if they were detected with at least 2 reads in more than 1 cell. After applying these filters, the remaining matrix was normalized to CPM by dividing each gene’s expression by the total reads per cell, scaling to 1 million reads, which was used for downstream analyses, including regulon scores and cell-marker analysis. IMPREG activity scores were calculated based on the average activity of a predefined set of transcription factors. Gene expression signatures were analyzed to identify cells with high activity in predefined signatures, such as immature central neuron, myofibroblast, and endothelial (Supplementary Dataset 10). Cells were classified as IMPREG-high if their IMPREG scores exceeded the threshold defined as the median score plus the MAD (scaled by a constant of 1). The IMPREG-high ratio was calculated as the proportion of IMPREG-high cells in each cell type of each sample relative to the total number of cells in all cell types. Gene set scores representing distinct cell-state signatures were similarly classified as high or low using the same median-plus-MAD thresholding approach. Cell state-specific high score ratios were calculated as the proportion of cells with a high score for each cell-state signature relative to the total cell count in the sample. Cells were further classified as both IMPREG-high and high for specific cell state score if both their IMPREG score and the respective cell-state signature score both exceeded their thresholds. Cell type-specific ratio counts were computed as the proportion of cells within each specific cell type relative to the total cells in the sample. To ensure robust analysis, samples with fewer than 100 cells were excluded. The results were aggregated for the tumor samples with at least 100 cells sequenced and exported for downstream analysis (Supplementary Dataset 11).

### Identification of IMPREG subtype-specific cell-state signatures

To identify cell-state signatures associated with each IMPREG subtype, we systematically screened 1265 cell-state marker signatures compiled from MSigDB, CellMarkerDB, and PanglaoDB against IMPREG scores across the pan-cancer scRNA-seq compendium. For each sample, cell type, and cell-state signature, Pearson correlations with IMPREG scores were computed. Signatures were then evaluated per IMPREG subtype (malignant, fibroblast, endothelial) by calculating the trimmed mean correlation (trim = 10%) across samples within the corresponding cellular compartment. Subtype specificity was assessed using two-sided *t*-tests comparing IMPREG–signature correlations in the index subtype versus all other subtypes; only signatures achieving *p* < 0.01 were retained. From this filtered set, compartment-matched signatures were prioritized (e.g., endothelial-derived signatures for the endothelial subtype) to ensure biological coherence with the cellular origin of each IMPREG subtype. Final signatures were further validated in independent bulk TCGA pan-cancer RNA-seq data. The complete analytical results for all 1265 signatures are provided in Source Data.

### Analysis of spatial transcriptomic datasets

Raw count tables were normalized to counts-per-million (CPM). Low-quality spots were excluded based on: (i) fewer than 1500 detected genes, (ii) greater than 25% mitochondrial RNA content. After spot filtering, genes were filtered to reduce sparsity by retaining only those with ≥2 counts in at least 2 spots. Marker set enrichment scores were calculated using Singscore. Cell types were annotated via ScType, utilizing a curated marker database (Supplementary Dataset 12). Non-immune spots were further stratified based on IMPREG score using a semi-supervised K-means clustering approach. A decision boundary—defined as the midpoint between cluster centers—classified spots into IMPREG-Low or IMPREG-High. The latter were sub-classified into Endothelial, Myofibroblast, or Immature Central Neuronal subtypes based on the maximum ratio relative to their respective adaptive signature thresholds. Spatial Neighborhood Fraction was quantified via nearest-neighbor analysis using the FNN (v1.1.4.1) package: The proportion of cells within a radius of 2.5 times the median neighbor distance. To evaluate functional gradients, dbscan (v1.2.3) clustering (epsilon = 800 px, min Pts = 5) identified IMPREG-high “hotspot” peaks. IMPREG-low non-immune spots were binned into three radial zones relative to these peaks: proximal (0–100 µm), mid (100–500 µm), and distal (500–900 µm). Intercellular signaling was inferred using CellChat (v1.6.1). Ligand–receptor interactions between IMPREG-high subtypes and the tumor and stromal components of the TME were modeled using CellChat’s “triMean” probability method, requiring ≥10 spots per group per slide. Significance was assessed by default permutation-based testing (*p* < 0.05), with per-slide p-values aggregated via Fisher’s combined probability method and Benjamini–Hochberg FDR correction. Interaction prevalence was quantified as the fraction of eligible slides detecting a given ligand–receptor pair, and interaction strength was measured by mean communication probability across slides, which was also used to rank dominant signaling axes within each subtype.

### Statistical analysis

Unpaired *t*-tests were used to compare IMPREG between treatment responder and non-responder cohorts. Two-tailed tests were applied to analyze the discovery datasets, including IMvigor210, ISPY2, STAD (Kim), and melanoma (Gide), while one-tailed tests were used for the validation datasets to test directional hypotheses, to provide greater statistical power, particularly for clinical trial datasets with small sample sizes. Paired *t*-tests were used when measurements were matched within the same samples or subjects across conditions, whereas unpaired *t*-tests were used for independent groups. Log-rank tests were employed to compare statistical differences in survival distributions between groups, and Kaplan-Meier plots were used to visualize survival differences. Receiver operating characteristic (ROC) curves were utilized to evaluate the outcome prediction performance of IMPREG. For datasets with response data categorized by RECIST criteria, we classified Complete Response (CR) and Partial Response (PR) as responders, while Stable Disease (SD) and Progressive Disease (PD) were categorized as non-responders. In datasets where pathological clinical response (pCR) data were available, we classified pCR cases as responders and no-pCR cases as non-responders. For cohorts where response and non-response definitions were specific to the clinical trial protocols, we adhered to the original trial definitions to ensure consistency. Patients are stratified into three IMPREG levels; IMPREG-low, IMPREG-intermediate, and IMPREG-high, based on median and median absolute deviation (MAD) thresholds. The groups were defined as follows: IMPREG-low (< Median − MAD), IMPREG-intermediate (≥ Median − MAD and ≤Median + MAD), and IMPREG-high (> Median + MAD). A constant of 1 was applied for MAD calculation. RR was defined as the percentage of responder patients within each group, with the total number of patients referring to the size of the specific group, not the entire dataset. Spearman’s correlation was used to assess the association between IMPREG and immune infiltration. All statistical analyses were conducted using R (v4.5.1) or GraphPad Prism (v10.2.3) software.

### Computational analyses

We assessed whether IMPREG represents a transcriptionally independent axis by evaluating its orthogonality to five canonical latent tumor programs using TCGA pan-cancer RNA-seq. Spearman correlation clustering of 17 canonical features and PCA on six representative features (IMPREG, T-effectors, Pan-F.TBRS, proliferation, TMB, prior therapy) quantified separability. Interaction testing was performed using likelihood ratio tests comparing a main effects model (Response ~ IMPREG + Modifier) to a model including the interaction term (Response ~ IMPREG + Modifier + IMPREG:Modifier). A significant likelihood ratio test indicates that the effect of IMPREG on response differs across levels of the modifier variable. Benchmarking against established biomarkers used AUROC and AUPRC from single-variable logistic regression with Wilcoxon signed-rank comparison. Independent predictive value was further tested in multivariable models jointly including IMPREG, TMB, CD274, IFNg-related and T-inflamed signature. Finally, enrichment of the neuronal IMPREG subtype in metastasis was assessed using purity-stratified analysis, excluding neuronal-lineage tumors.

### Reporting summary

Further information on research design is available in the Nature Portfolio Reporting Summary linked to this article.

## Supplementary information

Supplementary Information

Description of Additional Supplementary Files

Supplementary Dataset 1

Supplementary Dataset 2

Supplementary Dataset 3

Supplementary Dataset 4

Supplementary Dataset 5

Supplementary Dataset 6

Supplementary Dataset 7

Supplementary Dataset 8

Supplementary Dataset 9

Supplementary Dataset 10

Supplementary Dataset 11

Supplementary Dataset 12

Reporting Summary

Transparent Peer Review file

## Source data

Source Data

## Data Availability

The RNA-seq gene expression data of the UPMC TNBC cohort generated in this study have been deposited in the Gene Expression Omnibus (GEO) under accession code GSE312235. Source data are provided with this paper. The following publicly available datasets were used but not generated in this study. TCGA pan-cancer bulk RNA-seq data were obtained from the UCSC Xena browser (https://xenabrowser.net). GTEx bulk RNA-seq data for healthy tissues were obtained from the Human Protein Atlas portal (https://www.proteinatlas.org). Single-cell RNA-seq data from the pan-cancer compendium (3 CA) were obtained from the Curated Cancer Cell Atlas (https://www.weizmann.ac.il/sites/3CA/). ICGC breast cancer bulk RNA-seq data were obtained from the ICGC Data Portal (https://dcc.icgc.org/). Pan-metastatic bulk RNA-seq data were obtained from dbGaP under accession phs000673.v4.p1 (https://www.ncbi.nlm.nih.gov/projects/gap/cgi-bin/study.cgi?study_id=phs000673.v4.p1). Spatial transcriptomic data were obtained from the HEST-1k compendium (https://huggingface.co/datasets/MahmoodLab/hest). Clinical trial bulk RNA-seq datasets from GEO, the European Genome-Phenome Archive (https://ega-archive.org), and dbGaP (https://dbgap.ncbi.nlm.nih.gov) are detailed with their individual accession codes in Supplementary Dataset 4. Source data are provided with this paper.

## Code availability

The key scripts used in this study have been deposited in GitHub (https://github.com/wangxlab/IMPREG).
