# Supplementary material for: Tumors hijack immune-privileging regulons via distinct cell types to confer T cell desertion and immunotherapy resistance across various cancers
Source: Nat Commun. 2026 May 8;17:6233. doi: 10.1038/s41467-026-72538-x (PMC13369864; doi:10.1038/s41467-026-72538-x)
Supplement: Supplementary file 1 — Supplementary Information [file 41467_2026_72538_MOESM1_ESM.pdf]

Supplementary Information

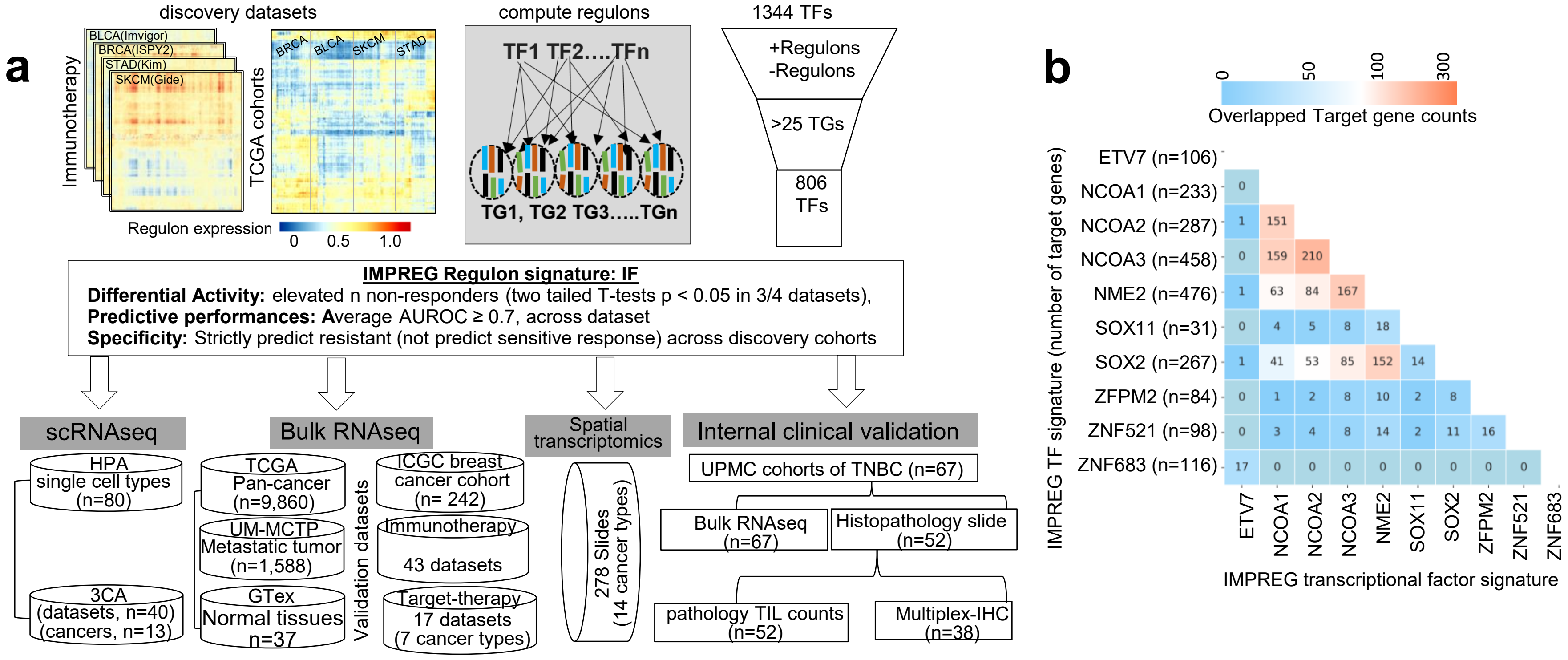

Figure S1. Workflow for Identification, Characterization, and Validation of the IMPREG Signature: (**a**) Analytical Pipeline: IMPREG was derived by computing regulon activity for 1,344 transcription factors across immunotherapy clinical trials and TCGA. Regulons with ≥25 target genes (n = 806) were tested, and IMPREG TFs were selected based on: (i) higher activity in non-responders ($p < 0.05$ in ≥3 of 4 cohorts), (ii) predictive accuracy (mean AUROC ≥ 0.7), and (iii) absence of response-associated patterns. Pathway enrichment defined the functional programs associated with IMPREG. IMPREG expression was characterized in scRNA-seq datasets (HPA: 31 tissues/80 cell types; 3CA: 40 datasets/13 cancers) and validated across TCGA (n = 9,860), GTEx (37 tissues), metastatic tumors (n = 1,588), and ICGC breast cancer (n = 242). Predictive and prognostic associations were evaluated using pan-cancer immunotherapy and targeted therapy datasets. Spatial transcriptomics from 278 slides across 14 cancers, together with the UPMC TNBC cohort (RNA-seq n = 67; histopathology/TILs n = 52; mIHC n = 38), enabled mechanistic evaluation of IMPREG in the tumor microenvironment, including spatial immune profiling, CD8 and PD-L1 IHC, and identification of immune-privileged niches. (**b**) Regulon Overlap Heatmap: Heatmap showing shared target genes among IMPREG transcription factors; cell intensity reflects overlap size. Total target-gene counts are shown in parentheses. c-e: Predictive Performance of IMPREG Scores for Immunotherapy Resistance and Survival Outcomes in Discovery Cohorts.

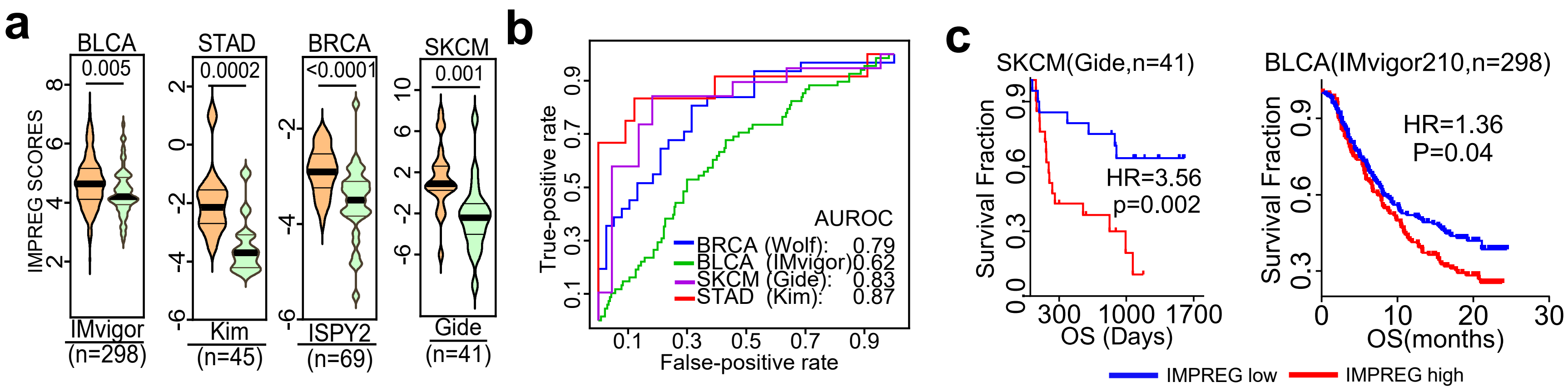

Figure S2. The predictive value of IMPREG signature in the discovery cohort: (a) Boxplots of IMPREG scores in anti-PD-1/PD-L1 discovery cohorts across SKCM, STAD, BRCA, and BLCA, comparing responders vs. non-responders. Each boxplot shows the median, minimum, and maximum values; p-values were computed using two-tailed unpaired t-tests. **(b)** ROC curves showing the predictive accuracy of IMPREG for immunotherapy resistance. IMPREG represents a composite of 10 regulons selected for consistently higher activity in non-responders ($p < 0.05$ in ≥3/4 datasets), strong predictive performance (mean AUROC ≥ 0.7), and absence of inverse associations. AUC values range from 0.5 (random) to 1.0 (perfect). **(c)** Kaplan–Meier curves comparing survival between IMPREG-high and IMPREG-low tumors in the Gide (SKCM) and IMvigor210 (BLCA) trials, demonstrating the prognostic relevance of IMPREG in immunotherapy-treated patients.

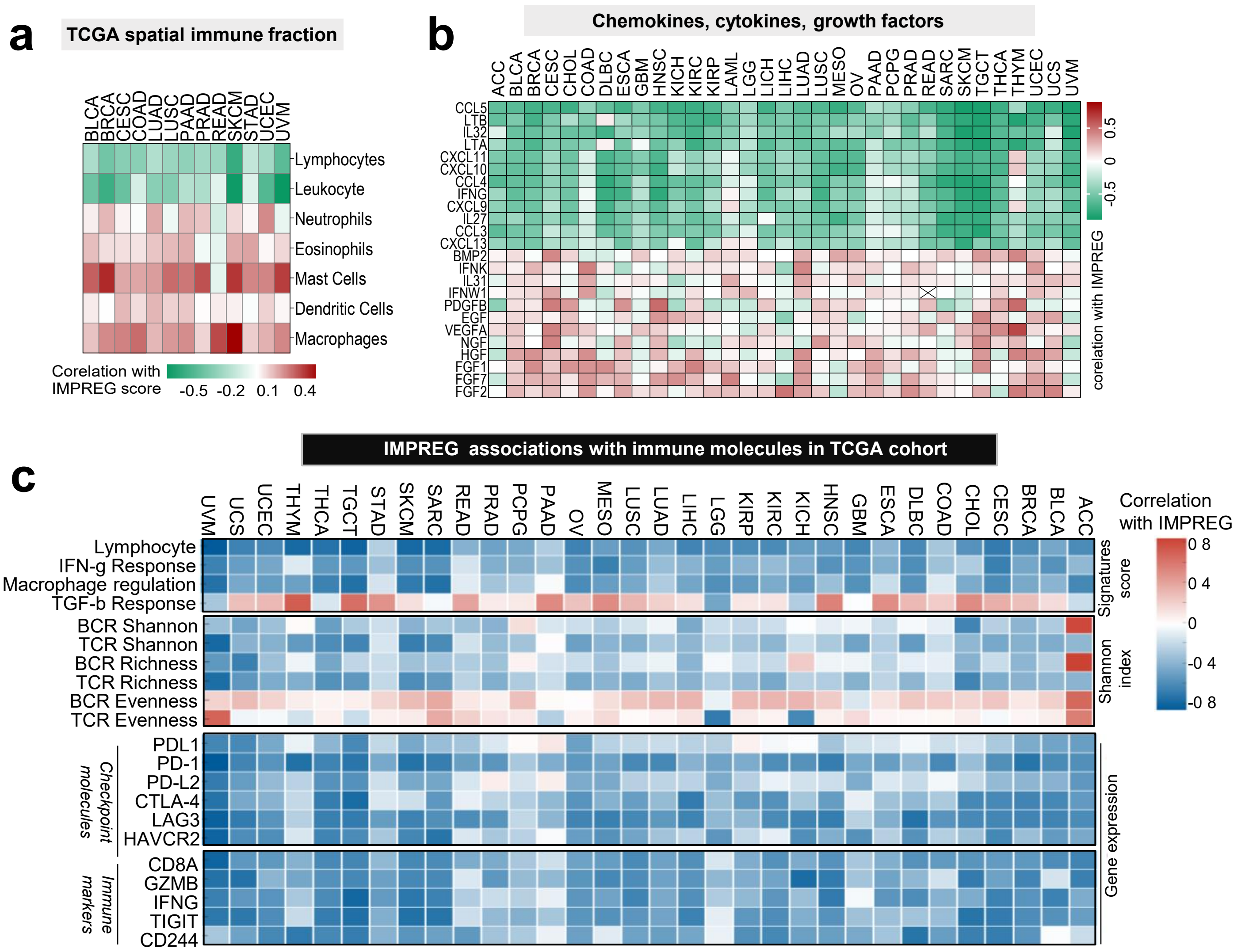

Figure S3. IMPREG is associated with spatial immune desertion, immunosuppressive cytokine profiles, and reduced immune diversity across cancer types. (**a**) Heatmap depicting correlations between IMPREG scores and spatial immune cell fractions. Right: Scatter plot showing a significant negative correlation between IMPREG scores and spatial TIL percentage, indicating an immune-deserted phenotype in high-IMPREG tumors. P values are based on two-sided Spearman rank correlation tests (**b**) Correlation of IMPREG scores with chemokines and cytokines. The heatmap reveals an inverse relationship between IMPREG scores and immune-stimulating factors, while demonstrating a positive association with immunosuppressive cytokines. **(c)** Heatmap of IMPREG Enrichment Correlations with Immune Diversity, Cancer-Related Pathways, and Immune Checkpoint Expression Across TCGA Tumor Types: The heatmap displayed the correlation between IMPREG enrichment and the Shannon diversity index (BCR/TCR-Shannon, richness, and evenness), as well as associations with cancer-related pathways (Lymphocyte IFN-γ Response, Macrophage Regulation, and TGF-β Response). The analysis also includes correlations with gene expression levels of immune checkpoint molecules (PD-L1, PD-1, PD-L2, CTLA-4, LAG3, and HAVCR2) and immune markers (CD8A, GZMB, IFNG, TIGIT, and CD244) across all TCGA tumor types.

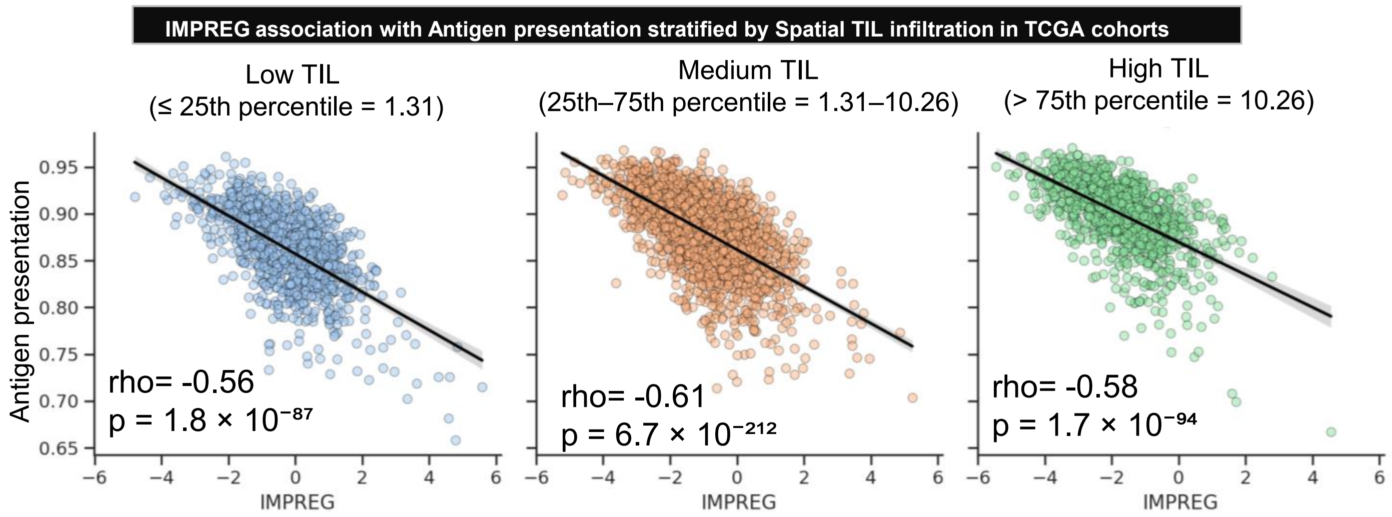

Figure S4. IMPREG association with Antigen presentation stratified by Spatial TIL infiltration in TCGA cohorts. Spatial TIL scores were stratified into three categories using quartiles: Low TIL (≤ 25th percentile = 1.31), Medium TIL (25th–75th percentile = 1.31–10.26), and High TIL (> 75th percentile = 10.26), based on the empirical distribution of the cohort. P values are based on two-sided Spearman rank correlation tests.

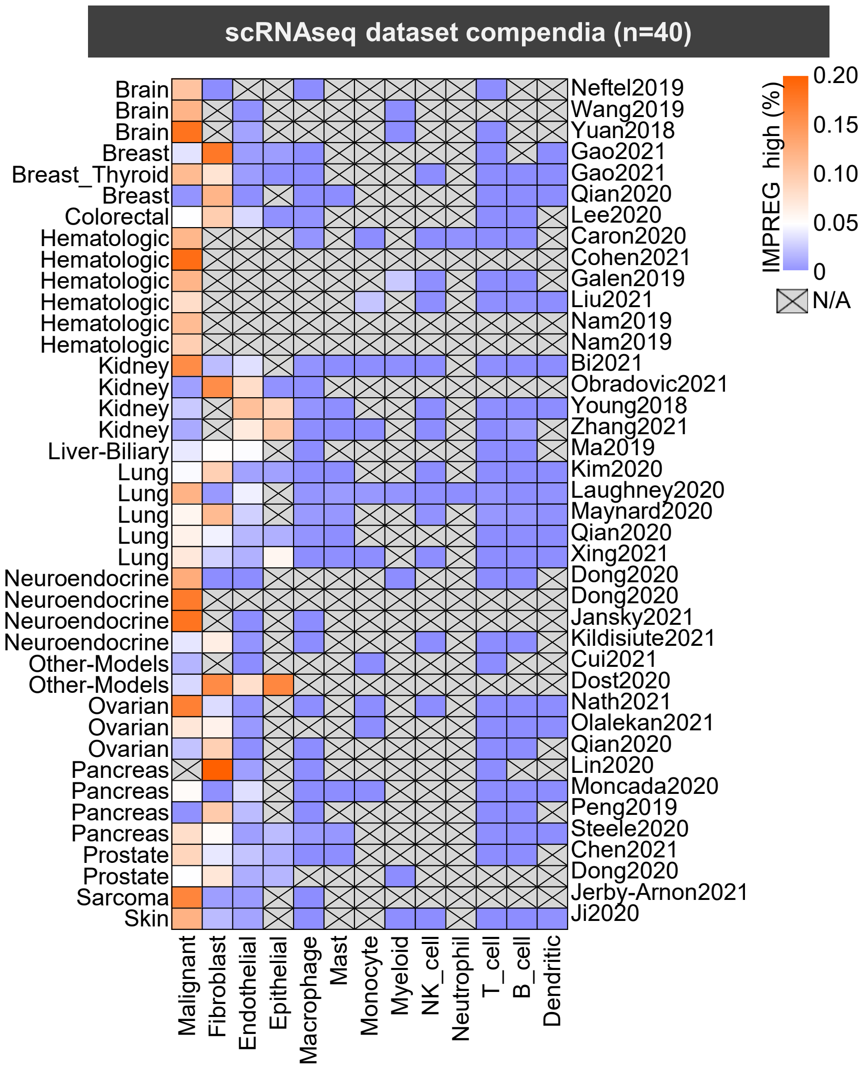

Figure S5: Distribution of IMPREG-High Cells Across Various Cell Types and Cancer Types in Single-Cell Compendium: Heatmap showing the percentage of IMPREG-high cells across major cell types in a single-cell compendium of 40 studies spanning multiple cancer types (BRCA, GBM, HC, NE, NSCLC, OV, PAAD, PRAD, RCC, SKCM, SARC). Cell types include B cells, dendritic cells, monocytes/myeloid cells, neutrophils, NK cells, T cells, macrophages, endothelial cells, malignant cells, fibroblasts, and others. The color scale reflects the proportion of IMPREG-high cells (orange = higher; blue = lower). IMPREG expression is enriched in fibroblasts, endothelial cells, and subsets of malignant cells, with minimal representation in immune cell types such as NK and T cells, highlighting its preferential association with stromal and malignant compartments of the tumor microenvironment.

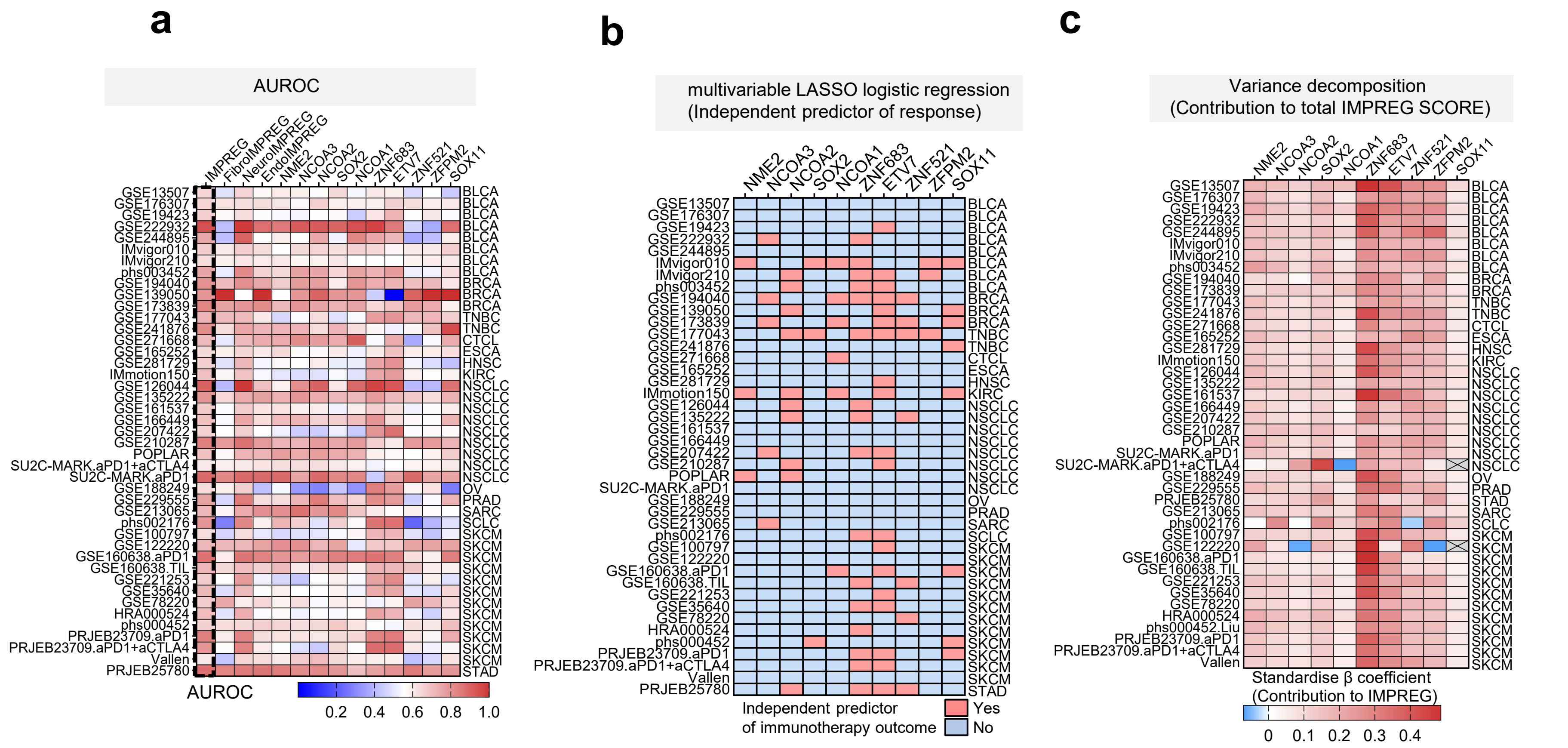

Figure S6. Regulon-level architecture, independence, and predictive performance underlying the IMPREG signature. (A) Heatmap showing the AUROC values for predicting immunotherapy response using the full IMPREG signature, the subtype-restricted versions of IMPREG (neuronal-IMPREG, fibroblast-IMPREG, endothelial-IMPREG), and each individual regulon that composes IMPREG, evaluated across forty independent clinical trial datasets. Color intensity reflects predictive performance (red = higher AUROC; blue = lower AUROC). Across all datasets, the full IMPREG signature demonstrated consistently superior predictive efficacy compared with its subtype-specific versions and the individual regulon components. **(B)** Binarized map of independent regulon predictors of immunotherapy response. For each clinical cohort, a multivariable LASSO logistic regression model was fit using the ten regulons as predictors of binary response. Regulons retained with non-zero coefficients were classified as independent predictors, whereas coefficients shrunk to zero were designated non-predictive. **(C)** Variance decomposition of the IMPREG composite score across datasets. Heatmap of standardized linear model coefficients quantifying the contribution of each regulon component to the IMPREG score.

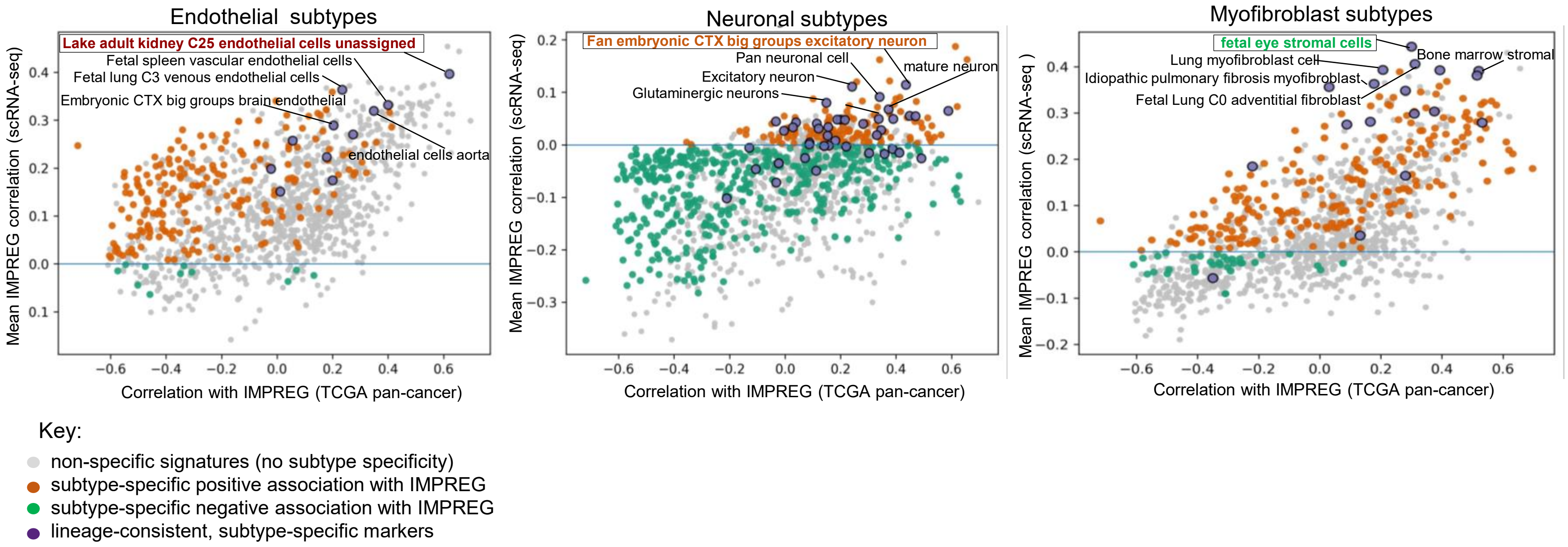

Figure S7. IMPREG-associated cell-state signatures across single-cell and bulk pan-cancer datasets. Scatter plots showing the association of 1,265 curated cell-state signatures (compiled from MSigDB, CellMarkerDB, and PanglaoDB) with IMPREG across the three IMPREG subtypes: endothelial (left), neuronal (middle), and myofibroblast (right). Each point represents one cell-state signature, positioned by its 10% trimmed mean Pearson correlation with IMPREG across pan-cancer single-cell RNA-seq datasets within the indicated cellular compartment (y-axis) and its Pearson correlation with IMPREG in TCGA pan-cancer bulk RNA-seq data (x-axis). Subtype specificity was assessed by comparing IMPREG–signature correlations in the index subtype versus all other subtypes within the pan-cancer single-cell RNA-seq data using two-sided t-tests; signatures with $p < 0.01$ were considered subtype-specific. Points are colored as follows: grey, non-significant signatures ($p > 0.01$); orange, significant positive associations with IMPREG; green, significant negative associations with IMPREG; and purple with black border, compartment-matched signatures prioritized for biological coherence with the subtype of origin, including endothelial-derived signatures in the endothelial subtype, neuronal-lineage signatures in the neuronal subtype, and fibroblast/myofibroblast-derived signatures in the myofibroblast subtype. In each panel, the top five lineage-consistent markers are labeled. The first-ranked signature among these, indicated by a border around its label, was selected as the representative subtype marker for IMPREG classification. Complete results for all 1,265 signatures—including trimmed mean correlations, t-test statistics, and p-values per IMPREG subtype—are provided in the source data.

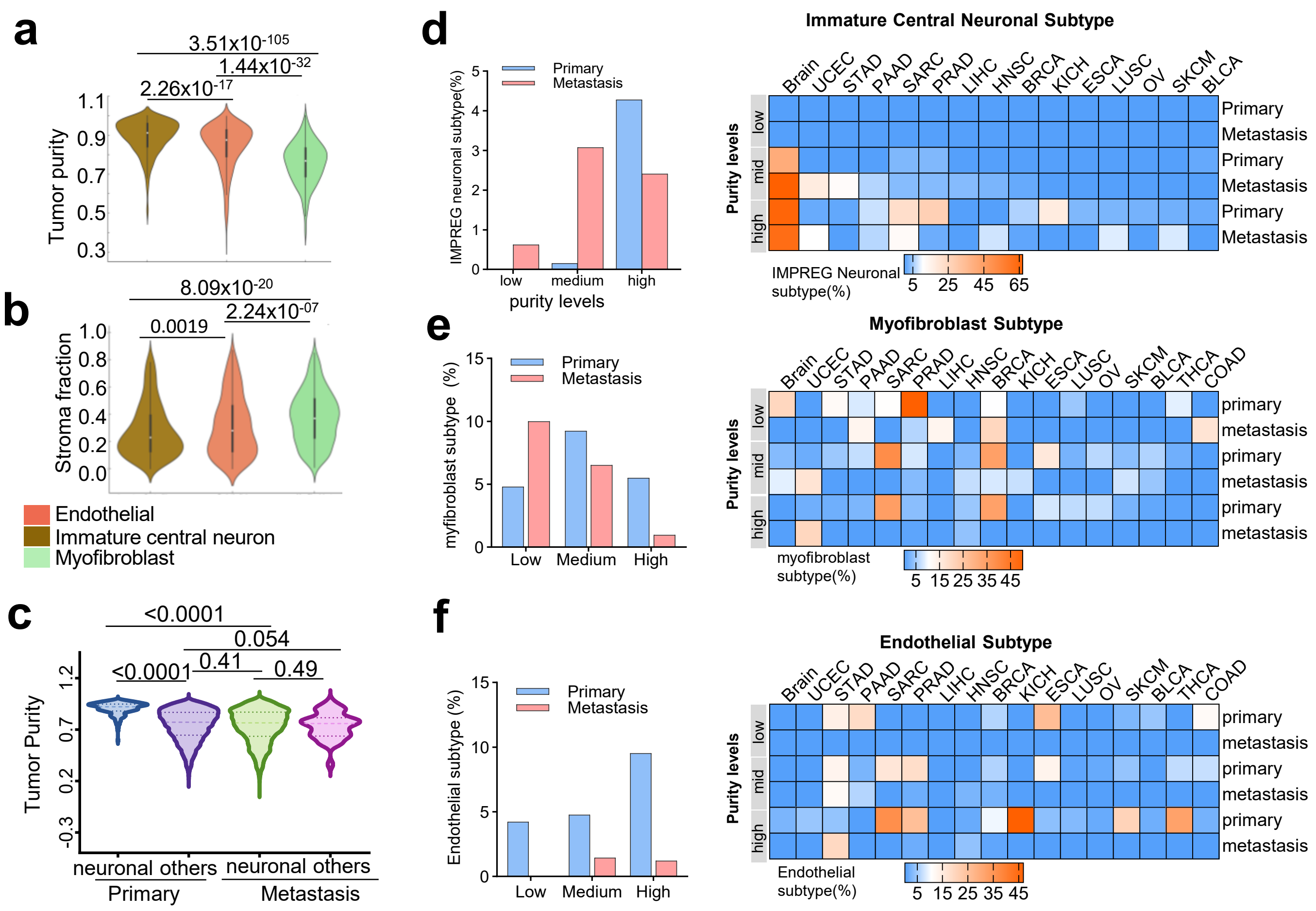

Figure S8. Tumor purity–dependent and –independent enrichment patterns of IMPREG subtypes in primary and metastatic tumors: Boxplots comparing (A) tumor purity and (B) stromal fraction within the three IMPREG-high subtypes (myofibroblast, neuronal, and endothelial-expressed) in TCGA cohort, illustrating the differences in structural composition between tumor cells and stromal components specific to each subtype. (C) Bar plot of purity between primary and metastatic tumor in TCGA primary tumor an UM. Metastatic pan cancer datasets. (D-F) Left panel: Bar plots showing IMPREG subtype frequencies across purity-stratified groups in primary and metastatic tumors, after excluding neuronal-origin cancers. Tumor purity (0–1) was binned into Low, Medium, and High, and the proportions of IMPREG-high neuronal, myofibroblast, and endothelial subtypes were compared between primary and metastatic samples. Neuronal lineage tumors (GBM, PCPG, LGG were excluded from this analysis to avoid confounding effect). Right panel: Heatmaps showing the distribution of neuronal, myofibroblast, and endothelial IMPREG subtypes across cancer types (columns), purity bins (rows), and sample types (primary vs. metastatic). Brain tumors are included here for comparison. Color scales represent the relative frequency of each subtype within each cancer type–purity category.

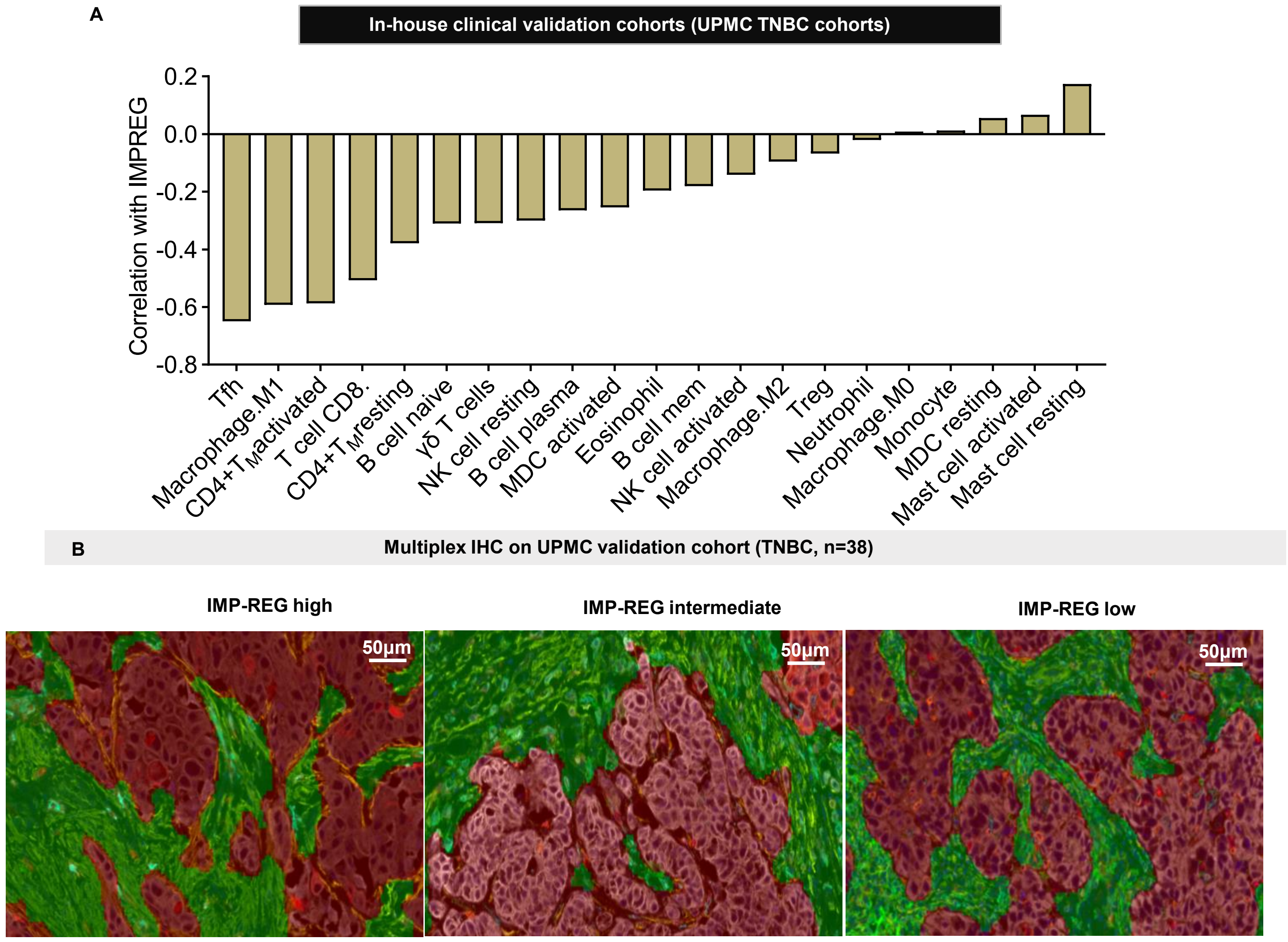

Figure S9: Correlation of IMPREG Signature with Immune Cell Populations and Representative Multiplex IHC Images in the UPMC TNBC Validation Cohort (a) box plot depicting the correlation of the IMPREG signature with CIBERSORT-deconvoluted immune cell populations within the in-house UPMC TNBC (Triple-Negative Breast Cancer) clinical validation cohort. The analysis reveals a consistent negative correlation between IMPREG expression and key immune cell populations, including CD8+ T cells, CD4+ T cells, M1 macrophages, T follicular helper (Tfh) cells, and γδ T cells. (b) Representative multiplex immunohistochemistry (IHC) image showcasing the distinct tumor and stroma compartments from the in-house UPMC TNBC clinical validation cohort.

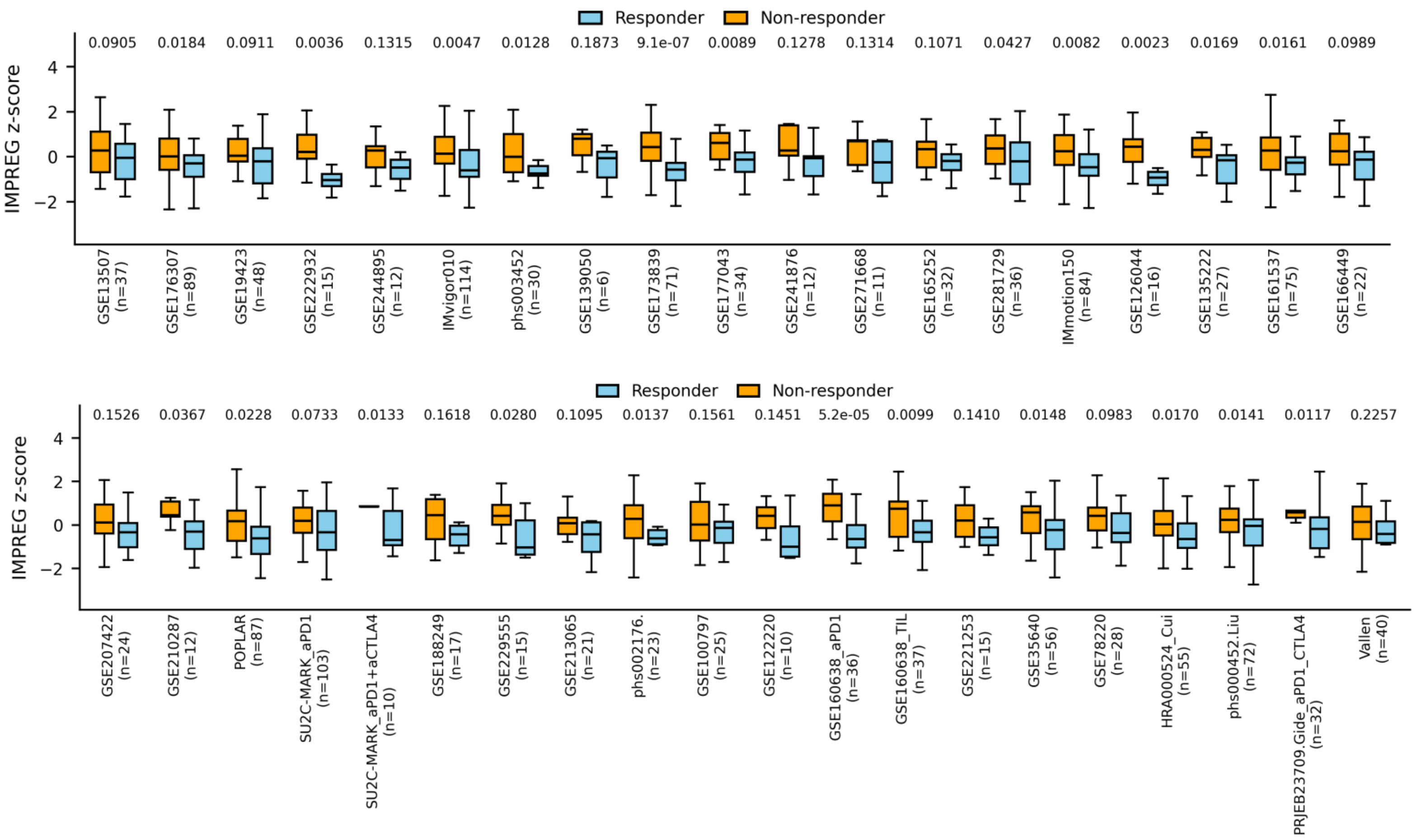

Figure S10. IMPREG score by response status across immunotherapy cohorts. Box-and-whisker plots show IMPREG z-scores within each cohort (per-dataset z-score) for responders (R; blue) and non-responders (NR; orange) across 36 independent immunotherapy validation datasets (39 Treatment Arms). For each cohort, the x-axis label indicates the cancer type, dataset and total sample size (n). Boxes represent the interquartile range (IQR) with the median shown as the central line. For each cohort, significance of higher IMPREG in non-responders was assessed using a one-tailed, unpaired Welch's t-test comparing NR vs R (alternative hypothesis: mean(NR) > mean(R)). The one-tailed p-value for each cohort is printed along a single horizontal line at the top of each panel.

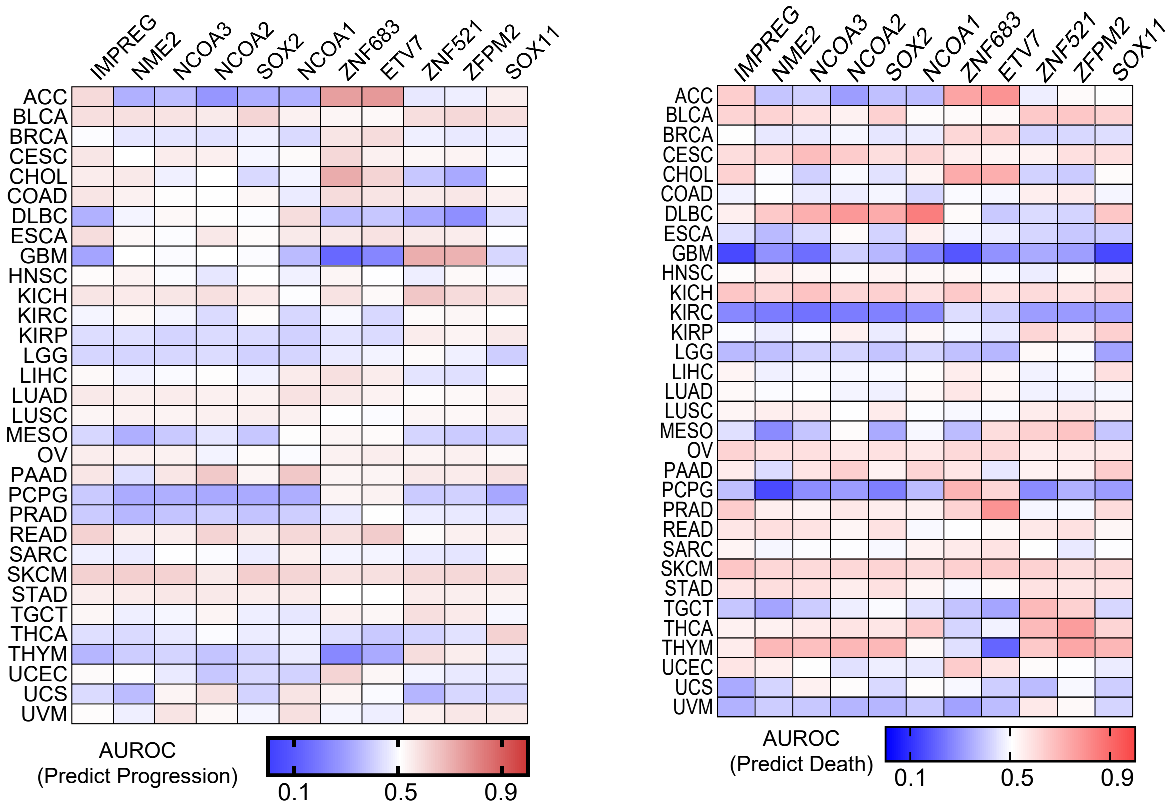

Figure S11. IMPREG regulons are not predictive of disease progression or death in untreated patient cohorts. Heatmap showing the AUROC for the IMPREG composite score and each individual regulon gene across untreated TCGA cancer types, using progression or death status as the endpoint.

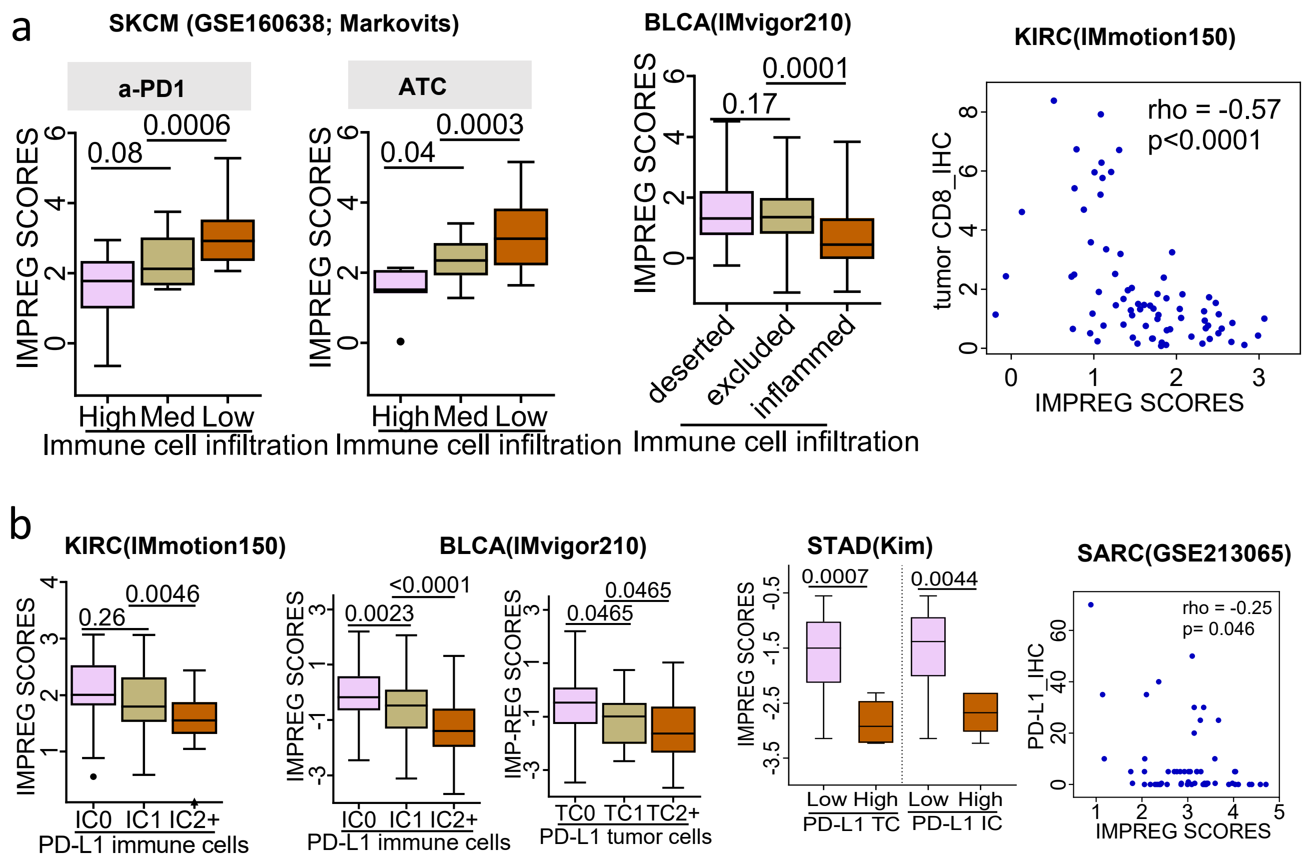

Figure S12: Associations Between IMPREG Scores, Immune Subtypes, CD8 Expression, and PD-L1 Expression Across Multiple Immunotherapy Cohorts **(A)** Boxplots of IMPREG scores across immune subtypes in SKCM (GSE160638; high-, mid-, and low-immune clusters defined by ImmuneScore) and BLCA (IMvigor210; immune-inflamed, -excluded, and -desert phenotypes defined by CD8 IHC and spatial immune patterns). Right panel: scatterplot showing the negative correlation between IMPREG scores and tumor CD8 IHC expression in KIRC (IMmotion150), demonstrating reduced cytotoxic T-cell infiltration in IMPREG-high tumors. **(B)** IMPREG scores across PD-L1 expression clusters in KIRC (IMmotion150), BLCA (IMvigor210), and STAD (Kim) cohorts. The boxplots show median, minimum, and maximum values. Right panel: scatterplot illustrating the association between IMPREG scores and PD-L1 IHC expression in sarcoma patients receiving immunotherapy (GE213065). P-values are based on one-tailed unpaired t-tests. All correlation analyses were performed using Spearman's rank correlation.

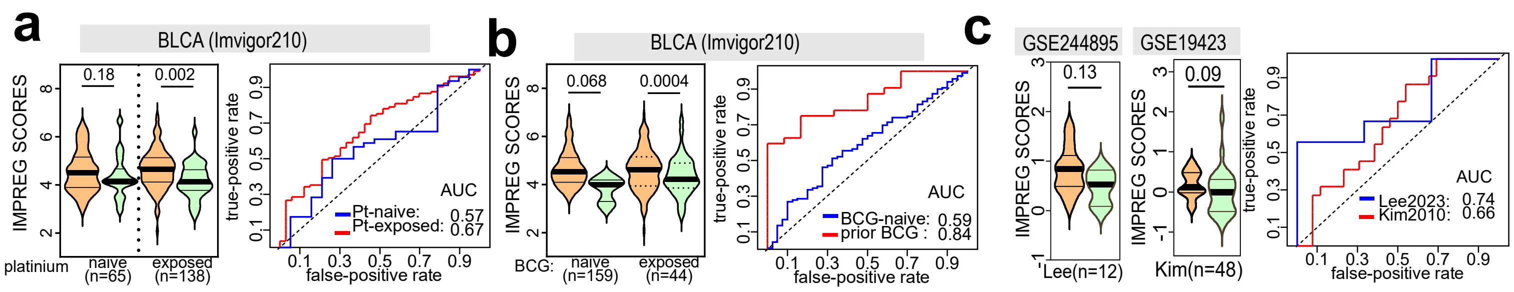

Figure S13. Predictive performance of IMPREG in Bacillus Calmette-Guérin (BCG)-treated bladder cancer (BLCA) patients. (A) IMPREG scores in IMvigor210 BLCA patients stratified by prior platinum treatment vs. platinum-naïve status, with corresponding ROC curves of predictive performance in each group. **(B)** Box plot of IMPREG scores in IMvigor210 patients stratified by prior BCG treatment vs. BCG-naïve status. Boxplots display median, IQR, and min/max values; p-values were computed using one-tailed unpaired t-tests. Right panel shows ROC curves for BCG-treated vs. BCG-naïve groups. **(C)** IMPREG scores (left) and AUC values (right) demonstrating the ability of IMPREG to predict BCG resistance in the Kim and Lee datasets.

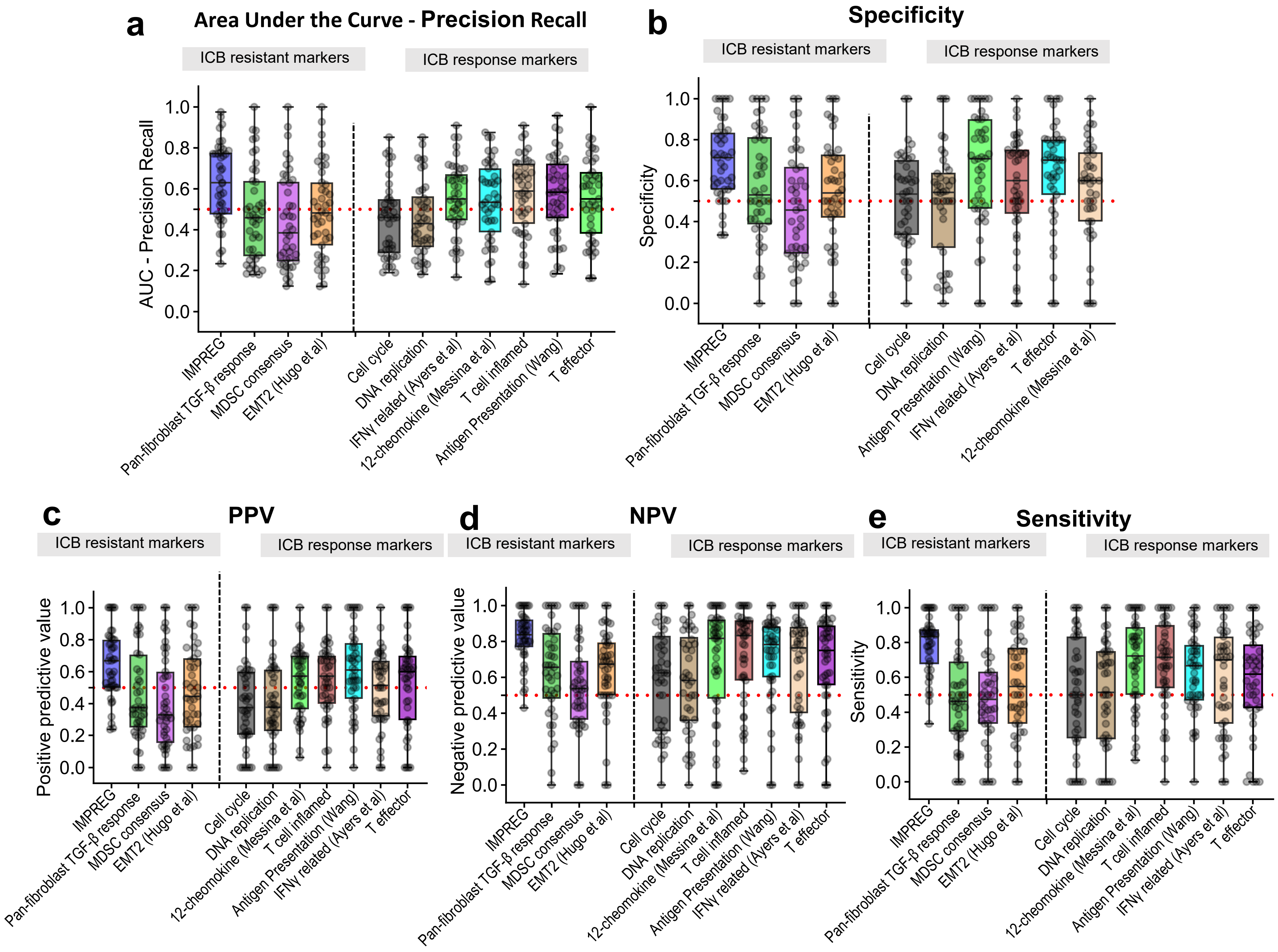

Figure S14. Comparative performance of IMPREG and established immune signatures across 40 immunotherapy clinical trial datasets. (A) Boxplots showing ROC–AUC values for IMPREG and reference ICB-resistant and ICB-response gene signatures across 40 immunotherapy cohorts (43 treatment arms). **(B)** Precision–recall AUC (AUC–PR) distributions for IMPREG and comparator signatures. **(C)** Sensitivity (true-positive rate) across datasets for IMPREG and established immune signatures. **(D)** Specificity (true-negative rate) for IMPREG and comparator signatures. **(E)** Positive and negative predictive value (PPV and NPV) across all datasets for IMPREG and reference immune signatures. All boxplots summarize signature performance across 40 independent ICB clinical trial datasets, with markers grouped into ICB-resistant and ICB-response categories.

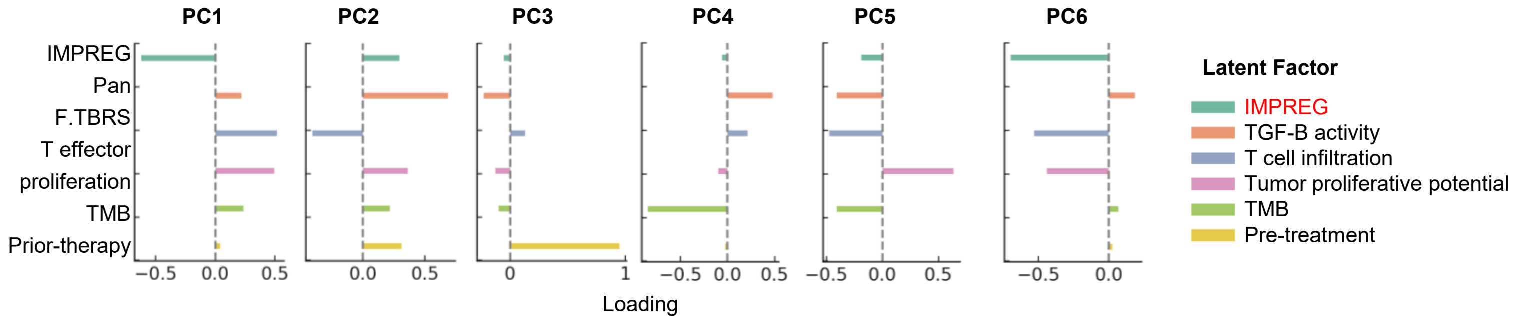

Figure S15: Principal component analysis (PCA) of IMPREG and five canonical latent factor features associated with immunotherapy outcomes. Bar plot depicts the loading coefficients for IMPREG and canonical tumor programs across the principal component (PC1-PC6). IMPREG emerged as the single most dominant contributor to PC1, projected in an orthogonal direction from all other canonical programs, indicating that it captures a unique axis of tumor-state variation not represented by established immunotherapy-associated features.
