# Supplementary material for: Tumors hijack immune-privileging regulons via distinct cell types to confer T cell desertion and immunotherapy resistance across various cancers
Source: Nat Commun. 2026 May 8;17:6233. doi: 10.1038/s41467-026-72538-x (PMC13369864; doi:10.1038/s41467-026-72538-x)
Supplement: Supplementary file 2 — Description of Additional Supplementary Files [file 41467_2026_72538_MOESM2_ESM.pdf]

Description of Additional Supplementary Files

Supplementary Dataset 1: IMPREG Variance Explained (R^2) Across All Immunotherapy Datasets. Tabulates the proportion of IMPREG score variance explained by each of the ten individual constituent regulons, computed via variance decomposition across each immunotherapy cohort analyzed in this study.

Supplementary Dataset 2: AUROC of IMPREG in Age and Gender-based Stratified Analysis. Reports area under the receiver operating characteristic curve (AUROC) values for IMPREG's predictive performance stratified by age and sex (female/male) across immunotherapy cohorts, supporting the independence of IMPREG from demographic variables.

Supplementary Dataset 3: AUROC of IMPREG in Tumor Stage-based Stratified Analysis. Reports AUROC values for IMPREG's predictive performance stratified by clinical tumor stage across immunotherapy cohorts, confirming IMPREG's stability across disease stages.

Supplementary Dataset 4: Overview of Publicly Available Datasets Analyzed in This Study. Provides comprehensive annotations for all 57 clinical trial datasets (40 immunotherapy and 17 targeted therapy) and additional cohorts used in this study, including dataset ID, cancer type, sample size, treatment regimen, clinical endpoint, GEO/EGA/dbGaP accession codes, and source publication references.

Supplementary Dataset 5: Clinical and Demographic Information for the UPMC Internal Validation Cohort. Summarizes patient characteristics for the 67 triple-negative breast cancer (TNBC) cases from UPMC Hillman Cancer Center used for experimental validation, including age, pathological stage, treatment history, and TIL scores.

Supplementary Dataset 6: Details of Antibodies Used for Multiplex Immunohistochemistry (IHC). Lists all primary antibodies included in the Opal 6-plex multiplex IHC panel used for tissue staining, including clone, concentration, catalog number, and supplier information.

Supplementary Dataset 7: AUROC and Two-tailed Test p-Values of Pan-Regulon Across Discovery Cohorts. Reports individual regulon performance statistics (AUROC and p-values from unpaired two-tailed t-tests) across the four immunotherapy discovery datasets (IMvigor210, ISPY2, Gide, Kim) used to select the final ten IMPREG regulons.

Supplementary Dataset 8: AUROC of Pan-Regulon in Sensitivity Analysis Excluding BLCA IMvigor210. Reports regulon performance statistics from the leave-one-out sensitivity analysis confirming that IMPREG signature selection is not disproportionately driven by the IMvigor210 bladder cancer cohort.

Supplementary Dataset 9: Comprehensive List of Target Genes Regulated by Transcription Factors in the IMPREG Signature. Provides the full regulon target gene list for each of the ten IMPREG transcription factors (SOX11, NCOA2, ZNF683, ETV7, ZFPM2, ZNF521, NCOA3, and others), including DoRothEA confidence tier and mode of regulation (activation or repression).

Supplementary Dataset 10: Gene Sets Defining Cell-State Markers for ImmatureCentralNeuron, Myofibroblast, and Endothelial Cell States. Lists the gene signatures used to classify IMPREG-high

tumor spots and single cells into the three IMPREG subtypes (immature central neuronal, myofibroblast, and endothelial) across single-cell and spatial transcriptomic analyses.

Supplementary Dataset 11: Aggregated Output of Individual Cell-Type Analysis Across Pan-Cancer scRNA-Seq Compendium Datasets. Provides per-sample, per-cell-type quantification of IMPREG-high cell fractions, cell-state signature scores, Shannon diversity metrics, and IMPREG subtype classifications across all 40 scRNA-seq datasets spanning 13 cancer types.

Supplementary Dataset 12: Marker Genes Used for Spatial Cell-Type Identification. Lists the curated marker gene sets used by ScType for automated cell-type annotation in the HEST-1k pan-cancer spatial transcriptomic datasets, organized by cell type and annotation category.
