# Supplementary material for: Tumors hijack immune-privileging regulons via distinct cell types to confer T cell desertion and immunotherapy resistance across various cancers
Source: Nat Commun. 2026 May 8;17:6233. doi: 10.1038/s41467-026-72538-x (PMC13369864; doi:10.1038/s41467-026-72538-x)
Supplement: Supplementary file 15 — Reporting Summary [file 41467_2026_72538_MOESM15_ESM.pdf]

nature portfolio

Corresponding author(s): Xiaosong Wang,
Last updated by author(s): Mar 23, 2026

Reporting Summary

Nature Portfolio wishes to improve the reproducibility of the work that we publish. This form provides structure for consistency and transparency in reporting. For further information on Nature Portfolio policies, see our Editorial Policies and the Editorial Policy Checklist.

Statistics

For all statistical analyses, confirm that the following items are present in the figure legend, table legend, main text, or Methods section.

n/a	Confirmed	
☐	☒	The exact sample size (*n*) for each experimental group/condition, given as a discrete number and unit of measurement
☒	☐	A statement on whether measurements were taken from distinct samples or whether the same sample was measured repeatedly
☐	☒	The statistical test(s) used AND whether they are one- or two-sided<br>*Only common tests should be described solely by name; describe more complex techniques in the Methods section.*
☐	☒	A description of all covariates tested
☐	☒	A description of any assumptions or corrections, such as tests of normality and adjustment for multiple comparisons
☐	☒	A full description of the statistical parameters including central tendency (e.g. means) or other basic estimates (e.g. regression coefficient) AND variation (e.g. standard deviation) or associated estimates of uncertainty (e.g. confidence intervals)
☐	☒	For null hypothesis testing, the test statistic (e.g. *F*, *t*, *r*) with confidence intervals, effect sizes, degrees of freedom and *P* value noted<br>*Give P values as exact values whenever suitable.*
☒	☐	For Bayesian analysis, information on the choice of priors and Markov chain Monte Carlo settings
☒	☐	For hierarchical and complex designs, identification of the appropriate level for tests and full reporting of outcomes
☐	☒	Estimates of effect sizes (e.g. Cohen's *d*, Pearson's *r*), indicating how they were calculated

Our web collection on statistics for biologists contains articles on many of the points above.

Software and code

Policy information about availability of computer code

Data collection	Single-cell RNA-seq (scRNA-seq) data from normal tissues and tumor samples were obtained from the Human Protein Atlas (HPA) and the 3CA resource, respectively. Bulk RNA-seq data for TCGA, GTEx, and the ICGC breast cancer cohort were sourced from UCSC Xena, HPA, and ICGC. Metastatic tumor RNA-seq data were accessed through dbGaP. Spatial transcriptomic datasets were obtained from the HEST-1k resource. Clinical annotations and RNA-seq profiles from immunotherapy and targeted-therapy trials were retrieved from GEO, EGA, and dbGaP. Detailed information for all datasets is provided in Supplementary Table 4.<br>Instrument used: Illumina NovaSeq (150 bp paired-end sequencing), Leica Bond RX (automated multiplex IHC staining), Akoya PhenoImager HT (whole-slide multispectral imaging), Qubit fluorometer (library quantification), Agilent Bioanalyzer (library size profiling).
Data analysis	IMPREG was evaluated using t-tests, survival analyses, ROC-based performance metrics, and multivariable regression models. Clinical response was defined using RECIST and, where applicable, trial-specific response criteria. IMPREG was benchmarked against standardized biomarkers across 40 immunotherapy datasets using AUROC, precision–recall AUROC (AUROC-PR), sensitivity, specificity, positive predictive value (PPV), negative predictive value (NPV), and overall accuracy. In addition, IMPREG performance was compared with tumor mutational burden (TMB) and other established biomarkers in harmonized datasets where TMB values were available from the original publications. Immune associations were assessed using Spearman's rank correlation. All statistical analyses were performed in R, with select analyses and visualizations generated in GraphPad Prism.<br>the software used include: R (v4.5.1), DoRothEA (v0.11), VIPER (via DoRothEA), CIBERSORT (v1.04), CellChat (v1.6.1), ScType (custom implementation), singscore (v1.28.1), dbscan (v1.2.3), FNN (v1.1.4.1), IndepthPathway/CSEA (https://github.com/wangxlab/IndepthPathway), igraph (v2.1.4), Seurat (v5.3.0), Rtsne (v0.17), ggplot2 (v4.0.0), ComplexHeatmap (v2.24.1), data.table (v1.17.8), Matrix (v1.7-4), AnnotationDbi (v1.70.0), org.Hs.eg.db (v3.21.0), HGNChelper (v0.8.15), openxlsx (v4.2.8), readxl (v1.4.5), readr (v2.1.5), writexl (v1.5.4), dplyr (v1.1.4), tidyr (v1.3.1), stringr (v1.6.0), pheatmap (v1.0.13), Biobase (v2.68.0), GSEABase (v1.70.1), BiocParallel (v1.42.2), lme4 (v1.1.38), lmerTest (v3.2.0), sf (v1.0.24), arrow (v22.0.0.1), pROC (v1.19.0.1), magick (v2.9.0), SingleCellExperiment (v1.30.1), SpatialExperiment

(v1.18.1), SummarizedExperiment (v1.38.1), zellkonverter (v1.18.0), RColorBrewer (v1.1-3), InForm (v2.8, Akoya Biosciences), GraphPad Prism (v10.2.3), MSigDB (v2022.1), PanglaoDB, GENCODE (v38).

For manuscripts utilizing custom algorithms or software that are central to the research but not yet described in published literature, software must be made available to editors and reviewers. We strongly encourage code deposition in a community repository (e.g. GitHub). See the Nature Portfolio guidelines for submitting code & software for further information.

Data

Policy information about availability of data

All manuscripts must include a data availability statement. This statement should provide the following information, where applicable:

- Accession codes, unique identifiers, or web links for publicly available datasets
- A description of any restrictions on data availability
- For clinical datasets or third party data, please ensure that the statement adheres to our policy

Data availability: The RNA-seq gene expression data of the UPMC TNBC cohort generated in this study has been deposited in Gene Expression Omnibus database, under the accession code GSE312235.
Code availability: The key scripts used in this study have been deposited in Github (https://github.com/wangxlab/IMPREG).

Research involving human participants, their data, or biological material

Policy information about studies with human participants or human data. See also policy information about sex, gender (identity/presentation), and sexual orientation and race, ethnicity and racism.

Reporting on sex and gender	n/a
Reporting on race, ethnicity, or other socially relevant groupings	n/a
Population characteristics	n/a
Recruitment	Formalin-fixed, paraffin-embedded (FFPE) TNBC patient slides (n=67) were obtained from UPMC Hillman Cancer Center.Ethical approval for the use of deidentified human tissues was obtained through the University of Pittsburgh Health Sciences Tissue Bank via a honest broker.Clinical characteristics of patients are provided in Supplementary Table 2.
Ethics oversight	University of Pittsburgh

Note that full information on the approval of the study protocol must also be provided in the manuscript.

Field-specific reporting

Please select the one below that is the best fit for your research. If you are not sure, read the appropriate sections before making your selection.

☒ Life sciences ☐ Behavioural & social sciences ☐ Ecological, evolutionary & environmental sciences

For a reference copy of the document with all sections, see nature.com/documents/nr-reporting-summary-flat.pdf

Life sciences study design

All studies must disclose on these points even when the disclosure is negative.

Sample size	The sample size of the analyses are determined based on the available data provided by published datasets
Data exclusions	Samples with no response data were excluded from each dataset treatment arm
Replication	not replicate
Randomization	Samples were stratified into responder/non-responder groups based on RECIST criteria or trial-specific definitions.IMPREG-high, intermediate, and low groups were defined using the median and MAD thresholds.
Blinding	Tumor-infiltrating lymphocyte (TIL) scoring was performed by a pathologist blinded to the genomic data.

Reporting for specific materials, systems and methods

We require information from authors about some types of materials, experimental systems and methods used in many studies. Here, indicate whether each material, system or method listed is relevant to your study. If you are not sure if a list item applies to your research, read the appropriate section before selecting a response.

Materials & experimental systems

n/a	Involved in the study	
☐	☒	Antibodies
☒	☐	Eukaryotic cell lines
☒	☐	Palaeontology and archaeology
☒	☐	Animals and other organisms
☐	☒	Clinical data
☒	☐	Dual use research of concern
☒	☐	Plants

Methods

n/a	Involved in the study	
☒	☐	ChIP-seq
☒	☐	Flow cytometry
☒	☐	MRI-based neuroimaging

Antibodies

Antibodies used	Detailed in Supplementary Table 6.
Validation	Antibodies were validated and optimized per supplier recommendations and Akoya Bioscience's Opal Assay Development Guide.

Clinical data

Policy information about clinical studies

All manuscripts should comply with the ICMJE guidelines for publication of clinical research and a completed CONSORT checklist must be included with all submissions.

Clinical trial registration	na
Study protocol	na
Data collection	na
Outcomes	na

Plants

Seed stocks	n/a
Novel plant genotypes	n/a
Authentication	n/a
