# Supplementary material for: Tumors hijack immune-privileging regulons via distinct cell types to confer T cell desertion and immunotherapy resistance across various cancers
Source: Nat Commun. 2026 May 8;17:6233. doi: 10.1038/s41467-026-72538-x (PMC13369864; doi:10.1038/s41467-026-72538-x)
Supplement: Supplementary file 16 — Transparent Peer Review file [file 41467_2026_72538_MOESM16_ESM.pdf]

nature portfolio

Peer Review File

Tumors Hijack Immune-Privileging Regulons via Distinct Cell Types to Confer T Cell Desertion and Immunotherapy Resistance Across Various Cancers

Corresponding Author: Dr Xiao-Song Wang

This file contains all reviewer reports in order by version, followed by all author rebuttals in order by version.

Version 0:

Reviewer comments:

Reviewer #1

(Remarks to the Author)
In this study, the authors identified an immune-privileging regulon (IMPREG) signature associated with T cell exclusion and immune checkpoint blockade (ICB) resistance across various cancer types. The IMPREG signature was evaluated using diverse bulk and single-cell RNA sequencing datasets, demonstrating a strong correlation with immunotherapy resistance in ten different cancer types. This was observed across multiple clinical trial datasets involving anti-PD-1/PD-L1 therapies and other immunotherapeutic approaches. While this work serves as a very good example of how carefully re-analyzing publicly available data using newer methods could provide novel insights, we have multiple concerns regarding the analysis objective, rationality for selecting datasets and the presentation of the results, as listed below.

Major comments:
To infer the IMPREG signature, the authors used 4 studies with different types of ICI and combination chemotherapy treatments
blader cancer (BLCA, IMvigor210 cohort) / anti-PD-L1 / n= 203
breast cancer (BRCA, ISPY2 trial) / anti-PD-1+ Paclitaxel / n=69
stomach adenocarcinoma (STAD, Kim cohort) / anti-PD-1 / n=45
melanoma (SKCM, Gide cohort) / anti-PD-1 / n=41

However, the rationale behind selecting these specific datasets is not clear and was not outlined in the manuscript. The selected datasets display an imbalance in terms of cancer types and therapy types. Notably, the IMvigor210 cohort comprises 43% of the patients, while, as Figure 1 illustrates, it shows a relatively low level of active regulons. In contrast, three smaller studies (ISPY2 trial, Kim cohort, Gide cohort) exhibit high regulon activity, representing only 155 patients out of 1128 and 3 studies out of 22. This imbalance could be a significant concern, especially considering the authors employed an unusual method for dividing the datasets into train and test groups. The size of the train group should be larger for robust analysis.

The process by which the authors selected the 10 regulons for the IMPREG signature is not explained. According to the Statistical Analysis section, t-tests were conducted to compare IMPREG between treatment responder and non-responder cohorts, but these results were not provided.
Figure 1 reveals varying regulon activities across studies, which is expected given the diverse immune responses to ICI therapy among cancer types. The analysis objective is unclear. For a comprehensive pan-cancer analysis, a larger train group should be considered, or the focus should be narrowed to a specific cancer type.

The manuscript claims that “the IMPREG score demonstrated AUROC values between 0.62 and 0.87, with an average of 0.72 for predicting resistance.” Many studies have already shown a relationship between tumor microenvironment (TME) composition and immunotherapy response. It is necessary to contextualize these findings within the existing literature and clarify whether the IMPREG signature offers any novel or complementary predictive value. For example, does it predict response independently of tumor mutational burden (TMB) or previously established signatures, such as those related to IFNG (e.g., Ayers et al.)?

Minor comments:
Line 98: Liu et al. is cited, but Gide et al. is used.
Lines 101–103: The text states that “regulons are not broadly indicative of poor clinical outcomes.” However, the analysis only tested the association with disease-free survival (DFS) in TCGA. To support this claim, additional clinical outcomes (e.g., overall survival [OS]) should be evaluated.
Figure 1B: Please fix the color scale.
Line 118: Figure 1B shows a positive correlation between IMPREG and CD4 memory resting T cells, yet the text describes a correlation with activated memory CD4+ T cells. Please clarify or correct the discrepancy.
Figure 1C (left panel) and Figure 3B: Include Spearman’s rho and the corresponding p-value on these panels.
Line 139: The manuscript states: “Our findings revealed that IMPREG expression is strikingly elevated in immune-privileged tissues, such as the retina, brain, central nervous system (CNS), and ovary.” However, the ovary is not typically considered an immune-privileged site. Additionally, there are positive correlations with the endometrium and heart, which are also not immune-privileged. Please review and revise this claim.
Line 172: “In brain cancers, hematologic malignancies, 173 neuroendocrine tumors, skin cancers, and synovial sarcomas, malignant cells exhibited the highest levels of 174 IMPREG expression”. This finding seems biased by the underlying cell types that are present in the dataset. For example, the datasets for hematologic malignancies do not contain endothelial or fibroblasts cells. Therefore, malignant cells by default upregulate the IMPREG signature. As a result, this conclusion is misleading, and the effect of cell abundance in the underlying dataset needs to be controlled to avoid bias.

Line 223: “This notable enrichment suggests a potential selection for neuronal-like transcriptional programs during metastatic progression”. Given that the neuronal cell signature correlates with tumor purity, this observation might simply reflect increased tumor purity at metastatic sites rather than an active selection process. Evidence is needed to exclude the possibility that this is an artifact of tumor purity.
Figure 6A: The IMPREG score appears higher in responders, which seems to be a labelling error. Please verify and correct this.
Line 334: The claim “It suppresses antigen presentation” is not supported by the data. While the IMPREG score is negatively correlated with antigen presentation, this likely reflects an exclusion of T cells from the tumor rather than a direct suppression of antigen presentation. Please reconsider this statement.

The authors primarily use AUROC to determine the accuracy of the IMPREG signature for predicting response. However, including an additional metric, such as AUPRC, is necessary to ensure that any class imbalance is not confounding the result.

Supplementary file. Figure S3: Add the figure legend

Reviewer #2

(Remarks to the Author)
I co-reviewed this manuscript with one of the reviewers who provided the listed reports. This is part of the Nature Communications initiative to facilitate training in peer review and to provide appropriate recognition for Early Career Researchers who co-review manuscripts.

Reviewer #3

(Remarks to the Author)
Comments for the Author
Author identified IMPREG via clinical data analysis, linked to ICB resistance in cancers. IMPREG fosters immunosuppression by hindering antigen presentation, lowering pro-inflammatory cytokines, and depleting T cells. It's highly expressed in immune privileged organs (retina, brain, gonads), suggesting tumors exploit these mechanisms to evade immunity. IMPREG acts through malignant cells, fibroblasts, or endothelial cells in the tumor microenvironment, with expression varying by cell type and tumor. During metastasis, neuronal-like IMPREG subtypes rise while endothelial subtypes decline. In TNBC patients, high IMPREG tumors show T cell rejection and myofibroblast-rich environments. IMPREG predicts ICB response across datasets and correlates with sensitivity to targeted therapies (e.g., EGFR inhibitors, anti-angiogenics) in NSCLC. Findings support IMPREG as a biomarker and therapeutic target. Concerns remain to be addressed.

1. The background introduction of immune checkpoint blockade therapy is comprehensive, but it could further highlight the main challenges of current immunotherapy, such as low patient response rates and side effects, to emphasize the necessity and urgency of this study. When introducing the concept of immune privilege, it is recommended to supplement with specific examples of immune escape mechanisms in classical immune-privileged organs to enhance readers' understanding.

2. When describing the discovery of the IMPREG signature, the analysis methods and data sources are detailed. However, it is recommended to more clearly explain why these specific analysis tools (such as Dorothea) were chosen and their advantages over other methods. In addition, when validating the predictive utility of the IMPREG signature, it is suggested to add some validation experiments at the biological mechanism level, in addition to statistical indicators (such as AUROC), to

further confirm its role in immune evasion.

3. Single-cell RNA sequencing data analysis is a crucial part of the article. When showing the distribution of IMPREG expression in different cell types, more detailed information about cell type identification markers and the basis for their selection can be provided to help readers better assess the accuracy of cell type classification. Additionally, the spatial distribution characteristics of IMPREG-high cells in the tumor microenvironment can be explored in combination with more spatial transcriptomics data to reveal their specific localization patterns in tumor tissues.

4. The article identifies thee IMPREG-high tumor subtypes based on cell states, but when discussing their biological significance, it is recommended to further analyze the unique mechanisms of each subtype in tumor initiation, progression, and metastasis.

5. When analyzing clinical trial data, in addition to focusing on treatment response and survival rates, it is suggested to perform stratified analyses based on patient clinical characteristics (such as age, gender, and tumor stage) to explore whether there are differences in the predictive value of the IMPREG signature in different patient groups. Additionally, for the application of IMPREG in predicting targeted therapy sensitivity, it is recommended to further clarify its potential clinical application scenarios and usage processes to provide more operational guidance for clinicians.

6. The discussion section comprehensively summarizes and analyzes the research results. However, when comparing with other research findings, it is suggested to more deeply explore the innovation and advantages of this study, as well as the reasons for the differences from previous research results. Additionally, for the potential development direction of the IMPREG signature in future research, it is recommended to propose more forward-looking and innovative suggestions, such as exploring its application value in combination therapy strategies.

7. The article mentions some research limitations in the discussion section, such as data heterogeneity, but it is recommended to further elaborate on the potential impact of these limitations on the research results and how to overcome them in future research.

Reviewer #4

(Remarks to the Author)
In this manuscript, the authors present a pan-cancer computational analysis of transcriptional pathways associated with immune escape and the lack of response to immunotherapy. Their main claim is the development of a gene-set signature based on transcription factor (TF) regulons that are negatively associated with immune responses, indicating a role in immune evasion and resistance to immunotherapy. Interestingly, the signature appears to be linked to responses to anti-angiogenic therapies and EGFR inhibitors.
While the analysis supports the enrichment of the gene set in tumors with poor immunotherapy responses, its uniqueness and comparison to existing gene sets and scores remain unclear. Additionally, it is uncertain whether this represents a distinct biological entity or if it is capturing a more general phenomenon, which, while clinically relevant, may not constitute a truly novel biological category.
Below, I outline my main comments:
1)What is unique about IMPREG and how does it compare to other gene-sets? I would suggest:
- Conducting a systematic comparison with existing gene sets (e.g., MSigDB, Reactome, TGF-β-related gene sets) to contextualize IMPREG's distinct features.
- Assessing the added value of using an aggregated score versus individual regulons. Can the contribution of each component be broken down to understand how they influence the overall score across cancer types? Can you do this analytically?

2)While the association with negative immunotherapy responses is clear, it remains unclear how IMPREG compares to other molecular features linked to poor immunotherapy outcomes.
-Specifically, what is its added value relative to tumor mutational burden (TMB) or T-cell infiltration levels (high vs. low)?
- Is IMPREG an orthogonal marker, or does it represent a sixth latent factor (see PMID: 39266764)?
-Additionally, are other gene sets associated with negative immunotherapy responses also linked to increased sensitivity to anti-angiogenic therapies or EGFR inhibitors? Or is this feature unique to IMPREG?

3) I would finally recommend to focus the question of the manuscript (more clinical applicability or biological novelty) and weight the manuscript accordingly.

Version 1:

Reviewer comments:

Reviewer #1

(Remarks to the Author)
Overall, the revision has substantially improved the clarity and rigor of the work, and my major concerns from the initial review have been addressed.

In particular, the authors have clarified the rationale for selecting the discovery cohorts and described the discovery and selection criteria for the final regulon set more explicitly. The presentation and interpretation of the TCGA outcome analyses have been strengthened, and multiple sections have been revised to improve accuracy and consistency (including several figure and text corrections). The authors also expanded their benchmarking and discussion to better contextualize the proposed signature relative to existing biomarkers and related immune programs.

Based on the revised manuscript, I believe my concerns have been adequately addressed and I recommend the manuscript for publication.

(Remarks on code availability)

Reviewer #4

(Remarks to the Author)
The authors have addressed most of my comments and concerns, and based on their analyses, IMPREG appears to represent a distinctive fingerprint that could be clinically relevant for predicting immunotherapy response across different cancer types. While the underlying biological mechanisms remain not fully clear, I understand that this is not the primary focus of the study.

I have just one additional comment:

In Figure S7, I would like to see the similarity distribution of all programs compared against the full set of 1,265 programs, with an indication of where the three main contributors (fibroblast, neuronal, and endothelial programs) fall within this overall distribution. In its current form, the figure lacks sufficient context, and it is unclear whether these programs are indeed among the most similar ones or were selectively highlighted.

(Remarks on code availability)

Open Access This Peer Review File is licensed under a Creative Commons Attribution 4.0 International License, which permits use, sharing, adaptation, distribution and reproduction in any medium or format, as long as you give appropriate credit to the original author(s) and the source, provide a link to the Creative Commons license, and indicate if changes were made.
In cases where reviewers are anonymous, credit should be given to 'Anonymous Referee' and the source.
The images or other third party material in this Peer Review File are included in the article’s Creative Commons license, unless indicated otherwise in a credit line to the material. If material is not included in the article’s Creative Commons license and your intended use is not permitted by statutory regulation or exceeds the permitted use, you will need to obtain permission directly from the copyright holder.
To view a copy of this license, visit https://creativecommons.org/licenses/by/4.0/

Reviewer #1 (Remarks to the Author):

***Comment**: In this study, the authors identified an immune-privileging regulon (IMPREG) signature associated with T cell exclusion and immune checkpoint blockade (ICB) resistance across various cancer types. The IMPREG signature was evaluated using diverse bulk and single-cell RNA sequencing datasets, demonstrating a strong correlation with immunotherapy resistance in ten different cancer types. This was observed across multiple clinical trial datasets involving anti-PD-1/PD-L1 therapies and other immunotherapeutic approaches. While this work serves as a very good example of how carefully re-analyzing publicly available data using newer methods could provide novel insights, we have multiple concerns regarding the analysis objective, rationality for selecting datasets and the presentation of the results, as listed below.*

Response: We appreciate Reviewer #1's thoughtful and constructive feedback, which has substantially enhanced the quality of our manuscript. In response, we have comprehensively addressed all concerns regarding dataset selection criteria, regulon identification strategy, analytical approaches, and the clarity of data presentation.

Major comments: *To infer the IMPREG signature, the authors used 4 studies with different types of ICI and combination chemotherapy treatments: blader cancer (BLCA, IMvigor210 cohort) / anti-PD-L1 / n= 203, breast cancer (BRCA, ISPY2 trial) / anti-PD-1+ Paclitaxel / n=69, stomach adenocarcinoma (STAD, Kim cohort) / anti-PD-1 / n=45, melanoma (SKCM, Gide cohort) / anti-PD-1 / n=41. The rationale behind selecting these specific datasets is not clear and was not outlined in the manuscript. The selected datasets display an imbalance in terms of cancer types and therapy types. Notably, the IMvigor210 cohort comprises 43% of the patients, while, as Figure 1 illustrates, it shows a relatively low level of active regulons. In contrast, three smaller studies (ISPY2 trial, Kim cohort, Gide cohort) exhibit high regulon activity, representing only 155 patients out of 1128 and 3 studies out of 22. This imbalance could be a significant concern*, especially considering the authors employed an unusual method for dividing the datasets into train and test groups. The size of the train group should be larger for robust analysis.

Response 1: We appreciate this important point and have now added a dedicated subsection in the Methods ("Selection of Discovery Datasets") to clarify our rationale for dataset inclusion (**Page 12-13 of the revise manuscript**). It is important to clarify that we employed a **consensus discovery approach** to identify immune-privileging regulons, rather than a traditional supervised machine learning workflow involving a split training/testing set. Consequently, the concept of class balance between datasets does not apply in the same way. The four cohorts (IMvigor210, ISPY2, Kim, Gide) were chosen based on predefined criteria available at the initiation of this study: (i) available pretreatment RNA-seq data, (ii) high-quality clinical and response data, (iii) representation of distinct immunotherapy contexts across cancer types, and (iv) being the largest publicly available datasets with these characteristics in their respective indications at the time we initiate this study. To mitigate concerns about sample-size imbalance, each dataset was analyzed independently to avoid dominance by larger cohorts. We acknowledge that the IMvigor210 cohort had limited influence on the derivation of the IMPREG signature; exclusion of this cohort reproduced 9 of the 10 regulons identified (**Supplementary Table S8**). This demonstrates that the discovery process was not driven by dataset size but rather by consistent regulon activation patterns *across diverse immunotherapy contexts*.

***Major Comment 2**: The process by which the authors selected the 10 regulons for the IMPREG signature is not explained. According to the Statistical Analysis section, t-tests were conducted to compare IMPREG between treatment responder and non-responder cohorts, but these results were not provided. Figure 1 reveals varying regulon activities across studies, which is expected given the diverse immune responses to ICI therapy among cancer types. The analysis objective is unclear. For a comprehensive pan-cancer analysis, a larger train group should be considered, or the focus should be narrowed to a specific cancer type.*

Response: We thank the reviewer for this valuable feedback. We have detailed this selection process in Methods (page 12) and **Figure S1**. The t-tests comparing regulons between treatment responder and non-responder cohorts is provided in **Supplementary Table S7**. We clarify that our **analytical objective** was to identify a *convergent* transcriptional program of immune exclusion shared across diverse cancer types, rather than cancer-

specific mechanisms. While regulon activity naturally varies due to biological heterogeneity, our **consensus discovery approach** was specifically designed to filter out this context-specific noise and isolate core regulators of resistance. By requiring regulons to be consistently predictive across 4 diverse discovery cohorts ($p < 0.05$ in ≥3/4 datasets; average AUROC ≥ 0.7), we prioritized robust, pan-cancer signals over cancer-specific features. This intersection strategy serves as a rigorous alternative to training on a single large dataset, ensuring the signature's generalizability and robustness against overfitting to a specific tumor type.

Comments: *The manuscript claims that "the IMPREG score demonstrated AUROC values between 0.62 and 0.87, with an average of 0.72 for predicting resistance." Many studies have already shown a relationship between tumor microenvironment (TME) composition and immunotherapy response. It is necessary to contextualize these findings within the existing literature and clarify whether the IMPREG signature offers any novel or complementary predictive value. For example, does it predict response independently of tumor mutational burden (TMB) or previously established signatures, such as those related to IFNG (e.g., Ayers et al.)?*

Response: We thank the reviewer for this important point. We have expanded the Results and Discussion to contextualize IMPREG within existing markers. First, we directly **benchmarked IMPREG against established biomarkers** including TMB, T-cell effector, and TGF-β signatures **across an expanded compendia of 40 datasets** (**Figure 8a, page 8)**, showing consistent superiority. Second, principal component analysis demonstrates that IMPREG is orthogonal to the five canonical latent tumor factors (TMB, proliferation, EMT etc.), occupying a unique variance axis (page 8, **Figure 8c**). Finally, multivariable Coxph regression analyses confirmed that IMPREG retains significant predictive power for immunotherapy resistance after adjusting for TMB, IFNG-related signature (Ayers et al.), T-inflamed signature, and CD274 expression (**Page 9**, **Figure 8e**). These findings demonstrate its **complementary and independent predictive value** beyond established immune-related signatures.

Minor Comments:

Comment: Line 98: *Liu et al. is cited, but Gide et al. is used.*

Response: We have corrected the citation to "Gide et al., 2019" in the revised manuscript.

Comment: Lines 101–103: *The text states that "regulons are not broadly indicative of poor clinical outcomes." However, the analysis only tested the association with disease-free survival (DFS) in TCGA. To support this claim, additional clinical outcomes (e.g., overall survival) should be evaluated.*

Response: We appreciate this suggestion. We have expanded the TCGA analysis to include overall survival (OS) and disease progression across the TCGA cancer types (**Figure S11**). The results confirm that IMPREG is not broadly prognostic for poor outcomes in untreated patients, reinforcing its specific role as a predictive biomarker for immunotherapy resistance (**Page 8**). To maintain consistency with our AUROC metrics, the heatmap in **Figure 1a** has been updated to reflect AUROC values for predicting disease progression.

Comment: Figure 1B: *Please fix the color scale.*

Response: We thank the reviewer for catching this. We have now corrected the color scale in **Figure 1B**.

Comment: Line 118: *Figure 1B shows a positive correlation between IMPREG and CD4 memory resting T cells, yet the text describes a correlation with activated memory CD4+ T cells. Please clarify or correct the discrepancy.*

Response: We thank the reviewer for catching this discrepancy. We have corrected the text at Line 118 to accurately reflect the data shown in **Figure 1B**, stating that IMPREG positively correlates with CD4 memory resting T cells.

Comment: Figure 1C (left panel) and Figure 3B: *Include Spearman's rho and the corresponding p-value on these panels.*

Response: We have added Spearman's rho and the corresponding p-values directly onto the scatter plots in **Figure 1C** and **3B**.

Comments: *Line 139: The manuscript states: "Our findings revealed that IMPREG expression is strikingly elevated in immune-privileged tissues, such as the retina, brain, central nervous system (CNS), and ovary.* However, the ovary is not typically considered an immune-privileged site. Additionally, there are positive correlations with the endometrium and heart, which are also not immune-privileged. Please review and revise this claim.

Response: We thank the reviewer for the clarification. We agree that the ovary, endometrium, and heart are not traditionally classified as immune-privileged organs. In response, we have revised the statement to note that IMPREG is enriched in classical immune-privileged tissues (retina, brain, CNS) and also elevated in the ovary, endometrium, and heart (Page 4).

Comment: *Line 172: "In brain cancers, hematologic malignancies, neuroendocrine tumors, skin cancers, and synovial sarcomas, malignant cells exhibited the highest levels of IMPREG expression". This finding seems biased by the underlying cell types that are present in the dataset. For example, the datasets for hematologic malignancies do not contain endothelial or fibroblasts cells. Therefore, malignant cells by default upregulate the IMPREG signature. As a result, this conclusion is misleading, and the effect of cell abundance in the underlying dataset needs to be controlled to avoid bias.*

Response: We appreciate the reviewer's insight regarding compositional bias. We agree that in datasets lacking specific stromal populations (e.g., hematologic malignancies), relative expression rankings can be misleading.

To address this, we have refined our analysis to control for cell abundance and composition:

1. We excluded hematologic malignancies from comparative cell-type analyses in **Figure 3a-b** to avoid baseline bias and to illustrate the broader landscape of cancer subtypes. This ensures the bar charts in **Figure 3b** reflect the true landscape of cancer subtypes, not just the "high-diversity" ones.
2. For **Figure 3c-f,** we applied stricter inclusion criteria which exclusively analyze solid tumor samples containing both malignant and stromal (endothelial/fibroblast) populations with a Shannon diversity index ≥0.8.

Comments*: Line 223: "This notable enrichment suggests a potential selection for neuronal-like transcriptional programs during metastatic progression". Given that the neuronal cell signature correlates with tumor purity, this observation might simply reflect increased tumor purity at metastatic sites rather than an active selection process. Evidence is needed to exclude the possibility that this is an artifact of tumor purity.*

Response: We thank the reviewer for this important point. We now performed additional analyses to assess the impact of tumor purity (**Figure S8**). First, we found that primary neuronal tumors exhibited significantly *higher* purity than metastatic neuronal tumors ($p < 0.0001$; **Figure S8c, Page 6**). This counters the hypothesis that metastatic neuronal enrichment is driven by higher purity. Second, to disentangle purity effects, we stratified tumors into low-, medium-, and high-purity tiers. For the **neuronal subtype (Figure S8d)**, we observed a striking decoupling of signature from purity in metastases: while primary tumors showed a strict purity-dependency (restricted to high-purity samples), metastatic tumors exhibited significant neuronal enrichment in medium-purity samples (~3% vs primary ~0.2%). In contrast, the **myofibroblast subtype (Figure S8e)** in metastatic tumors displayed a monotonic decrease with increasing purity, consistent with stromal dilution, while the **endothelial subtype (Figure S8f)** remained low across metastatic tiers. These divergent, non-parallel purity trajectories confirm that the emergence of the neuronal phenotype in metastasis is not a linear function of tumor cellularity but reflects a specific, purity-independent biological selection. To avoid confounding by tissues with intrinsic neuronal programs, the overall neuronal subtype frequency in purity-stratified analyses were performed after excluding tumors of neuronal origin (GBM, LGG, and PCPG) in **Figure S8d-f** left panels.

minor comment *Figure 6A:* ***The IMPREG score appears higher in responders, which seems to be a labelling error****. Please verify and correct this.*

Response: You are correct—Figure 6A had a label inversion. We have corrected the figure and caption; the IMPREG score is now shown higher in **non-responders**, consistent with the text.

Line 334: The claim "It suppresses antigen presentation" is not supported by the data. While the IMPREG score is negatively correlated with antigen presentation, this likely reflects an exclusion of T cells from the tumor rather than a direct suppression of antigen presentation. Please reconsider this statement.

Response: We thank the reviewer for this helpful clarification. We have revised the statement to accurately reflect the data, now noting that IMPREG is *negatively associated with antigen presentation, independent of T-cell infiltration* (see **Page 4**). To address the concern that this might be a bystander effect of T-cell exclusion, we provide three lines of evidence supporting a direct or spatially-coupled relationship: (1) **Independence from Infiltration (Figure S4):** The negative association between IMPREG and antigen presentation machinery remains statistically significant even when analyzing tumors stratified by high vs. low TIL levels (determined based on H&E images), indicating the relationship exists regardless of spatial T-cell abundance. (2) **Cell-Intrinsic Downregulation (Figure 3d):** Single-cell analysis reveals that the downregulation of MHC-I/II genes (e.g., *HLA-A*, *B2M*) occurs *within* the specific malignant, fibroblast, and endothelial cells that express high IMPREG, suggesting an intrinsic regulatory link rather than just an extrinsic lack of immune pressure. (3) **Spatial Suppression Gradients (Figure 5d):** Spatial transcriptomics demonstrates a radial gradient where antigen presentation proficiency is lowest at the IMPREG+ core and increases with distance. This spatial patterning implies that IMPREG+ niches actively create a local suppressive microenvironment, rather than simply existing in immune-deserted regions. (4) **Mechanistic link via target gene analysis:** Pathway analysis of IMPREG regulon target genes reveals that antigen presentation programs are negatively regulated by IMPREG-associated transcription factors, providing a direct molecular basis for this suppression.

Comments: *The authors primarily use AUROC to determine the accuracy of the IMPREG signature for predicting response.* ***However, including an additional metric, such as AUPRC,*** *is necessary to ensure that any class imbalance is not confounding the result.*

Response: we have included AUPRC. We also compare the AUPRC, PPV, NPV, Sensitivity, and Specificity between IMPREG and established existing biomarkers across 40 dataset treatment arms **(Figure S14).**

Comments*: Supplementary file. Figure S3: Add the figure legend*

Response: We have now added the figure legend.

Reviewer #2 (Remarks to the Author):

I co-reviewed this manuscript with one of the reviewers who provided the listed reports. This is part of the Nature Communications initiative to facilitate training in peer review and to provide appropriate recognition for Early Career Researchers who co-review manuscripts.

Response: We appreciate the note and thank the reviewer and co-reviewer for their constructive evaluation and contribution.

Reviewer #3 (Remarks to the Author):

Comments for the Author

Author identified IMPREG via clinical data analysis, linked to ICB resistance in cancers. IMPREG fosters immunosuppression by hindering antigen presentation, lowering pro-inflammatory cytokines, and depleting T cells. It's highly expressed in immune privileged organs (retina, brain, gonads), suggesting tumors exploit these mechanisms to evade immunity. IMPREG acts through malignant cells, fibroblasts, or endothelial cells in the tumor microenvironment, with expression varying by cell type and tumor. During metastasis, neuronal-like IMPREG subtypes rise while endothelial subtypes decline. In TNBC patients, high IMPREG tumors show T cell rejection and myofibroblast-rich environments. IMPREG predicts ICB response across datasets and correlates with sensitivity to targeted therapies (e.g., EGFR inhibitors, anti-angiogenics) in NSCLC. Findings support IMPREG as a biomarker and therapeutic target. Concerns remain to be addressed.

comment: *1. The background introduction of immune checkpoint blockade therapy is comprehensive, but it could further highlight the main challenges of current immunotherapy, such as low patient response rates and side effects, to emphasize the necessity and urgency of this study. When introducing the concept of immune privilege, it is recommended to supplement with specific examples of immune escape mechanisms in classical immune-privileged organs to enhance readers' understanding.*

Response: We thank the reviewer for the suggestion. We now improved the manuscript's Introduction (Lines 1-14) **to address** the low patient response rates, side effects (irAEs), **financial burden**, and the resulting **urgent need for robust biomarkers** to identify non-responders (**Page 3, second paragraph)**. We have also revised the Introduction to emphasize the limited response rates and adverse effects of current immunotherapies with examples, and added immune escape mechanisms in classical immune-privileged organs to clarify the concept (**Page 3, first paragraph)**.

Comment *2. When describing the discovery of the IMPREG signature, the analysis methods and data sources are detailed. However, it is recommended to more clearly explain why these specific analysis tools (such as Dorothea) were chosen and their advantages over other methods. In addition, when validating the predictive utility of the IMPREG signature, it is suggested to add some validation experiments at the biological mechanism level, in addition to statistical indicators (such as AUROC), to further confirm its role in immune evasion. Additionally, the spatial distribution characteristics of IMPREG-high cells in the tumor microenvironment can be explored in combination with more spatial transcriptomics data to reveal their specific localization patterns in tumor tissues.*

Response: Thank you for your constructive feedback. We have revised the manuscript to address the rationale for our methodology and strengthen the mechanistic validation of the IMPREG signature. The Methods section on signature discovery has been revised to clearly state the advantages of our chosen tools. We emphasize that the DoRothEA-VIPER approach was selected because DoRothEA is a highly curated, quantitative resource of TF regulons that maximizes confidence in the regulatory network, and VIPER is critical as it incorporates the signed mode of regulation to estimate the inferred relative protein activity, providing a more functional measure than gene expression levels (**Page 13, second paragraph)**.

Crucially, we have conducted mechanistic validation using Pan-cancer spatial transcriptomics (HEST-1k) (**Figure 5, Page 6-7, Page 13, second paragraph**). Our spatial analysis demonstrates that IMPREG+ cells actively construct an immune-privileged niche through multiple converging mechanisms:

1. **Spatial localization and immune exclusion (Fig. 5a-c):** Analysis of 278 spatially profiled tumors revealed that IMPREG-high expression concentrates in three distinct cellular compartments—myofibroblast, immature central neuronal, and endothelial subtypes. Spatial neighborhood analysis confirmed profound exclusion of effector immune cells (CD8+ T cells, M1 macrophages, NK cells) as well as Tregs and exhausted T cells from IMPREG-high regions, indicating a lack of pre-existing immunity (**Page 6, third paragraph**).

2. **Radial gradient analysis (Fig. 5d):** Quantification of pathway enrichment in concentric bands extending outward from IMPREG-high cores revealed distinct functional zones. The immediate core (0–100 μm) exhibited the lowest levels of Antigen Presentation Proficiency across all three subtypes, establishing localized hubs of immune suppression. Cell cycle and proliferation programs were enriched in distant zones, suggesting active tumor growth occurs peripherally to IMPREG-positive niches (**Page 6, 4th paragraph**).

3. **Cytokine landscape (Fig. 5e):** Spatial cytokine profiling identified TGFB3, CXCL12, IL-34, and CX3CL1 as positively correlated with IMPREG expression—factors that drive myofibroblast transition, create "stromal traps" for T cells, and maintain anti-inflammatory microglial states. Conversely, CCL15, CXCL9, and MIF were downregulated within IMPREG-high cores, reflecting active suppression of immune cell recruitment pathways (**Page 7**).

4. **Ligand-receptor interactions (Fig. 5f):** CellChat analysis revealed subtype-specific immunosuppressive mechanisms. The myofibroblast subtype employs CXCL12→CXCR4 signaling to create T-cell exclusion barriers, IGF1→IGF1R for M2 macrophage polarization, and SEMA3C→PLXND1 for ECM remodeling. The endothelial subtype utilizes TNFSF12→TNFRSF12A for angiogenesis and ANGPTL4→SDC4 to regulate vascular permeability affecting immune cell extravasation. The neuronal subtype exhibits PTN→NCL and MDK→SDC4/SDC1 axes that promote immunosuppressive niches and CD8+ T-cell dysfunction (**Page 7, third paragraph**)..

Collectively, these spatial analyses provide mechanistic validation that IMPREG orchestrates a complex, subtype-specific signaling network that simultaneously creates physical barriers, establishes biochemical traps, and actively repels effector immune cells to maintain an immune-privileged tumor microenvironment. These findings are further corroborated by multiplex IHC experimental study in our breast cancer cohort, confirming the immune-excluded phenotype.

***Comments** 3. Single-cell RNA sequencing data analysis is a crucial part of the article. When showing the distribution of IMPREG expression in different cell types, more detailed information about cell type identification markers and the basis for their selection can be provided to help readers better assess the accuracy of cell type classification.*

Response: We thank the reviewer for this suggestion. Our compendium of single-cell datasets was obtained from the Curated Cancer Cell Atlas (3CA) (Gavish et al., *Nature*, 2023; PMID: 37258682). For all datasets, we used the **original cell type annotations provided by the respective studies**, which are **re-harmonized and standardized** by the 3CA consortium (https://www.weizmann.ac.il/sites/3CA/methods). This ensures consistent cell type labels across studies and minimizes batch- or study-specific annotation biases.

Comments 4. The article identifies three IMPREG-high tumor subtypes based on cell states, but when discussing their biological significance, it is recommended to further analyze the unique mechanisms of each subtype in tumor initiation, progression, and metastasis.

Response: We appreciate the reviewer's valuable suggestion. While dissecting the roles of IMPREG in tumor initiation is beyond the scope of this biomarker-focused study, our extensive multi-modal analysis (spatial transcriptomics, scRNA-seq, and metastasis cohorts) reveals distinct mechanisms of each subtype in tumor progression and metastasis:

1. Neuronal Subtype (Metastatic Selection & Immune Checkpoint Signaling): Our analysis of 1,588 pan-metastatic samples reveals that this subtype is significantly enriched in metastatic lesions (4.9%) compared to primary tumors (2.5%), independent of tumor purity (**Figure 4f, Figure S8c-d**). This purity-independent enrichment suggests a specific biological selection for neuronal-like transcriptional programs during metastatic progression. Malignant cells in this subtype mimic fetal central neuron transcriptional states. (**please see page 6 of revise manuscript**). Spatially, these cells employ developmentally regulated signaling axes: PTN→NCL (Pleiotrophin-Nucleolin) promotes neurotrophic signaling and establishes immunosuppressive niches, MDK→SDC4/SDC1 drives M2 macrophage polarization and CD8+ T-cell dysfunction, and SPP1→CD44 functions as a direct immune checkpoint suppressing CD8+ T-cell activation (**Figure 5f**). Spatial neighborhood analysis shows enrichment of CD4 T cells within this subtype (**Figure 5b**), and it predominates in glioblastomas, neuroendocrine tumors, and prostate cancers (**Figure 4d**). (please see **page 7** of revise manuscript)

2. Myofibroblast Subtype (Physical Exclusion & Stromal Remodeling): This subtype is more prevalent in primary epithelial tumors, particularly TNBC and pancreatic adenocarcinoma. It promotes progression by orchestrating immune exclusion through extensive stromal remodeling via multiple signaling axes (**Figure 5f**): CXCL12→CXCR4 signaling from FAP+ CAFs creates a T-cell exclusion barrier while stabilizing PD-L1 expression; IGF1→IGF1R supports metabolic reprogramming and M2 macrophage polarization; GAS6→AXL mediates efferocytosis and NK cell inhibition; and SEMA3C→PLXND1 promotes CAF activation and ECM remodeling. Spatial neighborhood analysis confirms this subtype recruits immunosuppressive M2 macrophages and mast cells associated with wound healing (**Figure 5b**), fostering a fibrotic, therapy-resistant environment.

This was experimentally validated in our TNBC cohort showing strong correlation between IMPREG and stromal αSMA expression (rho = 0.55, p = 0.0005; **Figure 6e**). (please see **page 7**)

3. Endothelial Subtype (Vascular Barrier & Immune Extravasation Control): In contrast to the neuronal subtype, this subtype is significantly depleted during metastasis, decreasing from 7.4% in primary tumors to 3.4% in metastatic lesions (**Figure S8f**), suggesting its role is primarily in maintaining the primary tumor niche rather than facilitating dissemination. This subtype predominates in highly vascularized tumors such as kidney and liver-biliary cancers, where endothelial cells adopt programs resembling kidney endothelial cells to create vascular barriers excluding immune cells. Key signaling axes include (**Figure 5f**): TNFSF12→TNFRSF12A promoting angiogenesis and myeloid recruitment; IGF1→IGF1R regulating tip cell maintenance and sprouting angiogenesis while enhancing Treg/MDSC recruitment; CXCL12→CXCR4 on tumor endothelial cells inhibiting CD8+ T-cell activation; and ANGPTL4→SDC4 regulating vascular permeability to limit immune cell extravasation (please see **page 7**).

These findings demonstrate that each IMPREG subtype employs distinct molecular mechanisms to achieve immune privilege, with the neuronal subtype uniquely selected during metastatic progression while myofibroblast and endothelial subtypes predominantly maintain immune evasion in primary tumor settings.

Comments 5. When analyzing clinical trial data, in addition to focusing on treatment response and survival rates, it is suggested to perform stratified analyses based on patient clinical characteristics (such as age, gender, and tumor stage) to explore whether there are differences in the predictive value of the IMPREG signature in different patient groups. Additionally, for the application of IMPREG in predicting targeted therapy sensitivity, it is recommended to further clarify its potential clinical application scenarios and usage processes to provide more operational guidance for clinicians.

Response: We thank the reviewer for this constructive recommendation. We have incorporated stratified analyses by age, gender, and tumor stage across multiple cohorts to assess whether the predictive value of the IMPREG signature varies among patient subgroups (**Supplementary Tables S2 and S3**). We have also conducted comprehensive interaction tests (**Figure 8d**) to evaluate whether clinical variables significantly modify the predictive effect of IMPREG. Interaction testing was performed using likelihood ratio tests comparing a main effects model (Response ~ IMPREG + Modifier) to a model including the interaction term (Response ~ IMPREG + Modifier + IMPREG:Modifier). A significant likelihood ratio test indicates that the effect of IMPREG on response differs across levels of the modifier variable. Our results show that interaction terms between IMPREG and key covariates—including age, gender, smoking status, ECOG, PD-L1 expression, TMB, and clinical stage—were consistently non-significant ($p>0.05$ across tested cohorts). This indicates that the predictive accuracy of IMPREG is stable across diverse patient demographics and clinical characteristics (**please see page 8 last paragraph and page -9, first paragraph**). Additionally, we have included a section titled "Clinical Applications: Stratification for Immunotherapy and Targeted Agents" in Discussion to clarify the potential clinical applications of IMPREG, outlining how it could be integrated into patient stratification and treatment decision workflows to guide both immunotherapy and targeted therapy (**please see page 10 last paragraph**).

Comments 6. *The discussion section comprehensively summarizes and analyzes the research results. However, when comparing with other research findings, it is suggested to more deeply explore the innovation and advantages of this study, as well as the reasons for the differences from previous research results. Additionally, for the potential development direction of the IMPREG signature in future research, it is recommended to propose more forward-looking and innovative suggestions, such as exploring its application value in combination therapy strategies.*

Response: We thank the reviewer for the suggestion. We have revised the Discussion to highlight the novelty and advantages of IMPREG compared with previous studies, explain observed differences, and propose future directions, including its potential use in combination therapy strategies (**page 10, first paragraph**).

Comments 7. The article mentions some research limitations in the discussion section, such as data heterogeneity, but it is recommended to further elaborate on the potential impact of these limitations on the research results and how to overcome them in future research.

Response: We thank the reviewer for the suggestion. We have expanded the Discussion to clarify how data heterogeneity and cohort variability may influence the generalizability of our findings and outlined future strategies—such as prospective validation and integration of multi-omics and spatial data—to mitigate these limitations. (**page 11, second paragraph**).

Reviewer #4 (Remarks to the Author):

In this manuscript, the authors present a pan-cancer computational analysis of transcriptional pathways associated with immune escape and the lack of response to immunotherapy. Their main claim is the development of a gene-set signature based on transcription factor (TF) regulons that are negatively associated with immune responses, indicating a role in immune evasion and resistance to immunotherapy. Interestingly, the signature appears to be linked to responses to anti-angiogenic therapies and EGFR inhibitors.

While the analysis supports the enrichment of the gene set in tumors with poor immunotherapy responses, its uniqueness and comparison to existing gene sets and scores remain unclear. Additionally, it is uncertain whether this represents a distinct biological entity or if it is capturing a more general phenomenon, which, while clinically relevant, may not constitute a truly novel biological category.

Response: We thank the reviewer for this insightful comment. Our multi-modal analyses demonstrate that IMPREG represents a distinct biological entity and is orthogonal to canonical gene sets and biomarkers:

1. Mechanistic Grounding in Immune-Privilege Physiology (Figure 2): Unlike post-hoc outcome-derived signatures, IMPREG is rooted in immune-privilege biology. GTEx analysis shows IMPREG is markedly elevated in immune-privileged tissues (retina, brain, CNS) and absent in T-cell-rich organs (spleen, liver) (**Figure 2a-b**). Pathway analysis confirms IMPREG defines a specific "immune-privilege" program—upregulating neuronal development and stromal-vascular remodeling while suppressing interferon responses and antigen presentation (**Figure 2c**).

2. Orthogonality to Canonical Programs (Figure 8b-c, S15): Benchmarking against 16 canonical features (Usset et al., Nat Genet. 2024) revealed IMPREG as a standalone node, not clustering with the five established programs (proliferation, immune activation, EMT/TGF-β, prior therapy, TMB) (**Figure 8b**). PCA confirmed IMPREG as the primary PC1 contributor (29% variance), with its vector opposing T-cell effector, TMB, pan-fibroblast TGF-β, and proliferation axes (**Figure 8c**)—underscoring its role as a novel orthogonal axis capturing active immune-privilege co-option rather than simple lack of immunogenicity (**Page 8, paragraph 3**).

3. Non-Redundant Predictive Power (Figure 8a, e-f): IMPREG outperformed established immunotherapy resistance signatures (MDSC, EMT, TGF-β) and response signatures (IFN-γ, T-effector) across 40 datasets/14 cancer types (**Figure 8a**). Across harmonized TMB-annotated datasets using author-reported TMB values from the original publications, multivariable Cox regression identified IMPREG as the strongest predictor over TMB, CD274, and established signatures (**Figure 8e**). Additive modeling confirmed that IMPREG contributed predictive information beyond established biomarkers (**Figure 8f**), consistently improving model fit across all marker combinations (**page 8, last paragraph**).

Collectively, these findings establish IMPREG as a mechanistically distinct and non-redundant biomarker that captures active immune-privilege co-option, representing a previously unrecognized biological program orthogonal to canonical immunotherapy resistance signatures.

Response: We thank the reviewer for this insightful comment. We have expanded our analysis to demonstrate that IMPREG represents a distinct biological entity rather than a proxy for generalized immune suppression.

1. **Orthogonality to Canonical Programs (Figure 8):** Benchmarking against 16 canonical latent tumor programs (Usset et al., 2024) reveals that IMPREG forms a standalone cluster distinct from other latent

variables such as EMT, TGF-β and T-effector signatures (**Figure 8b**). PCA confirms this orthogonality, identifying IMPREG as the primary contributor to PC1 (29% variance) and distinct from canonical programs such as proliferation or TMB axes (**Figure 8c, Figure S15, Page 8**).

2. **Distinctness from TGF-β and EMT (Figure 8e, S14):** IMPREG consistently outperforms the "Pan-fibroblast TGF-β response" and EMT signatures in predicting resistance (**Figure 8a**). Furthermore, correlation analyses (**Figure 8b**) and variance loading analyses (**Figure S15**) confirm that IMPREG provides independent, additive predictive value, indicating non-redundant biological mechanisms.
3. **Unique Biological Architecture (Figure 2c, page 4):** Pathway analysis confirms that IMPREG captures a specific "immune-privilege" program defined by neuronal (e.g., nervous system development) and stromal-vascular remodeling pathways, fundamentally distinguishing it from generalized immune-suppressed states characterized merely by the absence of inflammation.

Comments: 1)*What is unique about IMPREG and how does it compare to other gene-sets? I would suggest: - Conducting a systematic comparison with existing gene sets (e.g., MSigDB, Reactome, TGF-β-related gene sets) to contextualize IMPREG's distinct features.*

Response: We thank the reviewer for this suggestion. We now provide a systematic contextualization of IMPREG relative to existing gene sets and pathways.
1. We first performed MSigDB Hallmark and Canonical/Reactome pathway enrichment of IMPREG target programs using the IndepthPathway/CSEA framework, revealing enrichment of neuronal and stromal-vascular remodeling pathways with concomitant suppression of interferon/cytokine signaling and antigen-presentation programs (**Fig. 2c, Page 5**).
2. Beyond predefined pathways, we conducted an unbiased correlation of IMPREG with 1,265 curated cell-state signatures in single cell and bulk RNAseq datasets, identifying myofibroblast, immature neuronal, and endothelial programs that form the basis of IMPREG subtyping (**Fig. 4a; Fig. S7, Page 6 paragraph 1**).
3. We benchmarked IMPREG against ten established MSigDB/Reactome-derived immune and stromal signatures (i.e., T-cell effector, antigen presentation, EMT, and pan-fibroblast TGF-β response) across 40 independent immunotherapy cohorts, where IMPREG consistently outperformed these signatures for predicting resistance (**Fig. 8a, Page 8, paragraph 3**).
4. Orthogonality analyses demonstrated that IMPREG is not reducible to canonical immune, TGF-β, EMT, TMB, or T-cell–inflamed programs, but instead represents an independent axis of tumor immune privilege (**Fig. 8b–c, Page 8, paragraph 3**).
Collectively, these analyses establish IMPREG as a distinct, non-redundant transcriptional program rather than a re-labeling of existing gene sets.

Comments: Assessing the added value of using an aggregated score versus individual regulons. Can the contribution of each component be broken down to understand how they influence the overall score across cancer types? Can you do this analytically?

Response: We thank the reviewer for this important question. We conducted three complementary analyses to quantify the added value of aggregation and analytically dissect regulon contributions across cancer types.

1. Performance benchmarking. The full IMPREG signature consistently outperformed all individual regulons and subtype-restricted versions (neuronal-, fibroblast-, endothelial-IMPREG) across 40 independent clinical cohorts (**Figure S6A**). While individual regulons occasionally achieved high AUROC in specific contexts, none maintained stable predictive performance across the full spectrum of cancer types and treatments (**Page 5 Last paragraph**).

2. Independent predictor analysis. Multivariable LASSO regression identified which regulons retain independent predictive value within each cohort (**Figure S6B**). The results revealed that while specific regulons drive resistance in context-dependent manners, no single regulon consistently predicts response across all 40 independent cohorts. This directly demonstrates why aggregation is necessary—resistance mechanisms operate through different regulatory nodes depending on tumor context (**Page 5 Last paragraph**).

3. Variance decomposition. We apply standardized linear model coefficients to quantify each regulon's contribution to the composite score (**Figure S6C**). The ten regulons collectively explained nearly all IMPREG variance (median $R^2 \approx 0.99$; Table S1), confirming a coherent regulatory module. ZNF683 and ETV7 emerged as consistently strong contributors, aligning with their established roles in tissue-resident immunity and interferon signaling. Notably, their substantial contribution to score variance did not confer universal independent predictive value (**Figure S6B**), illustrating that regulons anchoring biological coherence still require integration with context-adaptive components for robust prediction (**Page 5 Last paragraph**).

Conclusion. IMPREG's architecture reflects how biological systems achieve robustness: core regulons (ZNF683, ETV7) provide a stable immunoregulatory foundation, while context-adaptive regulons (NME2, NCOA family, SOX2) calibrate performance across tumor microenvironments. The aggregated score captures emergent predictive information unavailable from any individual component, offering significant added value for pan-cancer clinical application.

Comments 2)*While the association with negative immunotherapy responses is clear, it remains unclear how IMPREG compares to other molecular features linked to poor immunotherapy outcomes. Specifically, what is its added value relative to tumor mutational burden (TMB) or T-cell infiltration levels (high vs. low)?*

Response: We thank the reviewer for the suggestion. We have included comparative analyses to demonstrate the added value of IMPREG. As not all datasets have TMB data, we separated the analysis into two cohorts: 1) an expanded compendium of 40 immunotherapy clinical trial datasets, and 2) a subset of 12 ICB trials with available TMB data.

1. **Comparative Performance (Figure 8A, Supplementary Fig. 14):** In the expanded compendium, IMPREG consistently outperforms 10 established gene expression predictive signatures in predicting immunotherapy outcome across 40 datasets (**Page 8, 3rd paragraph**).
2. **Independent Predictive Value (Figure 8e):** In the ten ICB trials with available TMB data, multivariable regression models confirm that IMPREG remains a significant independent predictor of non-response even after adjusting for TMB, CD274 (PD-L1) expression, IFN-γ, CD8 T effector, and pan-fibroblast TGF-β signatures. This indicates that IMPREG captures a distinct mechanism of resistance that is not accounted for by mutational load or established inflammation signatures (**Page 8 last paragraph, and page 9 first paragraph**).

Comments *- Is IMPREG an orthogonal marker, or does it represent a sixth latent factor (see PMID: 39266764)? –Additionally, are other gene sets associated with negative immunotherapy responses also linked to increased sensitivity to anti-angiogenic therapies or EGFR inhibitors*? Or is this feature unique to IMPREG?

Response:

1. Orthogonality: To rigorously quantify this, we performed correlation-based clustering and Principal Component Analysis (PCA) (**Figure 8b-c**). IMPREG formed a distinct cluster with low or negative correlations to the five canonical latent factors (TMB, Proliferation, EMT/TGF-β, etc.) (**Figure 8b**). Notably, in the PCA biplot, IMPREG emerged as the primary contributor to PC1 (29% variance) and projected orthogonally to T-effector and TMB vectors (**Figure 8c, S15**), confirming it captures a unique source of biological variance not redundant with existing programs (**Page 8**).

2. Uniqueness of IMPREG's predictive value for targeted therapy. We benchmarked IMPREG against established immunotherapy resistance signatures (MDSC, TGF-β, EMT) across 19 targeted therapy clinical trials (**Figure 9a**). IMPREG exhibited a unique bidirectional predictive pattern: it predicted sensitivity to EGFR-targeted therapies (erlotinib, osimertinib, vandetinib) in NSCLC as well as to VEGF-targeted monotherapies (bevacizumab, sunitinib, sorafenib) across multiple cancer types. No other signature demonstrated this discriminatory capacity. Notably, when bevacizumab was combined with chemotherapy (CRC, TNBC), IMPREG showed diminished association, suggesting the predictive value is specific to

antiangiogenic therapy. This indicates that the sensitivity to these targeted agents is a unique feature of the IMPREG phenotype and not a general property of immune-resistant tumors.

Comments 3) I would finally recommend to focus the question of the manuscript (more clinical applicability or biological novelty) and weight the manuscript accordingly.

Response: We thank the reviewer for this suggestion. The revised manuscript now explicitly centers on IMPREG's **dual clinical utility** as a predictive biomarker:

1. Predictive Biomarker for ICB Resistance: IMPREG consistently predicts non-responders across 4 discovery and 36 validation datasets spanning 10 cancer types (Average AUC 0.71), enabling identification of patients at risk for futile treatment, hyper-progressive disease, and severe toxicities.

2. Redirectional Biomarker for Targeted Therapy: Uniquely among immunotherapy biomarkers, IMPREG-high tumors show enhanced sensitivity to EGFR inhibitors (AUC 0.72–0.81 in NSCLC trials, independent of mutation status) and anti-angiogenic agents in RCC, HCC, and CRC.

We added a "Clinical Applications" Discussion section outlining an IMPREG-guided decision framework for treatment stratification, positioning biological findings as mechanistic support for these applications (**Page 10-11**, last paragraph).

Response to Reviewer #4

We sincerely thank the reviewer for the constructive feedback throughout the review process and are pleased that the reviewer finds IMPREG to represent a distinctive and clinically relevant fingerprint for predicting immunotherapy response across cancer types. Below, we address the remaining comment in detail.

Reviewer Comment:

"In Figure S7, I would like to see the similarity distribution of all programs compared against the full set of 1,265 programs, with an indication of where the three main contributors (fibroblast, neuronal, and endothelial programs) fall within this overall distribution. In its current form, the figure lacks sufficient context, and it is unclear whether these programs are indeed among the most similar ones or were selectively highlighted."

Response: We thank the reviewer for this valuable suggestion. We have replaced Supplementary Figure S7 to address this concern. The new figure now displays all 1,265 curated cell-state signatures simultaneously as scatter plots, one for each IMPREG subtype (endothelial, neuronal, and myofibroblast). Each point represents a single signature, positioned by its trimmed mean Pearson correlation with IMPREG in pan-cancer scRNA-seq datasets within the indicated cellular compartment (y-axis) and its correlation with IMPREG in TCGA pan-cancer bulk RNA-seq data (x-axis). This provides the full distributional context the reviewer requested, making the position of each selected signature visible relative to all 1,265 signatures.

To objectively distinguish subtype-specific from non-specific signatures, we performed two-sided t-tests comparing each signature's IMPREG correlation in the index subtype versus all other subtypes. Only signatures achieving $p < 0.01$ were considered subtype-specific. Points are color-coded accordingly: grey for non-significant signatures, orange for significant positive associations, and green for significant negative associations with IMPREG. Compartment-matched, lineage-consistent signatures (e.g., endothelial-derived signatures in the endothelial subtype) are highlighted in purple with black borders, and the top five are labeled in each panel. The first-ranked signature among these, indicated by a bordered label, was selected as the representative subtype marker. This presentation demonstrates that the three selected signatures emerged as top-ranked lineage-consistent markers through a systematic, data-driven selection process. The complete results for all 1,265 signatures — including trimmed mean correlations, t-test statistics, and p-values per IMPREG subtype — are provided in the Source Data for independent verification.
